# Synthesis, Structure, Electrochemistry, and In Vitro Anticancer and Anti-Migratory Activities of (*Z*)- and (*E*)-2-Substituted-3-Ferrocene-Acrylonitrile Hybrids and Their Derivatives

**DOI:** 10.3390/molecules30132835

**Published:** 2025-07-02

**Authors:** William O. Mendoza-Morales, Esteban Rodríguez, Aliana González, Zulma Ramos, Jemily Acosta-Mercado, Dalice M. Piñero-Cruz, Claudia A. Ospina, Enrique Meléndez, Eliud Hernández-O’Farrill

**Affiliations:** 1 Department of Pharmaceutical Sciences, School of Pharmacy, University of Puerto Rico, San Juan 00936, Puerto Rico; william.mendoza1@upr.edu (W.O.M.-M.); esteban.rodriguez15@upr.edu (E.R.); zulma.ramos1@upr.edu (Z.R.); 2 Department of Natural Sciences and Mathematics, Interamerican University of Puerto Rico, Bayamon Campus, Bayamon 00957, Puerto Rico; agonzalez6977@interbayamon.edu (A.G.); cospina@bayamon.inter.edu (C.A.O.); 3 Department of Chemistry, College of Natural Sciences, University of Puerto Rico, Rio Piedras Campus, San Juan 00925, Puerto Rico; jemily.acosta@upr.edu (J.A.-M.); dalice.pinero@upr.edu (D.M.P.-C.); 4 Department of Chemistry, University of Puerto Rico, Mayagüez Campus, Mayagüez 00681, Puerto Rico; enrique.melendez@upr.edu

**Keywords:** ferrocene, acrylonitrile, cancer, migration, electrochemistry

## Abstract

In this study, a series of (*Z*)- and (*E*)-2-substituted-3-ferrocene-acrylonitrile derivatives were synthesized, characterized, and evaluated in vitro for their anticancer and anti-migration properties. The compounds were synthesized via the Knoevenagel condensation of the appropriate benzyl cyanide or benzoyl acetonitrile with ferrocenecarboxaldehyde 1, producing isolated yields of 99 to 23%. The structures of the compounds were analyzed using IR, ^1^H NMR, ^13^C{^1^H} NMR, GC-MS, and UV/Vis spectroscopic methods. Single-crystal X-ray diffraction analysis of representative compounds **21**, **27**, and **29** demonstrated that the geometry of the double bond was that of the (*Z*)-isomer. For representative compound **33,** the geometry of the double bond was that of the (*E*)-isomer. Additionally, the electrochemistry of the compounds was investigated using cyclic voltammetry. The cytotoxic and anti-migratory effects of these compounds were evaluated in the MCF-7 and MDA-MB-231 breast cancer cell lines, providing insight into the structure–activity relationships. Preliminary investigations of their anticancer activity revealed that several compounds exhibit moderate antiproliferative effects on cancer cell lines, with GI_50_ values ranging from 23 to 44 μM for the MCF-7 cell line and from 9 to 41 μM for the MDA-MB-231 cell line. Moreover, compound (*Z*)-**25** inhibited 13% of the migration activity of the metastatic MDA-MB-231 cell line.

## 1. Introduction

**Figure 1:**
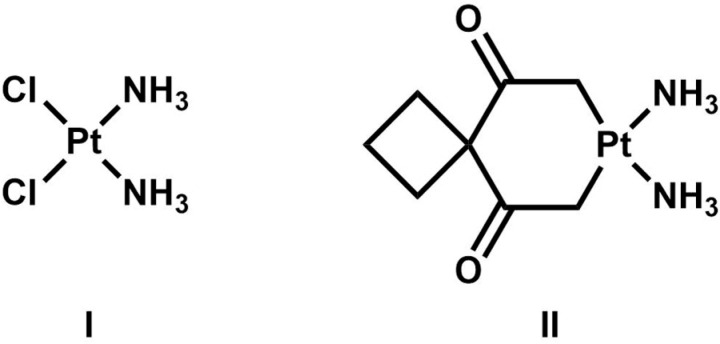
Structure of representative anticancer platinum derivatives cisplatin (**I)** and carboplatin (**II)**.

Cancer remains one of the most commonly diagnosed diseases worldwide, with approximately 20 million cancer cases diagnosed by 2022 [1]. The number of cancer cases is predicted to increase to approximately 35 million by the year 2050. The rate of new cases of overall cancer incidence per 100,000 person-years is 213 in males and 186 in females. Breast cancer (BC) is one of the most common types of cancer among women and represents 15% of all new cancer cases in the U.S. The standard treatment strategy for BC patients with HER2+ or TNBC is based on a multimodal approach, including trastuzumab (Herceptin), taxane-based therapy, mastectomy, radiation therapy, and targeted therapy [2]. Among the alternatives to targeted treatment for cancer, metal-based anticancer drugs have garnered attention over the last four decades [3]. The well-known drug cisplatin [*cis*-diamminedichloroplatinum (II)] and its derivative carboplatin [*cis*-diammine-1,1-cyclobutanedicarboxylatoplatinum (II)] are platinum coordination complexes with a square planar geometry that are used in 50% of cancer treatments (Figure 1) [4]. Cisplatin is administered intravenously and can be used alone or in combination with other chemotherapeutic agents for treating early-stage and advanced breast cancer. Once the drug reaches cancer cells, it enters by passive diffusion as a neutral compound and accumulates intracellularly. Cisplatin is activated inside the cell through the hydrolysis of the Pt-Cl bonds that lead to the formation of the reactive platinum complex *cis*-[Pt(NH_3_)_2_(OH_2_)_2_]^2+^. The primary mechanism of action of cisplatin and carboplatin involves binding the Pt atom to the highly nucleophilic N7 atoms of guanine or adenine purine bases on DNA, forming intrastrand cross-links between bases, which interfere with gene transcription and DNA replication mechanisms [5]. The primary mode of anticancer activity of these drugs is initiating a cytotoxic effect by targeting the cell division process. However, three major drawbacks to their use are their binding to non-DNA targets, ineffectiveness against platinum-resistant tumors, and nephrotoxicity [6]. Therefore, new metallic-based compounds are being investigated as potential anticancer drugs with fewer side effects.

**Figure 2:**
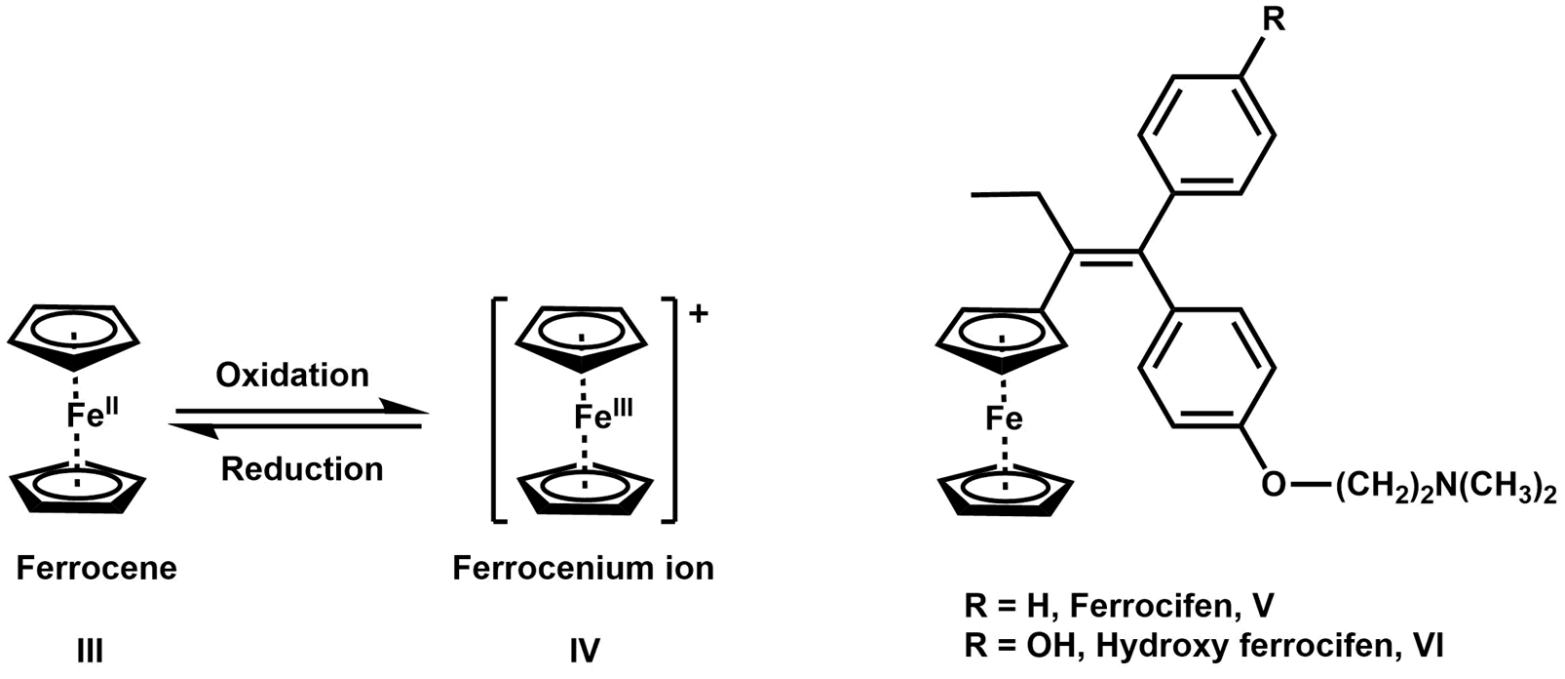
Structure of ferrocene (**III)** and ferrocenium ion (**IV)**, and representative anticancer ferrocene derivatives ferrocifen (**V)** and hydroxy ferrocifen (**VI)**.

Metallocenes are a class of organometallic compounds that are widely used in catalysis-mediated processes, biosensors, and medicinal applications. Kealy and Pauson discovered the first metallocene, ferrocene, in 1951, and Fischer and Wilkinson’s subsequent structural analysis marked the beginning of a new era in organometallic chemistry [7]. Metallocenes, such as ferrocene (Fc) **III**, are organometallic compounds consisting of two parallel cyclopentadienyls (Cp) anions bound to a metal center; through each metal center, a hapticity of η^5^ (eta) bonds forms a very stable “sandwich-like” complex with the two Cp π-bonded anions and the positively charged metal atom (Figure 2) [8]. In the electronic structure of ferrocene, the primary orbital interactions that form the Fe-(Cp)_2_ bonds occur between the Fe *d* orbitals and the π-orbitals of the Cp ligands. Each Cp ligand forms five *p*-orbitals oriented perpendicular (*p_z_*) to the five-carbon ring, which combine to produce five molecular orbitals with a suitable symmetry to overlap with the Fe 3*d_xz_* and 3*d_yz_* orbitals [9]. This is the orbital symmetry combination in the Cp rings that allows the Fe 3d orbitals to interact efficiently, and it is mainly responsible for the stability of the complex. Ferrocene has a pseudo-octahedral structure and is highly stable in air, water, and in the presence of strong bases, as well as at high temperatures of up to 460 °C [10]. Moreover, ferrocene is lipophilic and has two accessible oxidation states under physiological conditions [Fc, Fe(II); Fc^+^, Fe(III)], which are essential features that influence its behavior in biological systems. The redox characteristics of ferrocene enhance its ability to induce ferroptosis in cancer cells [9,10]. In the presence of an oxidizing agent, Fc can undergo reversible single-electron oxidation to form Fc^+^ (ferrocenium ion) **IV**. When reacting Fc with hydrogen peroxide, the single-electron oxidation of Fc to Fc^+^ produces hydroxyl radicals (HO^−^) and other reactive oxygen species (ROS) via the Fenton mechanism [11]. This mechanism is primarily responsible for the cytotoxicity of some ferrocene derivatives, as the highly reactive nature of hydroxyl radicals and the generation of intracellular reactive oxygen species (ROS) can interfere with cellular processes by reacting with DNA, lipids, RNA, and proteins, ultimately initiating apoptosis in cancer cells [12].

Each cyclopentadienyl anion ligand in the ferrocene complex is aromatic and exhibits characteristics similar to benzene rings [13,14,15]. Due to its chemical properties, including lipophilicity and stability, it is logical to use ferrocene in ring bioisosterism, through which, phenyl rings in bioactive agents are replaced [15]. Ferrocene itself is not a very toxic compound. A study reported that daily oral administration of 300 mg/kg for 3 months resulted in hemosiderosis, a condition characterized by the abnormal accumulation of iron. However, no adverse effects were observed in dogs with high levels of iron that were maintained for 12–26 months after a 6-month treatment [16]. However, some major metabolic conversions of the phenyl rings in the liver were observed [17]. The low toxicity of ferrocene may be attributed to its structural stability and the well-protected iron atom, which is buried in the sandwich geometry formed by the two cyclopentadienyl (Cp) rings. The Fc/Fc^+^ redox change is fast, and different solvents do not affect the rate [18]. Furthermore, the one-electron removal during the redox change does not affect the compound’s sandwich geometry, and only a slight change in the Fe-C bond elongation is observed because the electron is removed from the nonbonding *d*_xy_ molecular orbital.

The application of ferrocene and its derivatives in cancer treatment began to gain attention after it was established that their cytotoxic activity is primarily mediated through a redox mechanism [19]. One of the most studied ferrocene derivatives is the anticancer drug candidate ferrocifen (Figure 2). This compound is a tamoxifen (Tam) analog that is currently in advanced preclinical evaluation against hormone-dependent and hormone-independent breast cancer cells [20]. Tamoxifen is a first-line chemotherapeutic for patients with hormone-dependent (estrogen receptor α-positive (ERα^+^)) breast cancer [20,21]. After oral administration, Tam is metabolized to hydroxytamoxifen (HO-Tam), an active metabolite that competes with β-estradiol for binding to estrogen receptor α (ERα) [22]. HO-Tam is more potent than its parent compound against ERα^+^ tumors; it functions by inducing cell death through the suppression of DNA transcription. To combine the antiproliferative effects of ferrocene with the activity of Tam, the ferrocene structure was bioisosterically incorporated into Tam, replacing the phenyl group. Ferrocifen (Fc-Tam) **V** and its analog hydroxy ferrocifen (HO-FC-Tam) **VI** were found to be more potent than Tam and HO-Tam (Figure 2) [23]. Moreover, they were more active against ERα^+^ and hormone-independent (ERα^−^) breast cancer cells. It was proposed that the mechanism of action of ferrocifen derivatives starts with the binding to ERα by the more active *Z*-isomer, followed by a loss of 2e^−^ and 2H^+^ to produce a *para* quinone methide intermediate that is stable under physiological conditions [24]. The ferrocene moiety was proposed to act as an electron relay in this process. The quinone methide intermediate is a reactive 1, 8 Michael acceptor that is susceptible to nucleophilic attack from endogenous glutathione or nucleobases, thereby contributing to the overall cytotoxic effect against breast cancer cells [25]. Additionally, ferrocifen derivatives have been found to produce reactive oxygen species (ROS), especially hydroxy radicals (HO˙), in specific cell lines [26].

α, β-unsaturated nitriles, also known as acrylonitriles, have been incorporated into various chemical structures, including medicinal agents, agrochemicals, and functionalized materials [27,28]. The versatility of the acrylonitrile group has been attributed to its flexibility, stability, and ability to incorporate functionalized alkyl or aryl groups in the 2- and 3-positions [29]. When highly substituted, the acrylonitrile unit can be obtained as both *Z*- and *E*-isomers, thereby contributing to the difference in the biological activity of compounds with this functionality [30]. Herein, we designed and synthesized a new series of (*Z*)- and (*E*)-2-aryl-3-ferrocene-acrylonitrile derivatives, which were characterized using UV/Vis spectroscopy, cyclic voltammetry, and X-ray diffraction experiments. All compounds were tested for their antiproliferative and anti-migratory activities in the MCF-7 and MDA-MB-231 breast cancer cell lines.

## 2. Results and Discussion

This study designed and synthesized a new series of (*Z*)- and (*E*)-2-aryl-3-ferrocene-acrylonitrile derivatives and analyzed their cytotoxic effect and potential to inhibit cancer cell migration. The structural elements identified as pharmacophoric units were ferrocene, the α, β-unsaturated nitrile unit, and substituted aryl or aroyl phenyl rings. Our strategy was to design and synthesize a new series of compound derivatives of 2-aryl- or 2-benzoyl-3-ferrocene-acrylonitriles. The influence of aromatic substituents on the 2-position, the Z/*E* structure geometry, and the 3-substituted-ferrocene-acrylonitrile unit as a scaffold was examined.

All compounds were synthesized with yields ranging from 99% to 23%, and their structures and purities were confirmed by ^1^H NMR, ^13^C{^1^H} NMR, IR, GC-MS, and UV/Vis spectroscopic analyses. The geometry of the double bond was established from single-crystal X-ray crystallographic data. Additionally, the electrochemistry of the ferrocene–acrylonitrile derivatives was investigated using cyclic voltammetry. We screened all the compounds to determine their cytotoxic effects against MCF-7 and MDA-MB-231 breast cancer cells using the Sulforhodamine B (SRB) assay [31]. In addition, the anti-migratory activity was determined using the wound-healing assay (scratch method) [32] and metastatic MDA-MB-231 cells. In this assay, the relative migration of MDA-MB-231 breast cancer cells in the presence of ferrocene–acrylonitriles at a concentration of 10 µM (or at concentrations that do not affect cell viability) was compared to the migration in the presence of the vehicle (0.02% DMSO). Representative micrographs of compound **25** are represented. The results showed that wound healing in the vehicle-control experiment progressed rapidly, and after 24 h, the wound was completely healed. When the cells were incubated with compound (*Z*)-**25**, wound healing was inhibited. The structure, UV/Vis absorption data, cyclic voltammetry values, and biological activity of all the compounds are summarized in Table 1, Table 2, Table 3, Table 4 and Table 5.

### 2.1. Synthesis and Structural Analysis

**Scheme 1:**
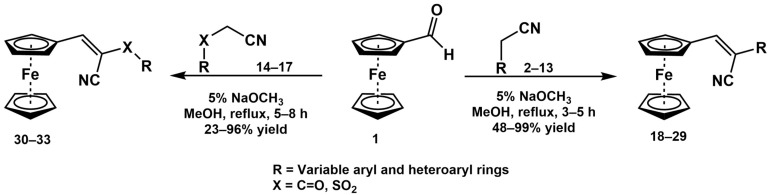
General synthesis procedure for 2-substituted-3-ferrocene-acrylonitrile derivatives **18–33**.

Scheme 1 describes the synthesis methods for constructing the 2-aryl-3-ferrocene-acrylonitrile derivative library (see the Supplementary Materials for representative ^1^H and ^13^C{^1^H} NMR spectral data). All compounds were synthesized through Knoevenagel condensation of the appropriate benzyl cyanide or benzoyl acetonitrile with ferrocenecarboxaldehyde. Ferrocenecarboxaldehyde **1** was refluxed with the appropriate acetonitrile **2**–**17** in a 5% sodium methoxide/methanol solution for 3–5 h to produce the corresponding 3-ferrocenyl-2-arylacrylonitriles **18**–**29**, 3-ferrocenyl-2-aroylacrylonitriles **30**–**32**, and 3-ferrocenyl-2-(phenylsulfonyl)acrylonitrile **33**, respectively. The resulting crude product was diluted with water, extracted with ethyl acetate, and purified via flash column chromatography over silica gel to obtain the final product in yields ranging from 23% to 99% (Table 1). It should be noted that (*Z*)-3-ferrocenyl-2-substituted-acrylonitrile derivatives **18**, **19**, **20,** and **28** have been previously synthesized (via a solvent-free synthetic protocol in the presence of piperidine) and were reported in [33,34,35]. Although the isolated yields for compounds **18** and **19** were not reported, the reported yields for compounds **20** and **28** were 6% and 91%, respectively. However, their biological activity was not reported. The base-catalyzed condensation of aryl or heteroaryl aldehydes with aryl or heteroaryl acetonitriles typically only yields the Z-isomer. However, some reaction products were obtained as a mixture of *Z* and *E* isomers (compounds **26** and **28** were obtained with *Z*:*E* ratios of 99:1 and 98:2, respectively). However, the isomers were separable and the biological activity of both isomers could be evaluated. The structure and purity of the products were confirmed by the analysis of the ^1^H and ^13^C{^1^H} NMR, FTIR, and GC-MS spectral data. In the FTIR spectra of all of the compounds, the nitrile C≡N absorption showed a sharp stretching signal of medium intensity at a frequency ranging from 2213 to 2206 cm^−1^, which is a typical frequency for α, β-unsaturated nitriles and represents an approximately 50–40 cm^−1^ shift to a lower frequency compared to the nitrile C≡N absorption of saturated nitriles (2260–2240 cm^−1^).

**Table 1:** Structure representation of synthesized 2-substituted-3-ferrocene derivatives and the corresponding acetonitrile.

Substrate	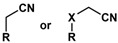 R = Aryl and Heteroaryl Rings X = C=O, SO_2_	Product	2-Substituted-3-Ferroceneacrylonitrile	Yield (%)
**2**	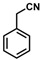	(*Z*)-**18**	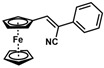	98
**3**	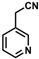	(*Z*)-**19**	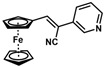	77
**4**	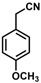	(*Z*)-**20**	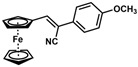	59 ^b^
**5**	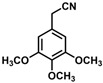	(*Z*)-**21**	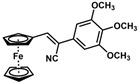	88
**6**	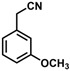	(*Z*)-**22**	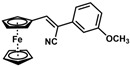	61
**7**	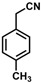	(*Z*)-**23**	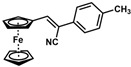	89
**8**	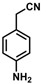	(*Z*)-**24**	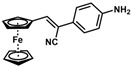	88
**9**	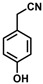	(*Z*)-**25**	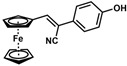	48
**10**	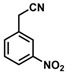	(*Z*)-**26** ^a^	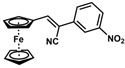	70
**11**	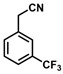	(*Z*)-**27**	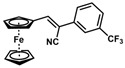	99
**12**	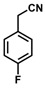	(*Z*)-**28** ^a^	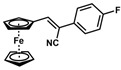	87 ^b^
**13**	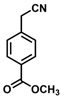	(*Z*)-**29**	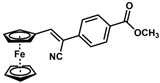	75
**14**	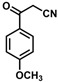	(*E*)-**30**	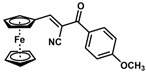	23
**15**	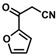	(*E*)-**31**	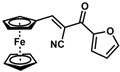	57
**16**	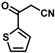	(*E*)-**32**	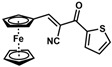	96
**17**	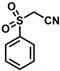	(*E*)-**33**	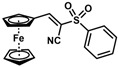	73

^a^ Reaction product was obtained as a mixture of *Z* and *E* isomers at ratios of 99:1 and 98:2 for compounds **26** and **28**, respectively. ^b^ The isolated yields reported for (*Z*)-**20** and (*Z*)-**28** are 6 and 91%, respectively [30]. For compounds (*Z*)-**18** and (*Z*)-**19**, the isolated yields are not reported.

**Scheme 2:**
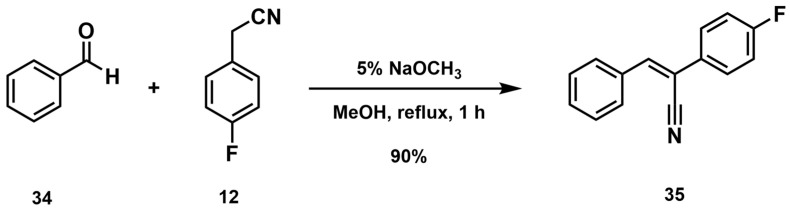
Synthesis of (*Z*)-2-(4-fluorophenyl)-3-phenylacrylonitrile **35**.

Each cyclopentadienyl ligand in the ferrocene complex is aromatic and exhibits characteristics similar to benzene rings. Therefore, to determine whether the replacement of the ferrocenyl moiety with a benzene ring would retain activity, for example, compared to compound **28**, the (*Z*)-2-(4-fluorophenyl)-3-phenylacrylonitrile **35** was synthesized by reacting equimolar amounts of benzaldehyde **34** with the corresponding 4-fluorophenylacetonitrile **12** refluxed in a 5% sodium methoxide/methanol solution for one hour, as shown in Scheme 2. Compound **35** was obtained with a 90% yield after purification of the crude material via flash column chromatography over silica gel. It should be mentioned that compound **35** has been previously reported [36]. The yield was reported as 95% after crystallization in ethanol, which is comparable to the yield that we obtained. However, its biological activity was not reported.

#### 2.1.1. Structure Determination

**Figure 3:**
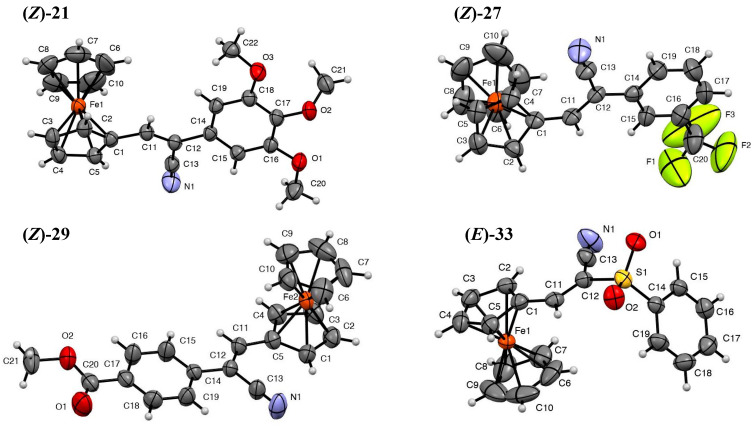
Molecular structures of (*Z*)-**21**, (*Z*)-**27**, (*Z*)-**29**, and (*E*)-**33**. Thermal ellipsoid plots (50%, 300 K) with labels.

The condensation of ferrocenecarboxaldehyde **1** with an acetonitrile (**2–13**) in 5% sodium methoxide in methanol mainly formed the (*Z*)-isomer (**18**–**29**). However, the condensation product using acetonitriles **14**–**17** formed the (*E*)-isomer of the 3-ferrocene-acrylonitrile analogs **30**–**33**. To confirm the (*Z*)- or (*E*)-configuration of the double bond, representative compounds **21**, **27**, **29**, and **33** were subjected to single-crystal X-ray diffraction analysis. The results showed that compound **21** crystallizes in the triclinic space group P-1, and **27** crystallizes in the monoclinic space group Pc. The substituent in the ferrocene subunit lies co-planar to the cyclopentadienyl ring. Compound **29** was found to crystallize in the monoclinic space group P21/c, while compound **33** was found to crystallize in the monoclinic space group P21/n. As shown in Figure 3, the geometry of the double bond of compounds **21**, **27**, and **29** was that of the (*Z*)-isomer, and the geometry in compound **33** was that of the (*E*)-isomer. In structures **21**, **27**, and **29**, we observed that the aryl ring and the double bond are coplanar with the cyclopentadienyl ring. Important intermolecular interactions were observed in structure **27** between nitrogen N1 from the nitrile substituent and C11 and C15, as well as between C7 … C35 from the C_6_H_4_CF_3_ substituents. The CF_3_ groups tend to undergo molecular rotation, leading to positional disorders in the structure (Figure S22, Supplementary Material). In the case of structure **33**, both oxygen atoms of the sulfonyl group are also coplanar with the double bond of the acrylonitrile moiety. Moreover, the two cyclopentadienyl rings in the four structures have an eclipsed conformation in their single-crystal form.

#### 2.1.2. UV/Vis Absorption

**Figure 4:**
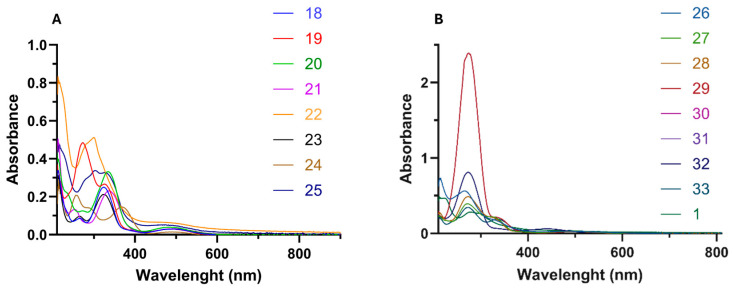
(**A**) Representative UV/Vis absorption spectra of 2-substituted-3-ferroceneacrylonitrile derivatives **18–25**. (**B**) Representative UV/Vis absorption spectra of 2-substituted-3-ferroceneacrylonitrile derivatives **26**–**33** and ferrocenecarboxaldehyde **1** in acetonitrile (10^−^⁵ M).

The UV/Vis absorption spectra of 2-substituted-ferrocene-acrylonitriles **18–33** were recorded in acetonitrile and are shown in Figure 4, and the data are compiled in Table 2. The UV/Vis spectra for ferrocene consist of three main absorptions: one absorption for d-d* transitions within the Fe atom at 440 nm (Vis region, electromagnetic radiation with wavelengths between 380 and 780) with a low intensity; a second, high-intensity absorption for n–π* transitions corresponding to the metal-to-ligand charge-transfer (MLCT) at 325 nm of the UV-A range (electromagnetic radiation with wavelengths between 320 and 380 nm), with absorption coefficients of 96 and 55 cm^−1^ M^−1^, respectively [37,38]; and a third absorption, corresponding to the intra-charge transfer (ICT) π–π* transition of the cyclopentadienyl rings, which was observed in the deep UV range (UV-C range, electromagnetic radiation with wavelengths below 280 nm) at 272 nm. However, the absorptions and electronic transitions of the 2-substituted-acrylonitrile-substituted ferrocenes differed from those of ferrocene. As can be seen from Table 2, the three main absorptions for the 2-substituted-3-ferrocene acrylonitrile derivatives **18–33** ranged from 214 to 247 nm, which corresponds to the Cp π–π* transition bands; 301 to 340 nm, which corresponds to the Fe (a_1g_) → Cp (e_2g_) CT n–π* transitions; and 426 to 509 nm, which corresponds to Fe (a_1g_) → Fe (e_1g_) or d–d* transitions. The intensity and the energy of the d-d* transitions were strongly affected by the addition of the acrylonitrile group and, to a lesser extent, by the *ortho*, *meta*, and *para* groups of the 2-substituent (aryl, aroyl, and sulfone substituents). The electron-withdrawing effect of the acrylonitrile moiety attached to one of the cyclopentadienyl rings resulted in a significant shift to longer wavelengths. In the spectrum of compound **18** with an unsubstituted benzene ring at the 2-position of the acrylonitrile group, the spectral band (492 nm) corresponding to d–d* transitions shifted by 53 nm to longer wavelengths (red shift) compared to the band in the ferrocene spectrum (440 nm). A similar effect was observed for compound **19** with a 2-pyridyl substituent (d–d* transition at 948 nm). For compounds **20**–**25** with electron-donating substituents at the 2-aryl substitution, a significant shift of the d-d* transitions into longer wavelengths was observed. In the case of compounds **27** and **29** with strong electron-withdrawing substituents, a substantial shift of the d–d* transition bands to longer wavelengths (>500 nm) was observed. However, compounds **26** and **28** with *m*-NO_2_ and *p*-F-phenyl substituents showed similar transition energies (Cp π–π*, MLCT, and d–d* transitions) compared to that of compound **19**. On the other hand, compounds **30**–**32** with α-cyano-aryl chalcone moieties showed a spectral band corresponding to d–d* transitions at wavelengths of 426–442 nm. This shift to lower wavelengths was attributed to the electron-donating ability of the *p*-MeO-phenyl, furyl, and thiophene rings. Additionally, the spectral band corresponding to the MLCT intra-transitions (n–π*) of compounds **30**–**32** shifted (≥11 nm) to longer wavelengths compared to the band in the ferrocene spectrum (325 nm). Compound **33,** a 2-sulfone substituent derivative, showed spectral band absorptions similar to those of compounds **18**–**29** because of the electron-withdrawing effect of the sulfone group that is directly attached to the acrylonitrile moiety. Additionally, the UV/Vis spectra for ferrocenecarboxaldehyde **1** were obtained, and the spectral band corresponding to d–d* transitions of the Fe atom were found to be shifted to a lower wavelength (460 nm) compared to the band in the spectra of the ferrocene–acrylonitrile derivatives. Another spectral band corresponding to the MLCT (n–π*) intra-transition was observed for compounds with C=O, -OCH_3_, and -OH groups. These n-π* intra-transitions correspond to the unshared electrons of the oxygen atoms in these compounds. All the compounds showed an intense spectral band corresponding to π–π* transitions (UV-C range) in the cyclopentadienyl-acrylonitrile system.

**Table 2:** UV/Vis absorption data of 2-substituted-3-ferrocene acrylonitrile derivatives **18–33** and ferrocenecarboxaldehyde **1**.

Comp.	π–π* Transition ^1^		n–π* Transition ^2^		d–d* Transition ^3^	
	λ (nm)	ε (×10^4^ cm^−1^ M^−1^)	λ (nm)	ε (×10^4^ cm^−1^ M^−1^)	λ (nm)	ε (×10^3^ cm^−1^ M^−1^)
(*Z*)-**18**	214	3.49	326	4.90	492	3.05
(*Z*)-**19**	211	3.64	329	2.61	489	2.93
(*Z*)-**20**	214	4.39	336	3.29	486	3.83
(*Z*)-**21**	213	5.00	338	2.28	492	3.08
(*Z*)-**22**	213	8.30	301	3.11	485	6.25
(*Z*)-**23**	212	3.02	335	2.05	498	2.99
(*Z*)-**24**	247	2.06	360	1.44	480	2.41
(*Z*)-**25**	215	4.80	329	3.26	476	5.23
(*Z*)-**26**	215	7.38	332	1.75	483	2.85
(Z)-**27**	212	2.36	325	2.19	503	2.98
(*Z*)-**28**	213	2.56	325	1.97	491	2.47
(*Z*)-**29**	211	2.95	338	2.09	509	2.40
(*E*)-**30**	213	2.19	337	0.44	426	4.99
(*E*)-**31**	215	1.62	340	0.41	442	2.55
(*E*)-**32**	213	1.80	336	0.51	442	4.77
(*E*)-**33**	215	1.97	338	1.77	490	3.54
**1**	227	4.51	304	2.53	460	2.18

^1^ Cp-system transition. ^2^ Fe-to-Cp charge transfer transition. ^3^ Symmetry-forbidden Fe-Fe transition. λ is the wavelength for each absorption band, the sample path length (*l*) is 1 cm, ε is the molar absorptivity (cm^−1^ M^−1^), and the concentration of the samples is 10^−^⁵ M in acetonitrile.

#### 2.1.3. Electrochemical Properties

**Figure 5:**
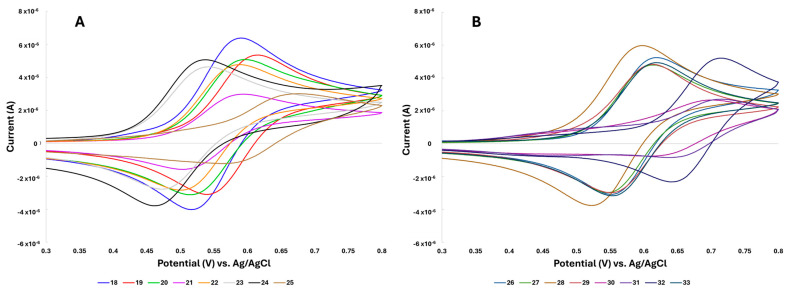
Cyclic voltammograms of ferrocene–acrylonitrile derivatives **18–25** (**A**) and **26–33** (**B**) in 0.1 M nBu_4_PF_6_/acetonitrile. The working electrode was graphite, the reference electrode was Ag/AgCl, the platinum wire served as the auxiliary electrode, and the scan rate was 50 mV/s.

The electrochemistry of ferrocene–acrylonitrile derivatives **18–33** was investigated using cyclic voltammetry in an acetonitrile solution with 0.1 M *n*Bu_4_NPF_6_ as the electrolyte at a scan rate of 50 mV/s. The cyclic voltammograms of the compounds are shown in Figure 5, and the electrochemical data are compiled in Table 3. The redox potential (*E*_½_) values for ferrocene–acrylonitriles were determined to be between 499 and 679 mV (Table 3), which was greater than for free ferrocene under the same conditions (Fc/Fc^+^, *E*_½_ = 450 mV vs. Ag/AgCl). The redox couple potential refers to the iron component of the ferrocenyl moiety. Moreover, the peak-to-peak potential separation (Δ*E*_p_) values were determined to be between 54 and 109 mV, representing a reversible redox process. The redox couple potential of compounds **18**–**33** varied significantly compared to free ferrocene because of the strong electron-withdrawing effect of the acrylonitrile moiety (α, β-unsaturated nitrile). The ethylene group of the acrylonitrile moiety is between the nitrile and the ferrocenyl groups. Thus, the withdrawal of the nitrile affected the ferrocenyl moiety through the ethylene moiety, thereby increasing the redox potential by ≥49 mV compared to free ferrocene. Furthermore, the substituents at the 2-position of the acrylonitrile moiety also affected the redox potential. The phenyl group in position 2 of compounds **18**–**29** is a slightly electron-withdrawing substituent that also affects the redox potential. In compounds **18** and **19**, the change in redox potential from substituting the 2-phenyl moiety with the more electron-withdrawing 2-(3-pyridyl) moiety was only 24 mV. Moreover, the substituents in the phenyl group also contributed to the change in redox potential. It can be seen that the redox potential for compounds **20**–**24** (499–556 mV) decreased by 22–79 mV compared to the redox potential of **19** (578 mV). The lower redox potential observed for compound **24** (499 mV) can be attributed to the stronger electron-donating effect of the amine group through resonance compared to the methyl and methoxy groups. On the other hand, the redox potential of compound **25** (619 mV) increased by 65 and 41 mV compared to compounds **18** (554 mV) and **19**, respectively. When the 2-phenyl moiety was substituted with strong electron-withdrawing groups, as seen in compounds **26**–**29**, the oxidation potential increased slightly by 6–31 mV compared to compound **18,** and their redox potential values were comparable to those of compound **19**. In compounds **30**–**32** with greater redox potential values of 655–678 mV, the ethylene group is between the ferrocenyl and the 2-carbonyl groups. Therefore, the electron-withdrawal effect of the carbonyl group affected the oxidation of the ferrocenyl group through the ethylene group, thereby increasing the redox potential values by 101–124 mV. In terms of substituents for the acrylonitrile moiety at the 2-position, the carbonyl group was considered to have a stronger electron-withdrawing effect compared to the phenyl moiety. This effect can also be compared to compound **33**, a 2-sulfonyl derivative with a redox potential value (586 mV) that was lower than that of compounds **30**–**32** by 69–92 mV. It is well known that sulfonyl groups are more electron-attracting than carbonyl ones [39]. However, the carbonyl group is much more efficient in charge delocalization than the sulfonyl group. This is due to the higher efficiency of p-p overlap between the carbonyl and ethylene groups than the delocalization occurring in the sulfonyl one.

**Table 3:** Cyclic voltammetry values for ferrocene–acrylonitrile derivatives **18–33** in 0.1 M nBu_4_PF_6_/acetonitrile obtained from voltammograms (versus Ag/AgCl).

Comp.	*E*_½_ (mV) ^a^	Δ*E_p_* (mV) ^b^
(*Z*)-**18**	554	73
(*Z*)-**19**	578	76
(*Z*)-**20**	556	77
(*Z*)-**21**	548	88
(*Z*)-**22**	544	86
(*Z*)-**23**	506	67
(*Z*)-**24**	499	77
(*Z*)-**25**	619	115
(*Z*)-**26**	585	64
(*Z*)-**27**	585	73
(*Z*)-**28**	560	75
(*Z*)-**29**	584	63
(*E*)-**30**	655	87
(*E*)-**31**	678	75
(*E*)-**32**	676	72
(*E*)-**33**	586	61

All compounds were tested in an acetonitrile solution with 0.1 M nBu_4_PF_6_ (supporting electrolyte) at 25 °C and a scan rate of 50 mV/s. ^a^ E_½_ = (E_pc_ + E_pa_)/2. ^b^ ΔE_p_ = E_pc_ − E_pa._

Due to their increased metabolic activity, cancer cells produce more hydrogen peroxide (H_2_O_2_) than normal healthy cells [40,41]. As a result, cancer cells function at higher levels of oxidative stress, making them more susceptible to apoptosis from oxidative damage [42]. Therefore, there may be a correlation between the antioxidant effects of ferrocene–acrylonitrile derivatives and their anticancer activity. As mentioned before, ferrocene hybrids and ferrocenium salts can generate reactive oxygen species (ROS), specifically hydroxy radicals (HO^−^), through reactions with endogenous H_2_O_2_. The observed experimental redox potentials of the ferrocene–acrylonitrile derivatives (499–678 mV) are below the oxidation potential of H_2_O_2_ (880–1760 mV) [43], suggesting that intracellular oxidation of ferrocene–acrylonitriles by H_2_O_2_ is favorable via the Fenton-like [44] reaction. Therefore, this would correlate to an in vitro biological application of ferrocene–acrylonitrile derivatives **18**–**33**.

### 2.2. Biological Activity

#### 2.2.1. In Vitro Anticancer Activity

**Figure 6:**
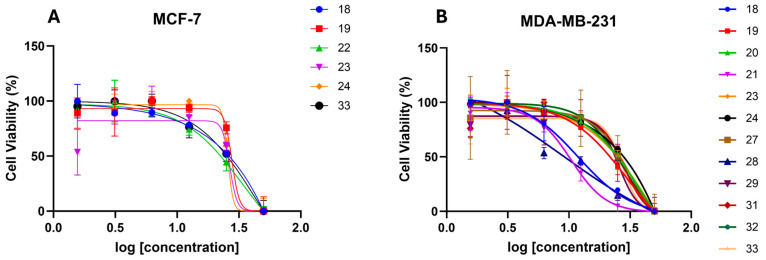
Log-dose response curve for ferrocene–acrylonitrile derivatives (*Z*)-**18–29** and (*E*)-**30–33** against MCF-7 (**A**) and MDA-MB-231 (**B**) breast cancer cell lines. Each data point represents the mean of three replicates, and the error bars represent the standard deviation (SD). Each GI_50_ value was calculated based on the sigmoidal curve that was fitted to the respective data.

Table 4 shows the in vitro antiproliferative activity of ferrocene–acrylonitrile derivatives **18**–**33**. For the most active compounds, log-dose response curves were established (Figure 6). In the MCF-7 (ER+) cancer cell line, compounds **18**–**19**, **22**–**24**, and **33** showed moderate antiproliferative activity with a GI_50_ in the range of 25.8 to 44 µM. In the MDA-MB-231 cell line, all the compounds inhibited cell proliferation, with GI_50_ values ranging from 9 to 41 µM, except for compounds **20**, **22**, **25**, and **30**, which had GI_50_ values above 50 µM. Compound **18**, with a 2-phenyl substituent, exhibited moderate anticancer activity against the MDA-MB-231 and MCF-7 cancer cell lines, with GI_50_ values of 11.9 and 32.8 µM, respectively. The activity of compound **19** with a 2-pyridyl substituent was shown to be moderate, with GI_50_ values of 27.7 and 29.6 µM against the two cancer cell lines, suggesting similar antiproliferative potency. Given these results, further evaluation was performed with substituents for the phenyl group to explore the derivatives’ antiproliferative effect against both cancer cell lines. Among the compounds bearing strong electron-donating substituents at the 2-phenyl group, two (**23** and **24**) demonstrated moderate activity against both cancer cell lines with GI_50_ values of 25.8 to 33.3 µM. However, compounds **20** and **25**, with *para*-methoxy- and *para*-hydroxy substituents, were ineffective against both cancer cell lines. On the other hand, four compounds (**26**–**28** and **29**) with electron-withdrawing substituents showed in vitro antiproliferative activity against the MDA-MB-231 cell line with GI_50_ values ranging from 9.1 to 41.4 µM. Notably, compound **28**, with a *para*-fluoro substituent, was the most promising and exhibited very good anticancer activity against the MDA-MB-231 cell line with a GI_50_ of 9.1 µM. However, compounds **26**–**29** were ineffective against the MCF-7 cell line. The *E*-isomers of compounds **26** and **28** were separable and their antiproliferative effects against both cancer cell lines were also evaluated to compare the potency of both isomers. Interestingly, the (*E*)-**26** and (*E*)-**28** compounds were ineffective against both cancer cell lines. On the other hand, replacing the ferrocenyl moiety with a phenyl group (compare **35** with (*Z*)-**28**) was associated with a loss of activity against both cell lines, with GI_50_ values above 50 µM. Two of the α-cyano aryl chalcone derivatives (**31** and **32**) exhibited antiproliferative activity against the MDA-MB-231 cancer cell line with GI_50_ values of 25.7 and 28.4 µM. Compound **30** was ineffective (GI_50_ > 50 µM) against both cell lines. Compound **33**, an (*E*)-3-ferrocenyl-2-(phenylsulfonyl)acrylonitrile derivative, exhibited higher potency against the MDA-MB-231 cell line with a GI_50_ of 27 µM compared to MCF-7, which showed a weak potency with a GI_50_ of 44 µM. From the results of the cytotoxicity assay against the MCF-7 cancer cell line and metastatic cell line MDA-MB-231, it was found that compounds **18** and **28** had the strongest effects (GI_50_ of 11.9 and 9.1 µM, respectively) against the MDA-MB-231 cell line compared to the MCF-7 cell line, with GI_50_ values of 32.8 to >50 μM.

**Table 4:** In vitro antiproliferative activity of ferrocene–acrylonitrile derivatives against MCF-7 and MDA-MB-231 cell lines.

Comp.	GI_50_ (µM) ^a^	
	MCF-7	MDA-MB-231
(*Z*)-**18**	32.8	11.9
(*Z*)-**19**	27.7	29.6
(*Z*)-**20**	>50	>50
(*Z*)-**21**	>50	38.8
(*Z*)-**22**	33.9	>50
(*Z*)-**23**	26.6	32.6
(*Z*)-**24**	25.8	33.3
(*Z*)-**25**	>50	>50
(*Z*)-**26**	>50	41.4
(*E*)-**26**	>50	>50
(*Z*)-**27**	>50	31.4
(*Z*)-**28**	>50	9.1
(*E*)-**28**	>50	>50
(*Z*)-**29**	>50	25.4
(*E*)-**30**	>50	>50
(*E*)-**31**	>50	28.4
(*E*)-**32**	>50	27.3
(*E*)-**33**	44	27.3
(*Z*)-**35**	>50	>50

^a^ GI_50_ = compound concentration required to inhibit MCF-7 or MDA-MB-231 proliferation by 50% after 48 h of treatment. Values are expressed as the means of triplicate experiments, and the standard deviation (SD) was <10%.

#### 2.2.2. In Vitro Anti-Migration Activity

To assess the anti-migration activity of ferrocene–acrylonitrile derivatives **18**–**33** in vitro, we examined their effects on the migration of the metastatic breast cancer cell line MDA-MB-231 using the wound-healing assay (scratch method) at concentrations that do not affect cell viability. We chose MDA-MB-231 breast cancer cells over MCF-7 due to their enhanced metastatic and migratory properties, due to concomitant Rac and Cdc42 expression, compared to the non-metastatic MCF-7 cells with poor migration abilities. Moreover, epithelial-to-mesenchymal transition (EMT) and its associated transcription factors downregulate genes that cause the reorganization of the cell cytoskeleton, thereby increasing the invasiveness and metastasis of cancer cells [45]. Therefore, EMT correlates with the migration and invasiveness abilities of cancer cells. Through the Fenton reaction, ferrocene and ferrocene-based hybrids generate reactive oxygen species (ROS), leading to cell death via ferroptotic mechanisms. There is evidence that cancer cells undergoing EMT are vulnerable to ferroptotic cell death [12]. Since the MDA-MB-231 cell line is an EMT-positive cell line, we hypothesized that the ferrocene–acrylonitrile derivatives would reduce cell migration. Therefore, the effect of compounds **18**–**33** on the migration of MDA-MB-231 metastatic cell lines was examined. The relative migration of the cells treated with the ferrocene–acrylonitrile derivatives compared with the control (untreated MDA-MB-231 cells) is summarized in Table 5.

**Table 5:** In vitro anti-migration activity of ferrocene–acrylonitrile derivatives against MDA-MB-231 cell line.

Comp.	Migration (%) ^a,b^	Concentration (µM) ^c^
(*Z*)-**18**	98.14 ± 4.31	2.4
(*Z*)-**19**	97.99 ± 2.82	5.9
(*Z*)-**20**	96.98 ± 3.54	10
(*Z*)-**21**	96.91 ± 3.74	7.8
(*Z*)-**22**	99.47 ± 1.32	10
(*Z*)-**23**	98.32 ± 3.17	6.5
(*Z*)-**24**	98.46 ± 4.17	6.7
(*Z*)-**25**	87.46 ± 4.11	10
(*Z*)-**26**	99.18 ± 0.47	10
(*E*)-**26**	99.04 ± 2.16	10
(*Z*)-**27**	99.37 ± 0.53	6.3
(*Z*)-**28**	97.35 ± 4.65	1.8
(*E*)-**28**	98.79 ± 0.03	10
(*Z*)-**29**	99.38 ± 1.10	5.1
(*E*)-**30**	99.02 ± 0.03	5.4
(*E*)-**31**	98.85 ± 0.03	5.1
(*E*)-**32**	99.28 ± 0.03	5.4
(*E*)-**33**	98.98 ± 2.78	10
(*Z*)-**35**	99.10 ± 0.03	10

^a^ After 24 h, MDA-MB-231 cell migration was determined by measuring the distance traveled from the edge of the scratch toward the center of the scratch relative to the control. ^b^ Results are presented as means ±SD of three independent experiments. ^c^ Percent relative migration values at 10 µM or at concentrations that do not affect cell viability.

**Figure 7:**
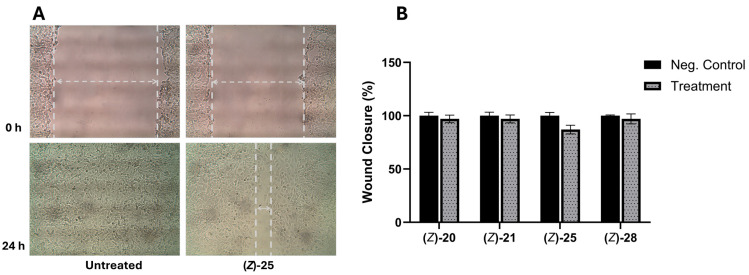
Inhibitory effect of compounds (*Z*)-**20**, (*Z*)-**21**, (*Z*)-**25**, and (*Z*)-**28** on MDA-MB-231 cell migration in wound-healing assay. MDA-MB-231 cells were treated with vehicle or with compounds (*Z*)-**20**, (*Z*)-**21**, (*Z*)-**25**, and (*Z*)-**28**. (**A**) The representative photomicrographs were obtained at 0 and 24 h. The percent relative migration values are the average of three independent experiments. The dotted lines show the area occupied by the initial scraping at 0 h and the wound edge at 24 h. (**B**) The percent migration at 10 μM or at concentrations that do not affect cell viability.

Among the nineteen compounds tested for their anti-migratory effect on the highly metastatic MDA-MB-231 cell line, four compounds (**20**, **21**, **25**, and **28**) inhibited migration by 3–13%. The *Z*-isomer of compound **28** exhibited the most cytotoxic effect against MDA-MB-231 cells, which only showed 3% anti-migratory activity at a concentration of 1.8 µM. However, compound **25** was ineffective against both cancer cell lines, with the highest anti-migratory activity (13% inhibition) at 10 µM (Figure 7). The other three compounds (**22**, **23**, and **24**), which have strong donating-electron substituents, showed low anti-migratory activity. Compound **18** exhibited good antiproliferative activity against the MDA-MB-231 cell line but low anti-migratory activity, achieving 2% inhibition at a concentration of 2.4 µM. On the other hand, the compounds with strong electron-withdrawing substituents (**26**–**29**), which showed good to moderate antiproliferative activity against both cancer cell lines, did not exhibit significant anti-migratory activity. When comparing the anti-migratory activity of Z- and *E*-isomers, specifically for compounds **26** and **28**, the (*E*)-**26** and (*E*)-**28** compounds did not show anti-migratory activity, similar to (Z)-**26** and (Z)-**28** (1 and 3% anti-migration, respectively). The α-cyano aryl chalcone derivatives **30**–**32**, with moderate GI_50_ values ranging from 25.7 to 28.4 µM against both cancer cell lines, did not inhibit migration. The (*E*)-3-ferrocenyl-2-(phenylsulfonyl)acrylonitrile derivative **33,** with a GI_50_ value of 27.3 µM against MDA-MB-231 cells, did not inhibit migration. Finally, replacing the ferrocene with a phenyl group((*Z*)-**35**) resulted in only 1% migration inhibition, similar to that of compound (*Z*)-**28** (3% migration inhibition).

The absence of any antiproliferative or anti-migratory activity for the (*E*)-**26** and (*E*)-**28** derivatives and compound **35**, together with the fact that several (*Z*)-ferrocene-acrylonitrile derivatives showed promising activity, a structure–activity relationship (SAR) can be suggested. For example, it seems that the presence of a ferrocenyl group at the 3-position of the acrylonitrile moiety and a substituted phenyl ring at the 2-position *trans* to the ferrocenyl group improved both antiproliferative and anti-migratory activities.

## 3. Materials and Methods

### 3.1. General Methods

All experiments were carried out in pre-dried glassware (≥2 h, 80–90 °C) under a nitrogen atmosphere. The nuclear magnetic resonance (NMR) spectra were obtained using a Bruker Ascend 500 MHz spectrometer (Bruker TopSpin version 4.5.0, Bruker Inc., Billerica, MA, USA). The ^1^H (500 MHz) and ^13^C{^1^H} (125 MHz) NMR spectral data were recorded in CDCl_3_ or DMSO-*d*_6_ unless indicated otherwise, and the chemical shift was expressed in parts per million (ppm) relative to CDCl_3_ (δ 7.26 for ^1^H and δ 77.0 for ^13^C) or DMSO-*d*_6_ (δ 2.50 for ^1^H and δ 39.5 for ^13^C) as the internal standard. The ^1^H NMR data are reported as position (δ), relative integral, multiplicity (s, singlet; d, doublet; t, triplet; q, quartet; dt, doublet of triplets; dd, doublet of doublets; dq, doublet of quartets; m, multiplet; br, broad peak), and coupling constant (*J*) in hertz (Hz). The shift in ppm for multiplets corresponds to the centermost value of the entire splitting pattern. The ^13^C{^1^H} NMR data are reported as position (δ). The NMR data analysis was performed using JEOL JASON software version 4.0. The UV/Vis spectra were recorded with a UV5 Nano Mettler Toledo (LabX UV/Vis software, Model UV/Vis Excellence 30254729, Greifensee, Switzerland) spectrophotometer with a wavelength range of 190–1100 nm at a concentration of 10^−5^ M in acetonitrile using a quartz cell (1 cm path length) at ambient temperature, and acetonitrile was used as the blank. Infrared spectra were recorded using a Nicolet Summit LITE FTIR Spectrometer (OMNIC Paradigm software version 2.8, Thermo Scientific, Saint Louis, MO, USA) with a lithium tantalate (LiTaO_3_) detector, a spectral range of 8000–350 cm^−1^, an optimized and mid-infrared KBr beamsplitter, and a spectral resolution of >0.6 cm^−1^. The electron-ionization mass spectra (EI-MS) data were obtained using an Agilent (Santa Clara, CA, USA) 6890 N GC System (Software version N.05.04) with a 5973 Network Mass Selective Detector utilizing a column with a 0.33 μm thick film of fused silica (length of 50 m length, 0.2 mm diameter, low polarity, Ultra 1 phase) and a temperature range of −60 °C to 325/350 °C. The method for running samples was as follows: (a) inlet temperature of 250 °C, pressure of 32.8 psi, and total flow of 23.5 mL/min; (b) constant flow in column, a pressure of 32.8 psi, flow of 1.0 mL/min, and average velocity of 29 cm/s; and (c) ramp rate of 20 °C/min with an initial temperature of 70 °C for 2 min and final temperature of 280 °C for 25 min, with a total runtime of 37.5 min.

### 3.2. Synthesis Methods

The progress of the reaction was monitored via thin-layer chromatography (TLC) analysis using general-purpose silica gel on 5 × 20 cm glass plates (250 μm thick, 60 Å pore diameter) with a UV indicator, which was visualized with UV fluorescent Spectroline E Series Ultraviolet lamps (254 nm), followed by, in most cases, staining with iodine (I_2_). The retention factor (*R_f_*) was calculated by measuring the distance traveled by the compound divided by the distance traveled by the solvent (a mixture of hexane/ethyl acetate at a ratio of 3:1 or 1:1). The compounds were purified via column chromatography over silica gel (70–230 mesh, 60 Å pore diameter) with the appropriate size column (24/40, 12 in. × 0.5 in. or 12 in. × 0.72 in.) or via flash chromatography using a Combi*Flash* EZ Prep Chromatography System (PeakTrack software version 3.1) with integrated ELSD, a 200–800 nm UV-Vis variable wavelength detector, and a 12 g pre-packed silica gel column (high-performance RediSep Gold^®^; Teledyne, Thousand Oaks, CA, USA) with a particle size of 20–40 μm. All acetonitrile derivatives (**2–17**) were obtained from MilliporeSigma (Burlington, MA, USA). The sodium methoxide reagent was obtained from MilliporeSigma as a 25 wt.% solution in methanol.

#### General Procedure for the Synthesis of 2-Substituted-3-Ferrocene-Acrylonitrile Derivatives **18**–**33**

A 50 mL three-neck round-bottom flask equipped with a reflux condenser was charged with the appropriate substituted acetonitrile (**2**–**17**) (0.7 mmol) and 5% NaOMe (0.343 mL, 1.5 mmol) in methanol (1.7 mL). After 15 min of stirring at room temperature, ferrocenecarboxaldehyde **1** (0.1070 g, 0.5 mmol) was added to the reaction mixture, and the mixture was refluxed for 5 h. After the reaction was completed (and analyzed by TLC), the mixture was allowed to reach room temperature. The mixture was diluted with water (15 mL), and the product was extracted using ethyl acetate (3 × 10 mL). The organic layer was washed with brine, dried with Na_2_SO_4_, filtered, and concentrated under low pressure. The crude oil was purified via flash column chromatography over silica gel and isocratically eluted using 30% or 50% ethyl acetate (EtOAc) in hexane (or a stepped gradient of 20% to 100% EtOAc within one column volume if purified using the *EZ* Prep Combi*Flash*), and the product was obtained as a solid.

*(Z)-3-Ferrocenyl-2-phenylacrylonitrile* (**18**)

The product was obtained as a deep red-purple solid (0.1535 g, 0.49 mmol, 98%), lit. (isolated yield was not reported) [30]. TLC analysis in hexane/ethyl acetate (3:1) *R_f_* = 0.80 (UV, I_2_). ^1^H NMR (500 MHz, CDCl_3_) δ 7.61 (d, *J* = 7.75 Hz, 2H), 7.42 (t, *J* = 9.0 Hz, 2H), 7.39 (s, 1H), 7.35 (t, *J* = 8.0 Hz, 1H), 4.97 (s, 2H), 4.54 (s, 2H), 4.24 (s, 5H). ^13^C{^1^H} NMR (125 MHz, CDCl_3_) δ 143.2, 134.7, 129.0, 128.2, 125.1, 119.1 (C≡N nitrile), 106.7, 77.4, 71.5, 70.1, 69.8 (C_5_H_5_ Cp). LRGC-MS *m*/*z* 313 [M]^+^, 299 [M − N]^+^, 245 [M − C_5_H_5_]^+^. FT-IR (neat) 2208 (C≡N), 2855, 2921 (=C-H), 3026, 3092 (=C-H) cm^−1^.

*(Z)-3-Ferrocenyl-2-(3-pyridyl)acrylonitrile* (**19**)

The product was obtained as a red solid (0.1207 g, 0.38 mmol, 77%), lit. (isolated yield was not reported) [30]. TLC analysis in hexane/ethyl acetate (3:1) *R_f_* = 0.26 (UV, I_2_). ^1^H NMR (500 MHz, CDCl_3_) δ 8.85 (s, 1H), 8.58 (d, *J* = 4.67 Hz, 1H), 7.87 (d, *J* = 8.13 Hz, 1H), 7.44 (s, 1H), 7.34 (dd, *J* = 4.43, 7.33 Hz, 1H), 4.99 (s, 2H), 4.58 (s, 2H), 4.23 (s, 5H). ^13^C{^1^H} NMR (125 MHz, CDCl_3_) δ 149.0, 146.2, 145.2, 132.5, 130.8, 123.6, 118.3 (C≡N nitrile), 103.1, 76.8, 72.1, 70.3, 70.0 (C_5_H_5_ Cp). LRGC-MS *m*/*z* 314 [M]^+^, 247 [M − C_5_H_5_]^+^, 222 [M − C_6_H_6_N]^+^, 192 (M − C_5_H_6_Fe]^+^. FT-IR (neat) 2210 (C≡N), 2800–2924 (=C-H) and 3000–3089 (=C-H) cm^−1^.

*(Z)-3-Ferrocenyl-2-(4-methoxyphenyl)acrylonitrile* (**20**)

The product was obtained as a red solid (0.101 g, 0.29 mmol, 59%), lit. isolated yield (18 mg, 6%) [30]. TLC analysis in hexane/ethyl acetate (3:1) *R_f_* = 0.74 (UV, I_2_). ^1^H NMR (500 MHz, CDCl_3_) δ 7.54 (d, *J* = 8.6 Hz, 2H), 7.26 (s, 1H), 6.95 (d, *J* = 8.6 Hz, 2H), 4.95 (s, 2H), 4.51 (s, 2H), 4.23 (s, 5H), 3.84 (s, 3H). ^13^C{^1^H} NMR (125 MHz, CDCl_3_) δ 159.6, 141.0, 127.2, 126.3, 119.2 (C≡N nitrile), 114.3, 106.3, 77.7, 71.1, 69.8, 69.7 (C_5_H_5_ Cp), 55.3 (OCH_3_ methoxy). LRGC-MS *m*/*z* 343 [M]^+^, 278 [M − C_5_H_5_]^+^, 251 [M − C_6_H_6_N]^+^, 195 [M − C_5_H_6_Fe]^+^. FT-IR (neat) 1288, 1335 (C-O), 2207 (C≡N), 2836–2922 (=C-H), 3000–3100 (=C-H) cm^−1^.

*(Z)-3-Ferrocenyl-2-(3,4,5-trimethoxyphenyl)acrylonitrile* (**21**)

The product was obtained as a deep red solid (0.1775 g, 0.44 mmol, 88.0%). TLC analysis in hexane/ethyl acetate (3:1) *R_f_* = 0.51 (UV, I_2_). ^1^H NMR (500 MHz, CDCl_3_) δ 7.28 (s, 1H), 6.79 (s, 2H), 4.97 (s, 2H), 4.54 (s, 2H), 4.24 (s, 5H), 3.94 (s, 6H), 3.89 (s, 3H). ^13^C{^1^H} NMR (125 MHz, CDCl_3_) δ 153.3, 142.4, 138.1, 130.1, 118.8 (C≡N nitrile), 106.3, 102.3, 71.2, 69.8, 69.5 (C_5_H_5_ Cp), 60.6 (OCH_3_ methoxy), 56.1 (OCH_3_ 2 methoxy). LRGC-MS *m*/*z* 403 [M]^+^, 251 [M − C_6_H_8_OFe]^+^, 220 [M − C_8_H_11_O_2_Fe]^+^. FT-IR (neat) 1243, 1335 (C-O), 2209 (C≡N), 2838, 2921 (=C-H), 3092 (=C-H) cm^−1^.

*(Z)-3-Ferrocenyl-2-(3-methoxyphenyl)acrylonitrile* (**22**)

The product was obtained as a deep red solid (0.1046 g, 0.31 mmol, 61%). TLC analysis in hexane/ethyl acetate (3:1) *R_f_* = 0.84 (UV, I_2_). ^1^H NMR (500 MHz, CDCl_3_) δ 7.39 (s, 1H), 7.33 (t, *J* = 6.5 Hz, 1H), 7.20 (d, *J* = 7.9 H, 1H), 7.13 (s, 1H), 6.90 (d, *J* = 8.1 Hz, 1H), 4.97 (s, 2H), 4.54 (s, 2H), 4.24 (s, 5H), 3.87 (s, 3H). ^13^C{^1^H} NMR (125 MHz, CDCl_3_) δ 160.0, 143.5, 136.1, 130.0, 119.0 (C≡N nitrile), 117.5, 113.6, 110.9, 106.4, 71.5, 70.1, 69.8 (C_5_H_5_ Cp), 55.3 (OCH_3_ methoxy). FT-IR (neat) 1288, 1216 (C-O), 2209 (C≡N), 2920–2835 (=C-H), 3000–3100 (=C-H) cm^−1^.

*(Z)-3-Ferrocene-2-(4-methylphenyl)acrylonitrile* (**23**)

The product was obtained as a deep red-purple solid (0.1456 g, 0.45 mmol, 89%). TLC analysis in hexane/ethyl acetate (3:1) *R_f_* = 0.83 (UV, I_2_). ^1^H NMR (500 MHz, CDCl_3_) δ 7.49 (d, *J* = 8.0 Hz, 2H), 7.33 (s, 1H), 7.22 (d, *J* = 7.9 Hz, 2H), 4.96 (t, *J* = 1.7 Hz, 2H), 4.51 (t, *J* = 1.7 Hz, 2H), 4.23 (s, 5H), 2.37 (s, 3H, -OCH_3_). ^13^C{^1^H} NMR (125 MHz, CDCl_3_) δ 142.1, 138.2, 131.9, 129.7, 125.0, 119.2 (C≡N nitrile), 106.7, 77.6, 71.4, 70.0, 69.8 (C_5_H_5_ Cp), 21.2 (CH_3_ methyl). LRGC-MS *m*/*z* 327 [M]^+^, 262 [M − C_5_H_5_]^+^, 235 [M − C_6_H_6_N]^+^, 179 [M − C_6_H_6_NFe]^+^. FT-IR (neat) 2207 (C≡N), 2852, 2924 (=C-H), 3024 (−C-H), 3085 (=C-H) cm^−1^.

*(Z)-3-Ferrocenyl-2-(4-aminophenyl)acrylonitrile* (**24**)

The product was obtained as a red solid (0.1438 g, 0.44 mmol, 88%). TLC analysis in hexane/ethyl acetate (3:1) *R_f_* = 0.29 (UV, I_2_). ^1^H NMR (500 MHz, CDCl_3_) δ 7.40 (d, *J* = 8.1 Hz, 2H), 7.18 (s, 1H), 6.70 (d, *J* = 8.1 Hz, 2H), 4.92 (s, 2H), 4.47 (s, 2H), 4.22 (s, 5H), 3.83 (bs, 2H, -NH_2_). ^13^C{^1^H} NMR (125 MHz, CDCl_3_) δ 146.7, 139.3, 126.4, 125.0, 119.4 (C≡N nitrile), 115.2, 106.9, 78.1, 70.9, 69.6 (C_5_H_5_ Cp). LRGC-MS *m*/*z* 328 [M]^+^, 263 [M − C_5_H_5_]^+^, 236 [M − C_6_H_6_N]^+^, 180 [M − C_6_H_6_NFe]^+^. FT-IR (neat) 1265 (C-N), 2207 (C≡N), 3032 (=C-H), 3218 (=C-H), 3032 (=C-H), 3455 and 3367 (s) (N-H) cm^−1^.

*(Z)-3-Ferrocenyl-2-(4-hydroxyphenyl)acrylonitrile* (**25**)

The product was obtained as a red solid (0.0793 g, 0.24 mmol, 48%). TLC analysis in hexane/ethyl acetate (3:1) *R_f_* = 0.83 (UV, I_2_). ^1^H NMR (500 MHz, CDCl_3_) δ 7.48 (d, *J* = 8.4 Hz, 2H), 7.24 (s, 1H), 6.88 (d, *J* = 8.2 Hz, 2H), 5.51 (bs, 1H), 4.93 (s, 2H), 4.50 (s, 2H), 4.23 (s, 5H). ^13^C{^1^H} NMR (125 MHz, CDCl_3_) δ 156.0, 141.3, 127.4, 126.7, 119.3 (C≡N nitrile), 115.9, 106.3, 77.7, 71.3, 69.8, 69.7 (C_5_H_5_ Cp). LRGC-MS *m*/*z* 329 [M]^+^, 313 [M-OH]^+^, 248 [M − C_5_H_6_O]^+^, 165 [M − C_6_H_6_NOFe]^+^. FT-IR (neat) 1225 (C-O), 2219 (C≡N), 2920 (=C-H), 3312 (O-H) cm^−1^.

*(Z)-3-Ferrocenyl-2-(3-nitrophenyl)acrylonitrile* (**26**)

The product was obtained as a deep red solid (0.1253 g, 0.35 mmol, 70%). TLC analysis in hexane/ethyl acetate (3:1) *R_f_* = 0.81. ^1^H NMR (500 MHz, CDCl_3_) δ 8.43 (bs, 1H), 8.17 (bs, 1H), 7.92 (bs, 1H), 7.58 (d, *J* = 7.8 Hz, 1H), 7.57 (s, 1H), 5.01 (bs, 2H) 4.79 (bs, 2H), 4.26 (bs, 5H). ^13^C{^1^H} NMR (125 MHz, CDCl_3_) δ 148.8, 146.2, 136.5, 130.9, 130.1, 122.5, 119.3 (C≡N nitrile), 118.3, 103.8, 76.4, 73.1, 72.3, 70.0 (C_5_H_5_ Cp). LRGC-MS *m*/*z* 358 [M]^+^, 312 [M − NO_2_]^+^, 190 [M − C_5_H_6_NO_2_Fe]^+^, 164 [C_6_H_6_N_2_O_2_Fe]^+^. FT-IR (neat) 1524 (st) (N-O), 1342 (st) (N-O), 2213 (C≡N), 2851, 2921 (=C-H), 3088 (st) (=C-H) cm^−1^.

*(E)-3-Ferrocenyl-2-(3-nitrophenyl)acrylonitrile* (**26**)

The crude mixture was purified via flash column chromatography over a silica gel column using a stepped gradient, starting at 20% EtOAc in hexane and increasing to 100% EtOAc within one column volume, and the CombiFlash system. The product was obtained as a red solid (0.0017 g, 0.0048 mmol, 0.96%). ^1^H NMR (500 MHz, CDCl_3_) δ 8.35 (bs, 1H), 8.19 (bs, 1H), 8.05 (bs, 1H), 7.62 (d, *J* = 7.44 Hz, 1H), 7.56 (s, 1H), 4.79 (bs, 2H) 4.62 (bs, 2H), 3.89 (bs, 5H). ^13^C{^1^H} NMR (125 MHz, CDCl_3_) δ 148.6, 146.3, 136.6, 131.0, 130.2, 122.6, 119.5 (C≡N nitrile), 116.5, 104.0, 76.5, 72.4, 70.5, 69.6 (C_5_H_5_ Cp). LRGC-MS *m*/*z* 358 [M]^+^, 312 [M − NO_2_]^+^, 190 [M − C_5_H_6_NO_2_Fe]^+^, 164 [C_6_H_6_N_2_O_2_Fe]^+^.

*(Z)-3-Ferrocenyl-2-[3-(trifluoromethyl)phenyl]acrylonitrile* (**27**)

The product was obtained as a deep red-purple solid (0.1886 g, 0.495 mmol, 99%). TLC analysis in hexane/ethyl acetate (3:1) *R_f_* = 0.92. ^1^H NMR (500 MHz CDCl_3_) δ 7.82 (s, 1H), 7.79 (d, *J* = 8.0 Hz, 1H), 7.61 (d, *J* = 7.6 Hz, 1H), 7.54 (t, *J* = 7.8 Hz, 1H), 7.46 (s, 1H), 7.46 (s, 1H), 5.00 (s, 2H), 4.59 (s, 2H), 4.26 (s, 5H). ^13^C{^1^H} NMR (125 MHz, CDCl_3_) δ 145.1, 135.5, 131.4 (q, CF_3_), 129.5, 128.3, 124.5, 121.4, 118.6 (C≡N nitrile), 115.8, 104.8, 76.7, 72.0, 70.3, 69.9 (C_5_H_5_ Cp). LRGC-MS *m*/*z* 381 [M]^+^, 316 [M − CHNF_2_]^+^, 240 [C_5_H_6_FFe]^+^, 220 [C_5_H_6_F_2_Fe]^+^, 190 [C_6_H_6_F_3_Fe]^+^. FT-IR (neat) 1332 (st) (CF_3_), 2210 (C≡N), 2862, 2922 (=C-H), 3094 (=C-H) cm^−1^.

*(Z)-3-Ferrocenyl-2-(4-fluorophenyl)acrylonitrile* (**28**)

The product was obtained as a red solid (0.1441 g, 0.44 mmol, 87%), lit. isolated yield (281 mg, 91%) [30]. TLC analysis in hexane/ethyl acetate (3:1) *R_f_* = 0.83. ^1^H NMR (500 MHz, CDCl_3_) δ 7.57 (t, *J* = 6.0 Hz, 2H), 7.30 (s, 1H), 7.10 (t, *J* = 8.0 Hz, 2H), 4.95 (s, 2H), 4.54 (s, 2H), 4.24 (s, 5H). ^13^C{^1^H} NMR (125 MHz, CDCl_3_). δ 163.6 (d, *J*_C-F_ = 248.9 Hz, =C-F), 161.6, 143.2, 130.9, 126.9, 119.0 (C≡N nitrile), 116.1, 105.7, 71.6, 70.1, 69.9 (C_5_H_5_ Cp). LRGC-MS Retention Time (min) 11.91, AUC *m*/*z* 331 [M]^+^, 266 [M − C_5_H_5_]^+^, 239 [M − C_6_H_6_N]^+^, 183 [M-FeC_6_H_6_N]^+^. FT-IR (neat) 1234 (=C-F), 2210 (C≡N), 2852, 2926 (=C-H), 3046, 3093 (st) (=C-H) cm^−1^.

*(E)-3-Ferrocenyl-2-(-(4-fluorophenyl)acrylonitrile* (*E*-**28**)

The crude mixture was purified via flash column chromatography over a silica gel column using a stepped gradient, starting at 10% EtOAc in hexane and increasing to 100% EtOAc within one column volume, and the CombiFlash system. The product was obtained as a red solid (0.0026 g, 0.008 mmol, 1.6%). ^1^H NMR (500 MHz, CDCl_3_) δ 7.52 (t, *J* = 6.1 Hz, 2H), 7.46 (s, 1H), 7.05 (t, *J* = 8.2 Hz, 2H), 4.93 (s, 2H), 4.50 (s, 2H), 4.22 (s, 5H). ^13^C{^1^H} NMR (125 MHz, CDCl_3_). δ 163.6 (d, *J*_C-F_ = 249 Hz, =C-F), 161.4, 143.3, 130.9, 127.0, 119.0 (C≡N nitrile), 116.2, 105.7, 71.3, 70.7, 69.8 (C_5_H_5_ Cp). LRGC-MS *m*/*z* 331 [M]^+^, 266 [M − C_5_H_5_]^+^, 239 [M − C_6_H_6_N]^+^, 183 [M − FeC_6_H_6_N]^+^.

*(Z)-3-Ferrocenyl-2-(4-methylbenzoate)acrylonitrile* (**29**)

The product was obtained as a deep red-purple solid (0.1392 g, 0.38 mmol, 75%). TLC analysis in hexane/ethyl acetate (3:1) *R_f_* = 0.73. ^1^H NMR (500 MHz, CDCl_3_) δ 8.07 (d, *J* = 8.4 Hz, 2H), 7.67 (d, *J* = 8.4 Hz, 2H), 7.51 (s, 1H), 5.00 (d, *J* = 2.0 Hz, 2H), 4.59 (t, *J* = 2.0 Hz, 2H), 4.26 (s, 5H), 3.94 (s, 3H). ^13^C{^1^H} NMR (125 MHz, CDCl_3_) δ 170.6 (C=O carbonyl), 148.8, 145.99, 131.15, 125.1, 125.0, 118.7 (C≡N nitrile), 105.3, 76.9, 72.2, 70.5, 70.0 (C_5_H_5_ Cp), 29.7 (OCH_3_ methyl ester). LRGC-MS *m*/*z* 371 [M]^+^, 306 [M − C_5_H_5_]^+^, 220 [M − C_6_H_8_OFe]^+^, 190 [M − C₇H₉O_2_Fe]^+^. FT-IR (neat) 1279 (C-C-O), 1716 (C=O), 2211(C≡N), 2853, 2922 (=C-H) cm^−1^.

*(E)-3-Ferrocenyl-2-(4-methoxybenzoyl)acrylonitrile* (**30**)

The product was obtained as a deep red solid (0.0426 g, 0.12 mmol, 23%). TLC analysis in hexane/ethyl acetate (3:1) *R_f_* = 0.51. ^1^H NMR (500 MHz, CDCl_3_) δ 8.14 (s, 1H), 7.93 (d, *J* = 8.4 Hz, 1H), 6.99 (d, *J* = 8.3 Hz, 2H), 5.10 (s, 2H), 4.77 (s, 2H), 4.30 (s, 5H), 3.89 (s, 3H). ^13^C{^1^H} NMR (125 MHz, CDCl_3_) δ 186.8 (C=O carbonyl), 163.5, 159.4, 131.5, 129.2, 119.0 (C≡N nitrile), 113.8, 104.3, 75.0, 74.3, 72.0, 70.7 (C_5_H_5_ Cp), 55.5 (OCH_3_ methoxy). LRGC-MS *m*/*z* 371 [M]^+^, 306 [M − C_5_H_5_]^+^, 291 [M − C_5_H_6_N]^+^, 263 [M − C₇H₉O]^+^, 235 [M − C_8_H_10_O]^+^. FT-IR (neat) 1598 (st) (C=O), 2209 (C≡N), 2851, 2922 (=C-H) cm^−1^.

*(E)-3-Ferrocenyl-2-(2-furanoyl)acrylonitrile* (**31**)

The product was obtained as an orange solid (0.0943 g, 0.29 mmol, 57%). TLC analysis in hexane/ethyl acetate (3:1) *R_f_* = 0.71. ^1^H NMR (500 MHz, CDCl_3_) δ 8.43 (s, 1H), 7.74 (d, *J* = 3.3 Hz, 1H), 7.73 (bs, 1H), 6.62 (d, *J* = 1.7 Hz, 1H), 5.13 (s, 2H), 4.83 (s, 2H), 4.30 (s, 5H). ^13^C{^1^H} NMR (125 MHz, CDCl_3_) δ 172.8 (C=O carbonyl), 160.2, 151.0, 147.4, 120.4, 118.9 (C≡N nitrile), 112.6, 101.4, 75.0, 74.8, 72.4, 70.8(C_5_H_5_ Cp). LRGC-MS *m*/*z* 331 [M]^+^, 302 [M − NO]^+^, 266 [M − C_5_H_5_]^+^, 210 [M − C_5_H_5_Fe]^+^. FT-IR (neat) 1640 (st) (C=O), 2210 (C≡N), 2852, 2922 (=C-H) cm^−1^.

*(E)-3-Ferrocene-2-(2-thienylketone)acrylonitrile* (**32**)

The product was obtained as a deep red solid (0.1666 g, 0.48 mmol, 96%). TLC analysis in Hexane/Ethyl Acetate (3:1) *R_f_* = 0.86. ^1^H NMR (500 MHz, CDCl_3_) δ 8.36 (s, 1H), 8.29 (d, *J* = 3.9 Hz, 1H), 7.73 (d, *J* = 4.9 Hz, 1H), 7.19 (t, *J* = 4.4 Hz, 1H), 5.12 (s, 2H), 4.82 (s, 2H), 4.30 (s, 5H). ^13^C{^1^H} NMR (125 MHz, CDCl_3_) δ 177.6 (C=O carbonyl), 160.3, 142.8, 134.8, 133.7, 128.5, 119.3 (C≡N nitrile), 102.0, 79.4, 74.7, 73.2, 70.8 (C_5_H_5_ Cp). LRGC-MS *m*/*z* 347 [M]^+^, 282 [M − C_5_H_5_]^+^, 254 [M − C_6_H_6_N]^+^, 227 [M − C_5_H_5_Fe]^+^. FT-IR (neat) 1661 (st) (C=O), 2206 (C≡N), 2849, 2918 (=C-H), 3097 (=C-H) cm^−1^.

*(E)-3-Ferrocenyl-2-(phenylsulfonyl)acrylonitrile* (**33**)

The product was obtained as a deep orange solid (0.1376 g, 0.37 mmol, 73%). TLC analysis in hexane/ethyl acetate (3:1) *R_f_* = 0.89. ^1^H NMR (500 MHz, CDCl_3_) δ 8.17 (s, 1H), 8.0 (d, *J* = 7.9 Hz, 2H), 7.68 (t, *J* = 7.5 Hz, 1H), 7.59 (t, *J* = 7.7 Hz, 2H), 4.96 (s, 2H), 4.77 (s, 2H), 4.24 (s, 5H). ^13^C{^1^H} NMR (125 MHz, CDCl_3_) δ 145.8, 144.7, 130.9, 125.1, 125.0, 118.7 (C≡N nitrile), 105.3, 76.9, 72.2, 70.5, 70.0 (C_5_H_5_ Cp). LRGC-MS *m*/*z* 377 [M]^+^, 312 [M − C_5_H_5_]^+^, 281 [M − C_5_H_5_O_2_]^+^, 235 [M − C_11_H_10_]^+^. FT-IR (neat) 1148 (SO_2_), 1319 (=C-S), 1446 (SO_2_), 2210 (C≡N), 2852, 2923 (=C-H), 3026 (=C-H) cm^−1^.

*Synthesis of (Z)-2-(4-Fluorophenyl)-3-phenylacrylonitrile* (**35**)

A 50 mL three-neck round-bottom flask equipped with a reflux condenser was charged with 4-fluorophenylacetonitrile **12** (0.084 mL, 0.7 mmol) and 5% NaOMe (1.5 mmol) in methanol (2.3 mL). After 15 min of stirring at room temperature, benzaldehyde **34** (0.051 mL, 0.5 mmol) was added to the reaction mixture and refluxed for 1 h. After the reaction was completed (and analyzed by TLC), the mixture was allowed to reach room temperature. The mixture was diluted with water (15 mL), and the product was extracted using ethyl acetate (3 × 10 mL). The organic layer was washed with brine, dried with Na_2_SO_4_, filtered, and concentrated under low pressure. The crude oil was purified via flash column chromatography over a silica gel column with a stepped gradient from 20% EtOAc in hexane to 100% EtOAc within one column volume using CombiFlash, and the product **35** was obtained as a white crystalline solid (0.1004 g, 0.45 mmol, 90%), m.p. 103–105 °C, lit. m.p. 105 °C, (95% yield) [31]. TLC analysis in hexane/ethyl acetate (3:1) *R_f_* = 0.92. ^1^H NMR (500 MHz, CDCl_3_) δ 7.88 (d, *J* = 7.4 Hz, 2H), 7.66 (dd, *J* = 5.1, 8.6 Hz, 2H), 7.49 (dd, *J* = 1.9, 8.6 Hz, 1H), 7.46 (d, *J* = 2.6 Hz, 2H), 7.45 (s, 1H), 7.15 (t, *J* = 8.4 Hz, 2H). ^13^C{^1^H} NMR (125 MHz, CDCl_3_) δ 163.2 (d, *J_FC_* = 250 Hz, =C-F), 142.1 (CH vinyl hydrogen), 133.5, 130.6, 129.2, 129.0, 127.9, 117.8 (C≡N nitrile), 116.2, 116.0, 110.6. LRGC-MS *m*/*z* 223 [M]^+^, 208 [M-HN]^+^, 196 [M − CHN]^+^. FT-IR (neat) 1235 (=C-F), 1508, 1601, 2215 (C≡N), 3053 (=C-H) cm^−1^.

### 3.3. Single-Crystal X-Ray Diffraction Experiments

Compounds (*Z*)-**21**, (*Z*)-**27**, (*Z*)-**29**, and (*E*)-**33** were crystallized by slow evaporation of dichloromethane/hexane (1:1). X-ray-quality single crystals were measured using a Rigaku SuperNova X-ray diffraction instrument equipped with a HyPix3000 X-ray detector and a Cu-K (λ = 1.5417 Å) radiation source (The Woodlands, TX, USA). Crystal data and structure refinement details for H atoms were processed using the following programs: CrysAlis PRO [46], SHELXT [47], SHELXL2018/3 [48], and OLEX2 [49], which were used to geometrically calculate positions and refine them into riding atoms using C-H = 0.93 Å and Uiso(H) = 1.2 Ueq(C).

Full crystallographic data for (*Z*)-**21**, (*Z*)-**27**, (*Z*)-**29**, and (*E*)-**33** have been deposited at the Cambridge Crystallographic Data Centre (CCDC) under the deposition numbers 2455357, 2455367, 2455361, and 2455359, respectively, and are obtainable from The Cambridge Crystallographic Data Centre at www.ccdc.cam.ac.uk/data_request/cif (accessed on 30 May 2025).

### 3.4. Cyclic Voltammetry

The electrochemical measurements of all the compounds were conducted using a BASi Epsilon EClipse™ Potentiostat (Software version 3.0.84, BASi, Mönchengladbach, Germany) in 5 mL of a deoxygenated acetonitrile solution containing 0.1 M of [nBu_4_]PF_6_ as the supporting electrolyte, along with a ferrocene/conjugate concentration of 1.0 × 10^−3^ M at 25 °C. The initial potential was set to 0 mV, with a switch potential of 800 mV and a final potential of 0 mV. The instrument was configured for 5 scans at a rate of 100 mV/s, with a quiet time of 5 s, using three supporting electrodes: graphite as the working electrode, Ag/AgCl as the reference electrode, and Pt as the auxiliary electrode. The potential values for the ferrocene derivatives (versus Ag/AgCl) were obtained from voltammograms. The working electrode was polished with a 0.05 μm alumina slurry for 1–2 min and then rinsed thoroughly with distilled and deionized water. The cleaning process for the graphite electrode was performed between each run.

### 3.5. Biological Evaluation

#### 3.5.1. Cell Culture

The MDA-MB-231 and MCF-7 cells were obtained from the American Type Culture Collection (ATCC) and were cultured in Minimum Essential Medium Eagle (MEME) supplemented with Earle’s Balanced Salt Solution (EBSS), nonessential amino acids (NEAAs), sodium pyruvate, Pen/Strep, L-glutamine, and 10% FBS at 37 °C in 5% CO_2_.

#### 3.5.2. Sulforhodamine B (SRB) Assay

Stock solutions of the compounds (10 mM) were prepared in 100% DMSO. The cells were added to a 75 cm^2^ (2.6 × 10^5^ cells/mL) or 25 cm^2^ flask (1.44 × 105 cells/mL) and grown until 80–90% confluence was reached. The cells were washed with PBS and trypsinized. The concentration of cells was determined using a 1:2 dilution with Trypan Blue and a hemocytometer. After counting the cells, the concentration was adjusted to 7.0–10.0 × 10^4^ cells/mL. Approximately 100 μL of the cell suspension, compounds, positive control, and negative control were added to a 96-well plate in triplicate. The positive control was doxorubicin and the negative control was 0.1% DMSO. All the compounds (at concentrations of 50, 25, 12.5, 6.3, and 1.6 μM) were incubated with the cells at 37 °C for 48 h. Cold 50% TCA was used for fixation and the cells were incubated at 4 °C for 1 hr. The wells were washed and dried before adding 100 μL of 0.4% SRB. Acetic acid was used to remove any excess SRB. For the analysis, a TRIS-BASE Solution (pH = 10.5) was used and the mixture was shaken before being read at 540 nm using an ELISA reader and the software SoftMax Pro 4.8. For each compound, the 50% growth inhibition (GI_50_) concentration was calculated from the sigmoidal dose–response curves (variable slope) generated using the data from the experiments carried out in triplicate. The GI_50_ values were determined using GraphPad Prism V. 6.02 (GraphPad Software, Inc., San Diego, CA, USA).

#### 3.5.3. Wound-Healing Assay (Scratch Method) Using MDA-MB-231 Cancer Cells

Before the assays, cells were grown until they reached 80–90% confluence. For 75 cm^2^ flasks, 10 mL of a 2.6 × 10^5^ cells/mL suspension was used and for 25 cm^2^ flasks, 5 mL of a 1.44 × 10^5^ cells/mL suspension was used. The cells were washed with PBS to remove all traces of FBS. We added trypsin to the cells (2 mL for 25 cm^2^ flasks or 4 mL for 75 cm^2^ flasks), which were then incubated for 5–10 min at 37 °C. At the end of the incubation time, the cells were re-suspended and counted using a hemocytometer and 1:2 dilutions with Trypan Blue. Subsequently, the cell viability was calculated. In a 12-well plate, using a plastic pattern, we drew a pattern with a fine marker on the bottom of the plate. The cells were seeded at a density of 1.5–2.2 × 10^5^ cells/mL and incubated for 24 h. Cells were then rinsed with PBS and incubated in starvation media (0.5% FBS) overnight. All the controls and drugs were tested in triplicate. The negative control for each drug was prepared using the same DMSO concentration as the drug sample. The drugs were diluted, and the final concentration in each well was 10 µM (or a concentration that did not affect the cell viability of MDA-MB-231 cells). A wound was made using a sterile 200 μL pipette tip. The cells were then gently rinsed with media without FBS, and the negative controls were added to the media. After a 24 h incubation, the gap width was evaluated using Lumera Infinity Analyze 6.4.0 software. Pictures were taken at 0, 8, 12, and 24 h using a 10× objective in a Leica DM IL LED Inverted Laboratory Microscope and an Infinity1-3 3.1 Megapixel USB 2.0 CMOS camera (Leica, Wetzlar, Germany). The percent migration was calculated using the following formulas:

100 − [ (X_0_⁄Ẍ_0_)] × 100 for 0 h measurements

100 − [ (X_24_⁄Ẍ_0_)] × 100 for 24 h measurements

## 4. Conclusions

In this study, a new series of 2-substituted-3-ferrocene-acrylonitrile derivatives was designed, synthesized, and biologically evaluated for their anticancer and anti-migration effects on MCF-7 and MDA-MB-231 cancer cells. Moreover, the geometry of the double bonds in the derivatives was determined using single-crystal X-ray crystallography. Additionally, the electrochemistry of the ferrocene–acrylonitrile derivatives was investigated using cyclic voltammetry. From the results, we identified the structure–activity relationship (SAR), which highlighted the importance of the ferrocenyl group at the 3-position of the acrylonitrile moiety and a substituted phenyl ring at the 2-position (*trans* to the ferrocenyl group) for both antiproliferative and anti-migratory activities. Replacing the ferrocene with a phenyl group led to a loss of activity. The most potent antiproliferative compounds were those bearing 2-phenyl ((*Z*)-**18)** and 2-*p*-F-phenyl ((*Z*)-**28**) groups, which exhibited GI_50_ values between 9.1 and 11.9 μM for MDA-MB-231 cancer cells. The most promising result regarding anti-migratory effects was observed with the ferrocene–acrylonitrile derivative (*Z*)-**25**, which resulted in a 13% migration inhibition for the highly metastatic MDA-MB-231 breast cancer cells. Future studies on structure optimization could enhance these derivatives, and they could assess their effects at higher concentrations and within shorter time frames to minimize any potential cytotoxic effects that may interfere with the inhibition of cell migration. Further investigations into the mechanism of action, focusing on the production of reactive oxygen species (ROS) and hydroxy radicals through reaction with endogenous hydrogen peroxide in cancer cells, are necessary to fully characterize the potential of these compounds as anticancer and anti-metastasis drugs.

## Data Availability

The original contributions presented in this study are included in the article/Supplementary Materials. Further inquiries can be directed to the corresponding author.

## Supplementary Materials

The following supporting information can be downloaded at: https://www.mdpi.com/article/10.3390/molecules30132835/s1, Figure S1: ^1^H NMR spectral data for (*Z*)-**18**; Figure S2: ^13^C{^1^H} NMR spectral data for (*Z*)-**18**; Figure S3: ^1^H NMR spectral data for (*Z*)-**19**; Figure S4: ^13^C{^1^H} NMR spectral data for (*Z*)-**19**; Figure S5: ^1^H NMR spectral data for (*Z*)-**20**; Figure S6: ^13^C{^1^H} NMR spectral data for (*Z*)-**20**; Figure S7: ^1^H NMR spectral data for (*Z*)-**21**; Figure S8: ^13^C{^1^H} NMR spectral data for (*Z*)-**21**; Figure S9: ^1^H NMR spectral data for (*Z*)-**22**; Figure S10: ^13^C{^1^H} NMR spectral data for (*Z*)-**22**; Figure S11: ^1^H NMR spectral data for (*Z*)-**23**; Figure S12: ^13^C{^1^H} NMR spectral data for (*Z*)-**23**; Figure S13: ^1^H NMR spectral data for (*Z*)-**24**; Figure S14: ^13^C{^1^H} NMR spectral data for (*Z*)-**24**; Figure S15: ^1^H NMR spectral data for (*Z*)-**26**; Figure S16: ^13^C{^1^H} NMR spectral data for (*Z*)-**26**; Figure S17: ^1^H NMR spectral data for 28; Figure S18: ^13^C{^1^H} NMR spectral data for 28; Figure S19: ^1^H NMR spectral data for (*Z*)-**35**; Figure S20: ^13^C{^1^H} NMR spectral data for (*Z*)-**35**; Figure S21: The asymmetric unit and crystal packaging of (*Z*)-**27**; Table S1: Important short contacts in the crystal structure (*Z*)-**21**; Table S2: Crystal data and structure refinement for (*Z*)-**21**; Table S3: Bond lengths for (*Z*)-**21**; Table S4: Bond angles for (*Z*)-**21**; Figure S22: Crystal packaging and asymmetric unit of (*Z*)-**27** along the c-axis; Table S5. Important short contacts in the crystal structure of (*Z*)-**27**. Table S6: Crystal data and structure refinement for (*Z*)-**27**; Table S7: Bond lengths for (*Z*)-**27**; Table S8: Bond angles for (*Z*)-**27**; Figure S23: Crystal packaging and asymmetric unit of (*Z*)-**29** along the c-axis; Table S9: Important short contacts in the crystal structure (*Z*)-**29**; Table S10: Crystal data and structure refinement for (*Z*)-**29**; Table S11: Bond lengths for (*Z*)-**29**; Table S12: Bond angles for (*Z*)-**29**; Figure S24: Crystal packaging and asymmetric of (*Z*)-**33** along the c-axis; Table S13: Important short contacts in the crystal structure of (*E*)-**33**; Table S14: Crystal data and structure refinement for (*E*)-**33**; Table S15: Bond lengths for (*E*)-**33**; Table S16: Bond angles for (*E*)-**33**.
