# Supplementary material for: Synthesis, Structure, Electrochemistry, and In Vitro Anticancer and Anti-Migratory Activities of (Z)- and (E)-2-Substituted-3-Ferrocene-Acrylonitrile Hybrids and Their Derivatives
Source: Molecules. 2025 Jul 2;30(13):2835. doi: 10.3390/molecules30132835 (PMC12250641; doi:10.3390/molecules30132835)
Supplement: Supplementary file 1 [file molecules-30-02835-s001.zip › molecules-3652418-supplementary.pdf]

-Supplementary Material-

Synthesis, Structure, Electrochemistry, and In Vitro Anticancer and Anti-Migratory Activities of (*Z*)- and (*E*)-2-Substituted-3-Ferrocene-Acrylonitrile Hybrids and Their Derivatives

William O. Mendoza-Morales [1], Esteban Rodríguez [1], Aliana González [2], Zulma Ramos [1], Jemily Acosta-Mercado [3], Dalice M. Piñero-Cruz [3], Claudia A. Ospina [2], Enrique Meléndez [4] and Eliud Hernández-O'Farrill [1,*]

[1] Department of Pharmaceutical Sciences, School of Pharmacy, University of Puerto Rico, San Juan 00936, Puerto Rico; william.mendoza1@upr.edu (W.O.M.-M.); esteban.rodriguez15@upr.edu (E.R.); zulma.ramos1@upr.edu (Z.R.)

[2] Department of Natural Sciences and Mathematics, Interamerican University of Puerto Rico, Bayamon Campus, Bayamon 00957, Puerto Rico; agonzalez6977@interbayamon.edu (A.G.); cospina@bayamon.inter.edu (C.A.O.)

[3] Department of Chemistry, College of Natural Sciences, University of Puerto Rico, Rio Piedras Campus, San Juan 00925, Puerto Rico; jemily.acosta@upr.edu (J.A.-M.); dalice.pinero@upr.edu (D.M.P.-C.)

[4] Department of Chemistry, University of Puerto Rico, Mayagüez Campus, Mayagüez 00681, Puerto Rico; enrique.melendez@upr.edu

* Correspondence: eliud.hernandez@upr.edu; Tel.: +1-(787)-785-2525 (ext. 5436 or 1236)

Table of Contents

Figure S1: ^{1}H NMR Spectral Data of (*Z*)-18 in $CDCl_3$ 3
Figure S2: ^{13}C{^{1}H} NMR Spectral Data of (*Z*)-18 in $CDCl_3$ 4
Figure S3: ^{1}H NMR Spectral Data of (Z)-19 in $CDCl_3$ 5
Figure S4: ^{13}C{^{1}H} NMR Spectral Data of (*Z*)-19 in $CDCl_3$ 6
Figure S5: ^{1}H NMR Spectral Data of (*Z*)-20 in $CDCl_3$ 7
Figure S6: ^{13}C{^{1}H} NMR Spectral Data of (Z)-20 in $CDCl_3$ 8
Figure S7: ^{1}H NMR Spectral Data of (*Z*)-21 in $CDCl_3$ 9
Figure S8: ^{13}C{^{1}H} NMR Spectral Data of (*Z*)-21 in $CDCl_3$ 10
Figure S9: ^{1}H NMR Spectral Data of (*Z*)-22 in $CDCl_3$ 11
Figure S10: ^{13}C{^{1}H} NMR Spectral Data of (*Z*)-22 in $CDCl_3$ 12
Figure S11: ^{1}H NMR Spectral Data of (*Z*)-23 in $CDCl_3$ 13
Figure S12: ^{13}C{^{1}H} NMR Spectral Data of (*Z*)-23 in $CDCl_3$ 14
Figure S13: ^{1}H NMR Spectral Data of (*Z*)-24 in $CDCl_3$ 15
Figure S14: ^{13}C{^{1}H} NMR Spectral Data of (*Z*)-24 in $CDCl_3$ 16
Figure S15: ^{1}H NMR Spectral Data of (*Z*)-26 in $CDCl_3$ 17
Figure S16: ^{13}C{^{1}H} NMR Spectral Data of (*Z*)-26 in $CDCl_3$ 18
Figure S17: ^{1}H NMR Spectral Data of (*Z*)-28 in $CDCl_3$ 19
Figure S18: ^{13}C{^{1}H} NMR Spectral Data of (*Z*)-28 in $CDCl_3$ 20
Figure S19: ^{1}H NMR Spectral Data of (*Z*)-35 in $CDCl_3$ 21
Figure S20: ^{13}C{^{1}H} NMR Spectral Data of (*Z*)-35 in $CDCl_3$ 22
Figure S21: Crystal structure of compound (*Z*)-21 23
Table S1. Important short contacts in the crystal structure (*Z*)-21 24
Table S2. Crystal data and structure refinement for (*Z*)-21 25
Table S3. Bond Lengths for (Z)-21 26
Table S4. Bond Angles for (Z)-21 27
Figure S22: Crystal structure of compound (*Z*)-27 29
Table S5. Important short contacts in the crystal structure of (Z)-27 30
Table S6. Crystal data and structure refinement for (*Z*)-27 31
Table S7. Bond Lengths for (Z)-27 32
Table S8. Bond Angles for (Z)-27 34
Figure S23: Crystal structure of compound (*Z*)-29 37
Table S9. Important short contacts in the crystal structure of (*Z*)-29 38
Table S10. Crystal data and structure refinement for (*Z*)-29 39
Table S11. Bond Lengths for (*Z*)-29 40
Table S12. Bond Angles for (*Z*)-29 42
Figure S24: Crystal structure of compound (*E*)-**33** 46
Table S13. Important short contacts in the crystal structure (*E*)-33 47
Table S14. Crystal data and structure refinement for (*E*)-33 48
Table S15. Bond Lengths for (*E*)-33 49
Table S16. Bond Angles for (*E*)-33 50

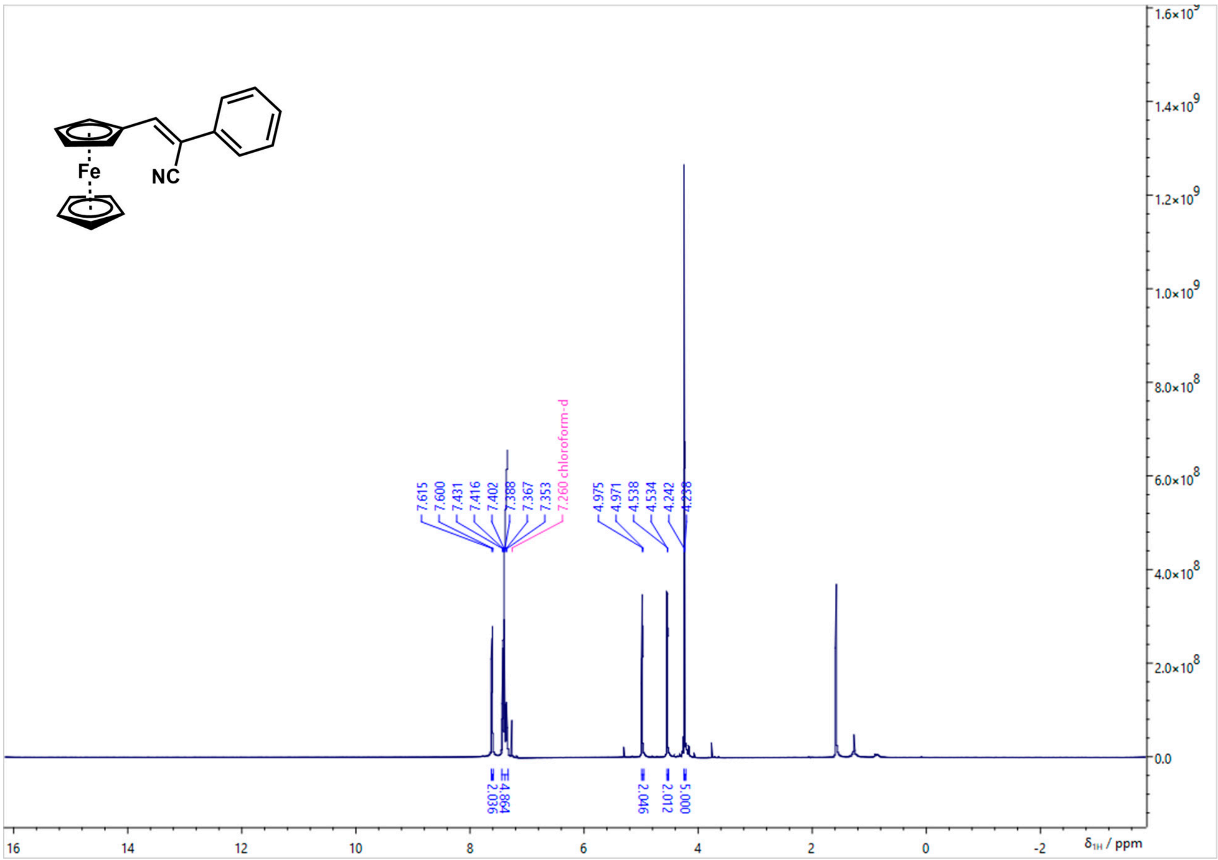

Figure S1: ^{1}H NMR Spectral Data of (*Z*)-**18** in $CDCl_3$

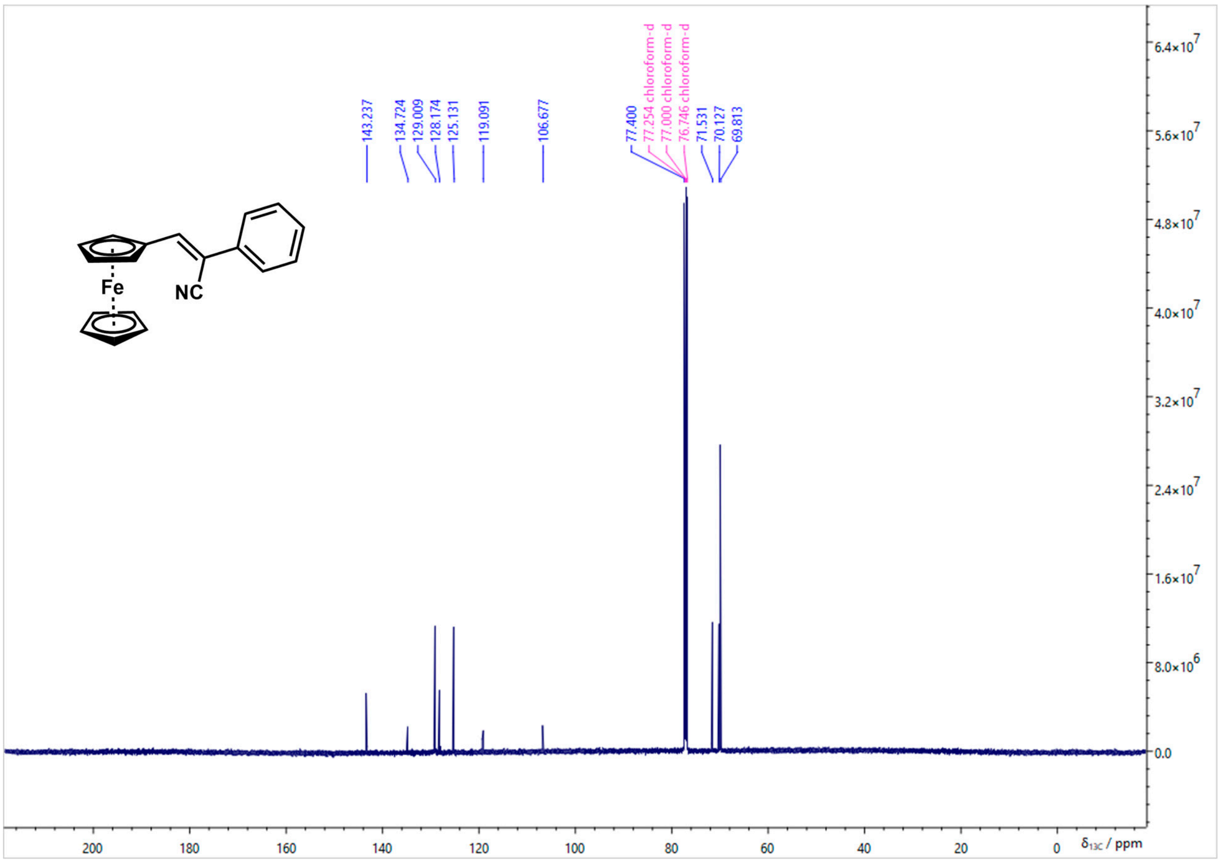

Figure S2: $^{13}C\{^{1}H\}$ NMR Spectral Data of (*Z*)-**18** in $CDCl_3$

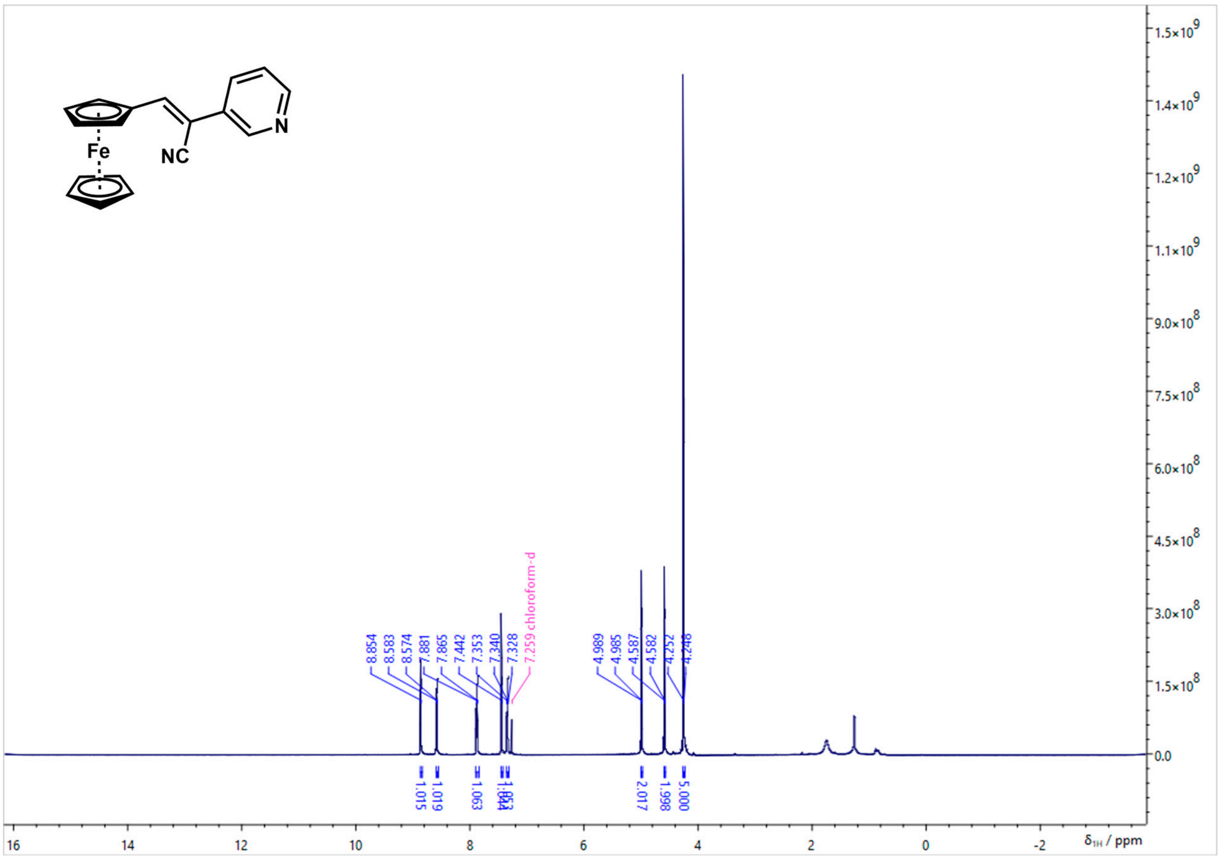

Figure S3: ^{1}H NMR Spectral Data of (*Z*)-**19** in $CDCl_3$

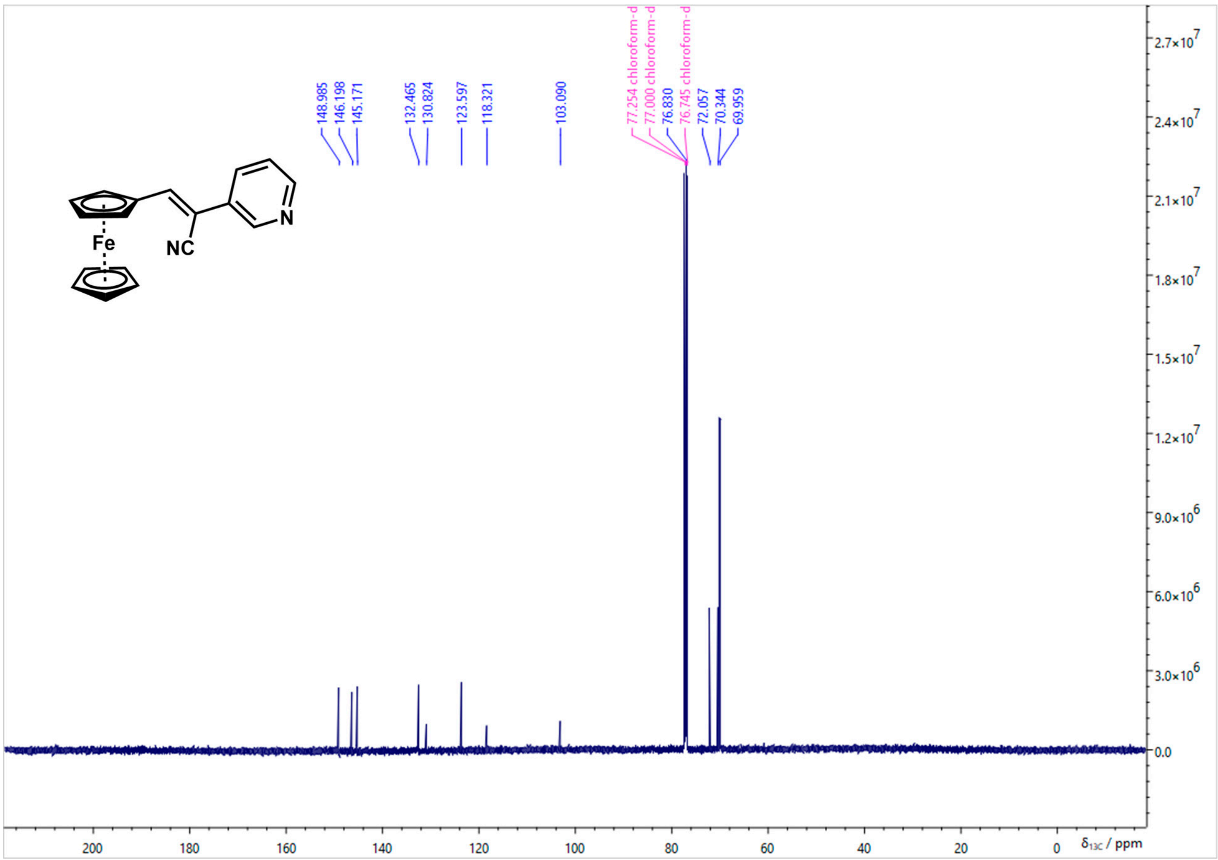

Figure S4: $^{13}C\{^1H\}$ NMR Spectral Data of (*Z*)-**19** in $CDCl_3$

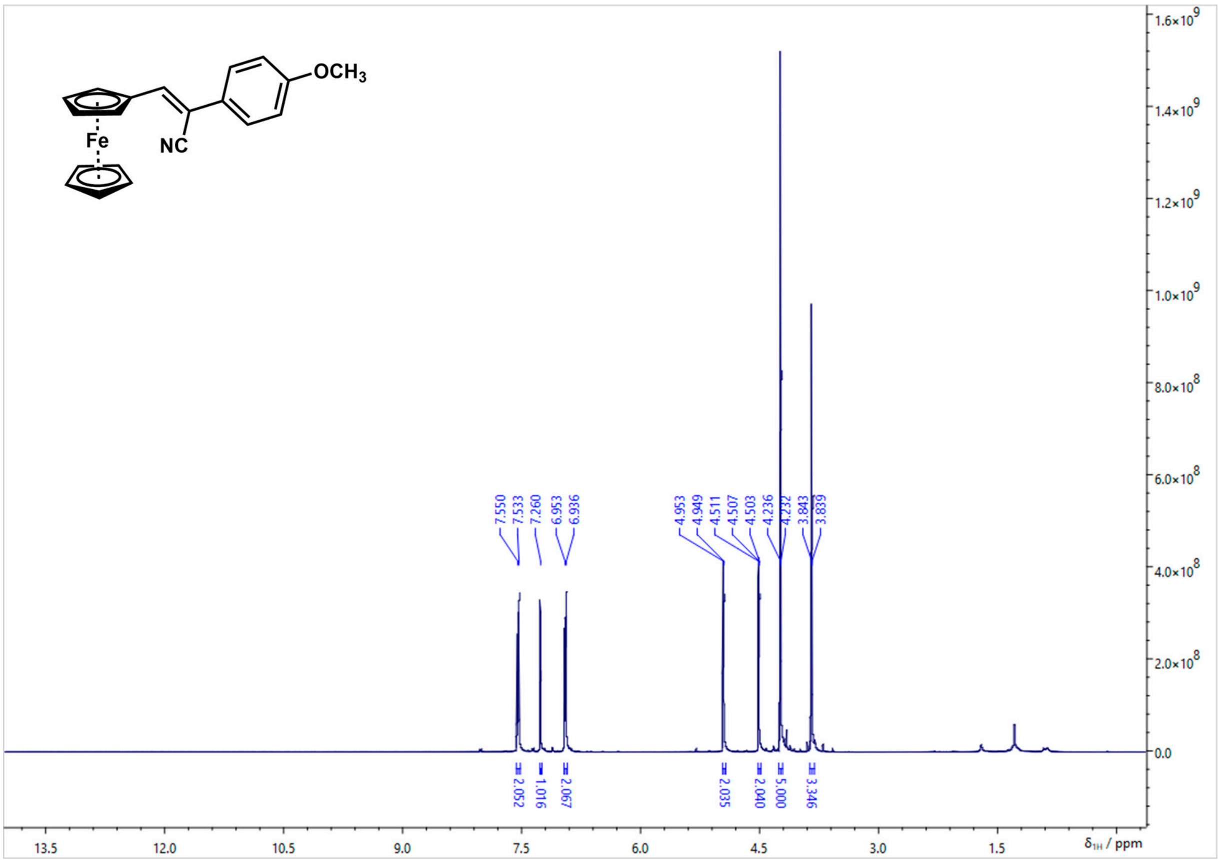

Figure S5: ^{1}H NMR Spectral Data of (*Z*)-**20** in $CDCl_3$

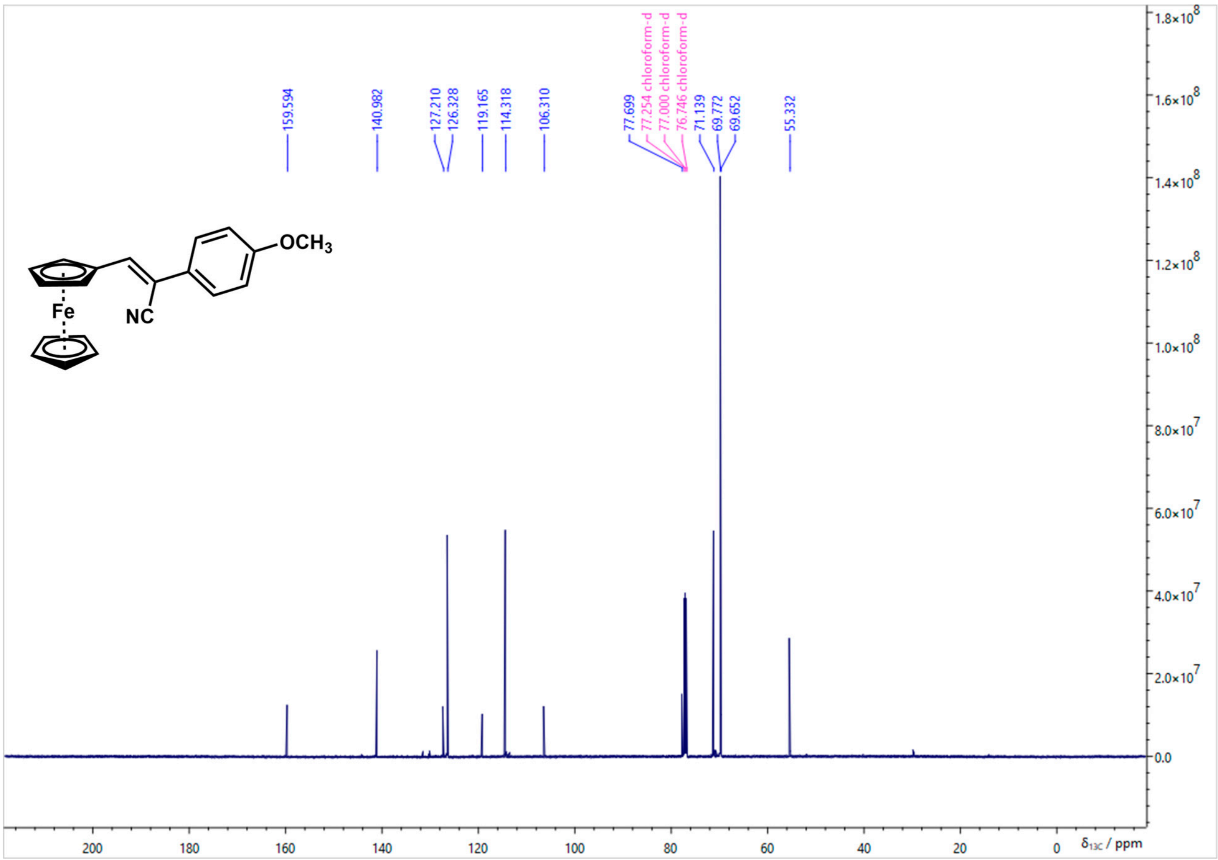

Figure S6: $^{13}C\{^{1}H\}$ NMR Spectral Data of (*Z*)-**20** in $CDCl_3$

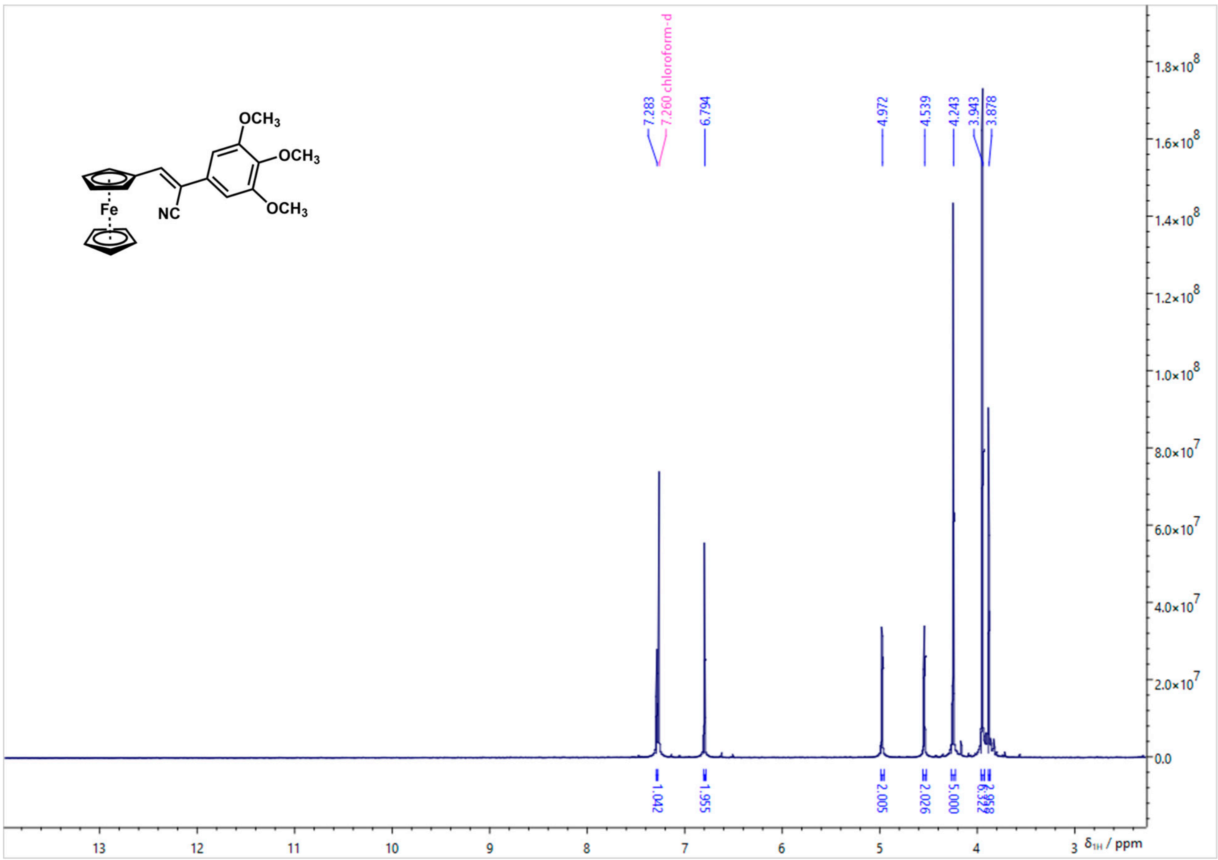

Figure S7: ^{1}H NMR Spectral Data of (*Z*)-**21** in $CDCl_3$

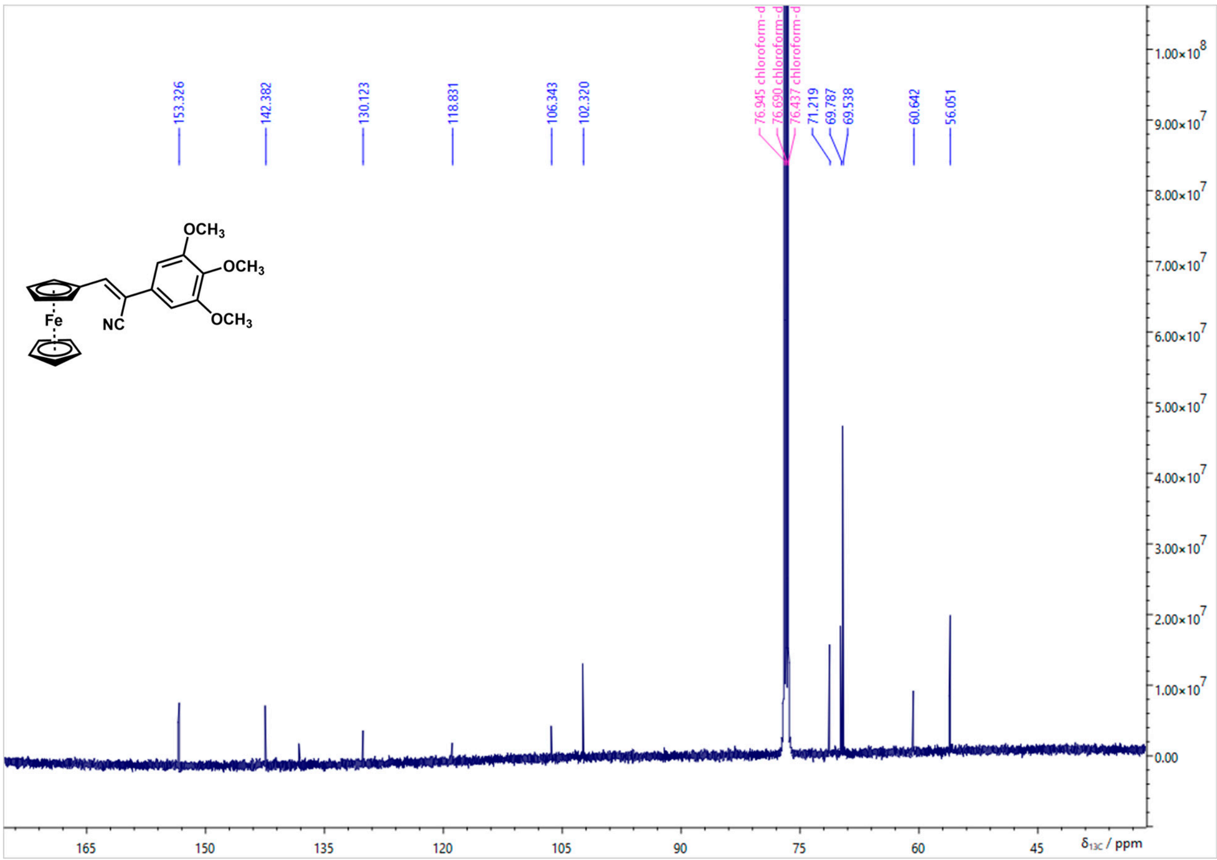

Figure S8: $^{13}C\{^{1}H\}$ NMR Spectral Data of (*Z*)-**21** in $CDCl_3$

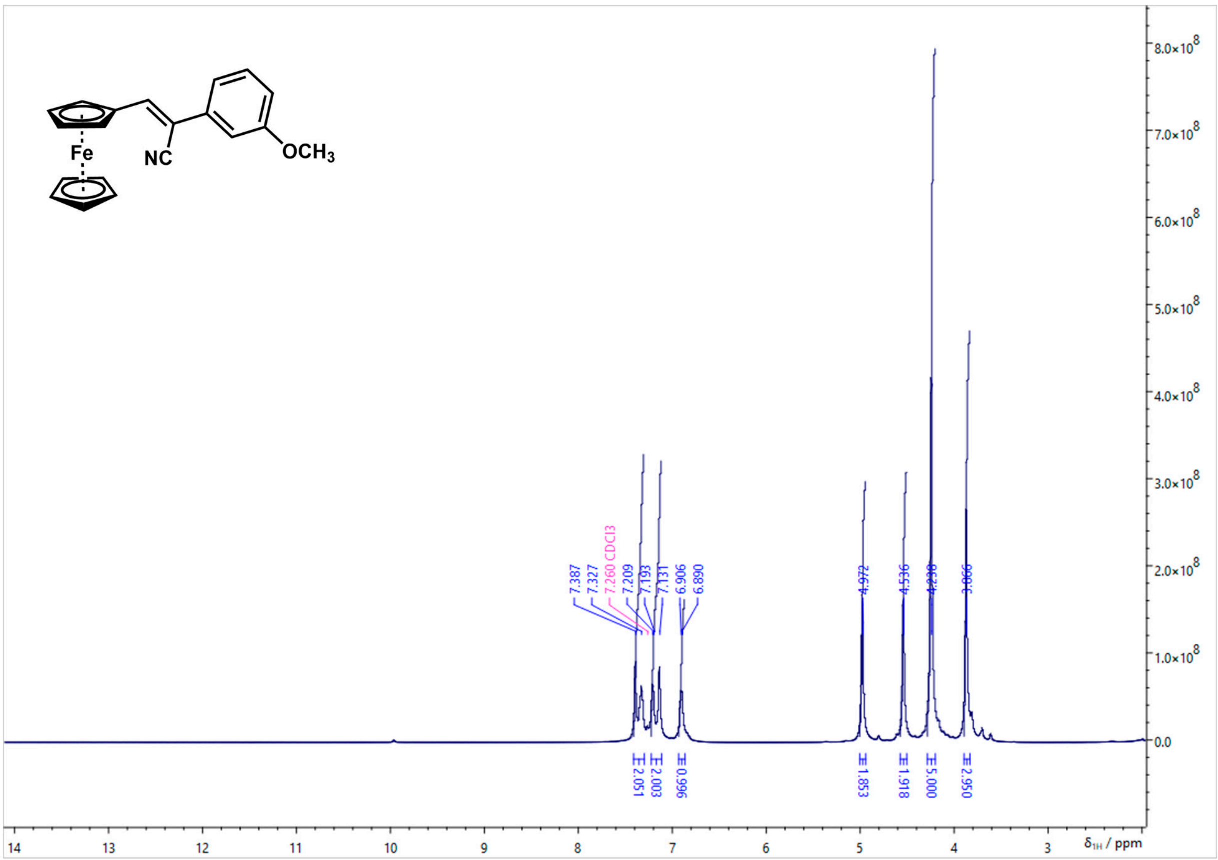

Figure S9: ^{1}H NMR Spectral Data of (*Z*)-**22** in $CDCl_3$

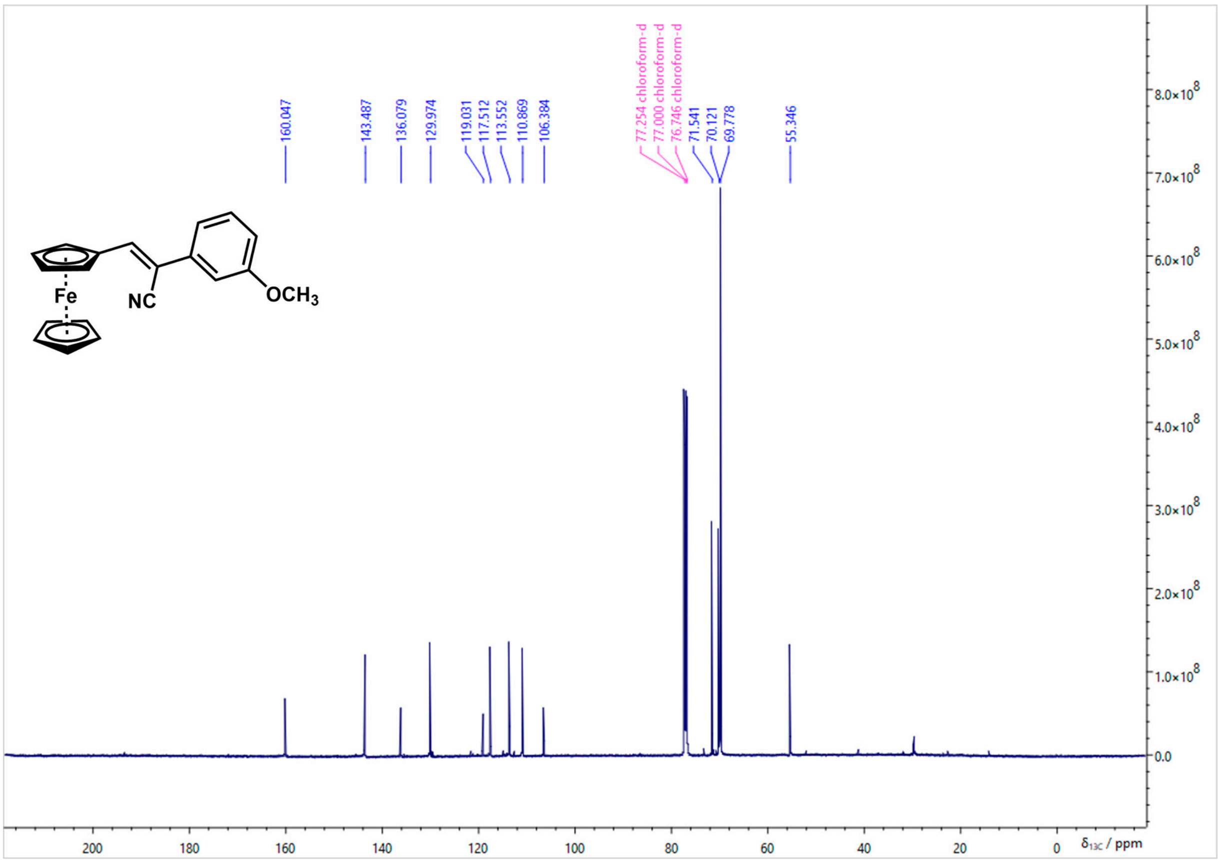

Figure S10: $^{13}C\{^{1}H\}$ NMR Spectral Data of (*Z*)-**22** in $CDCl_3$

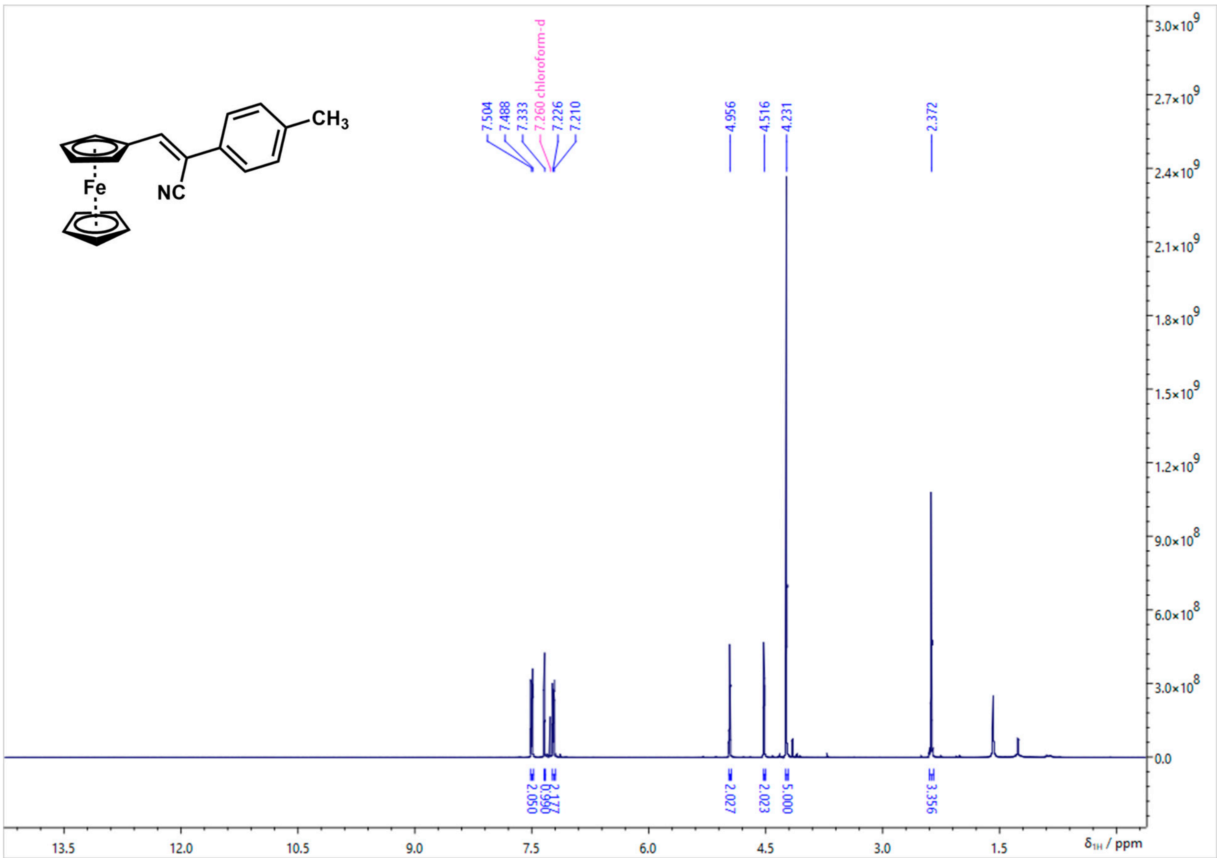

Figure S11: ^{1}H NMR Spectral Data of (*Z*)-**23** in $CDCl_3$

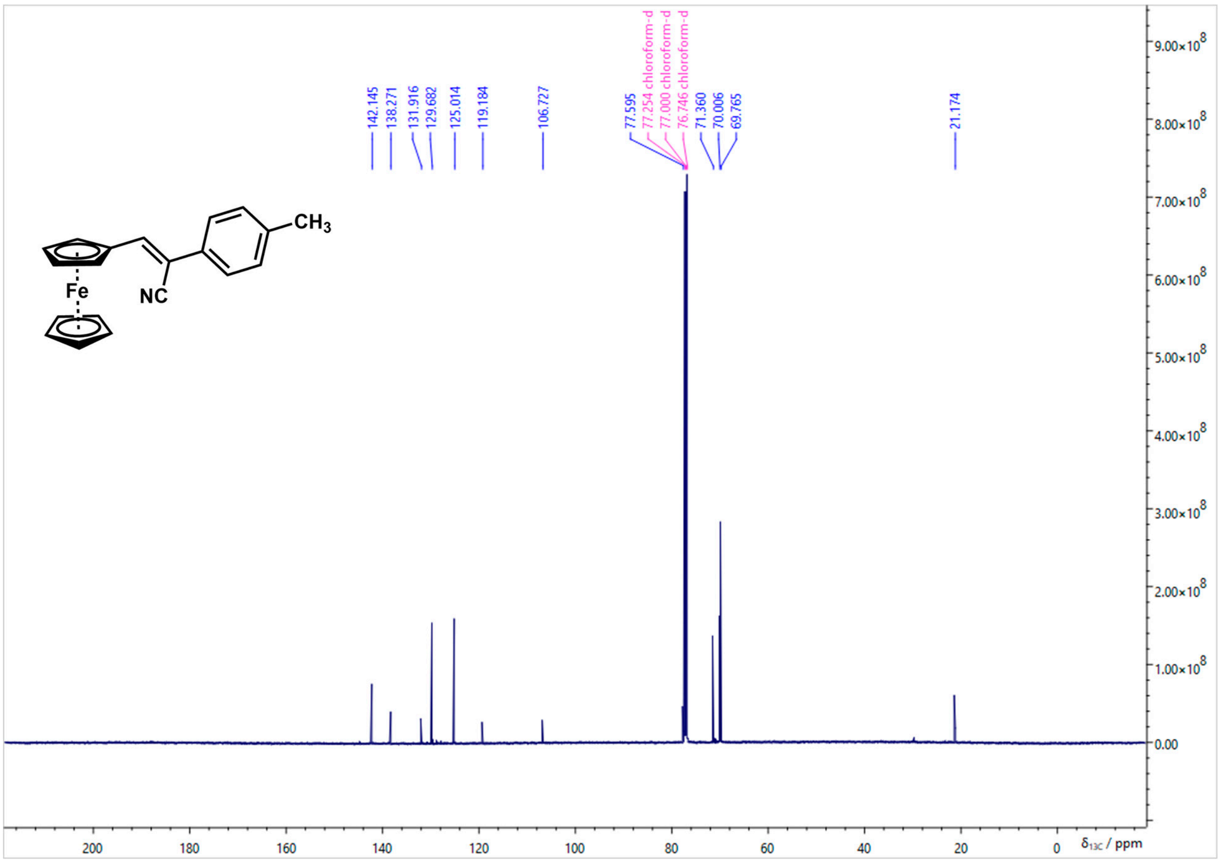

Figure S12: $^{13}C\{^1H\}$ NMR Spectral Data of (*Z*)-**23** in $CDCl_3$

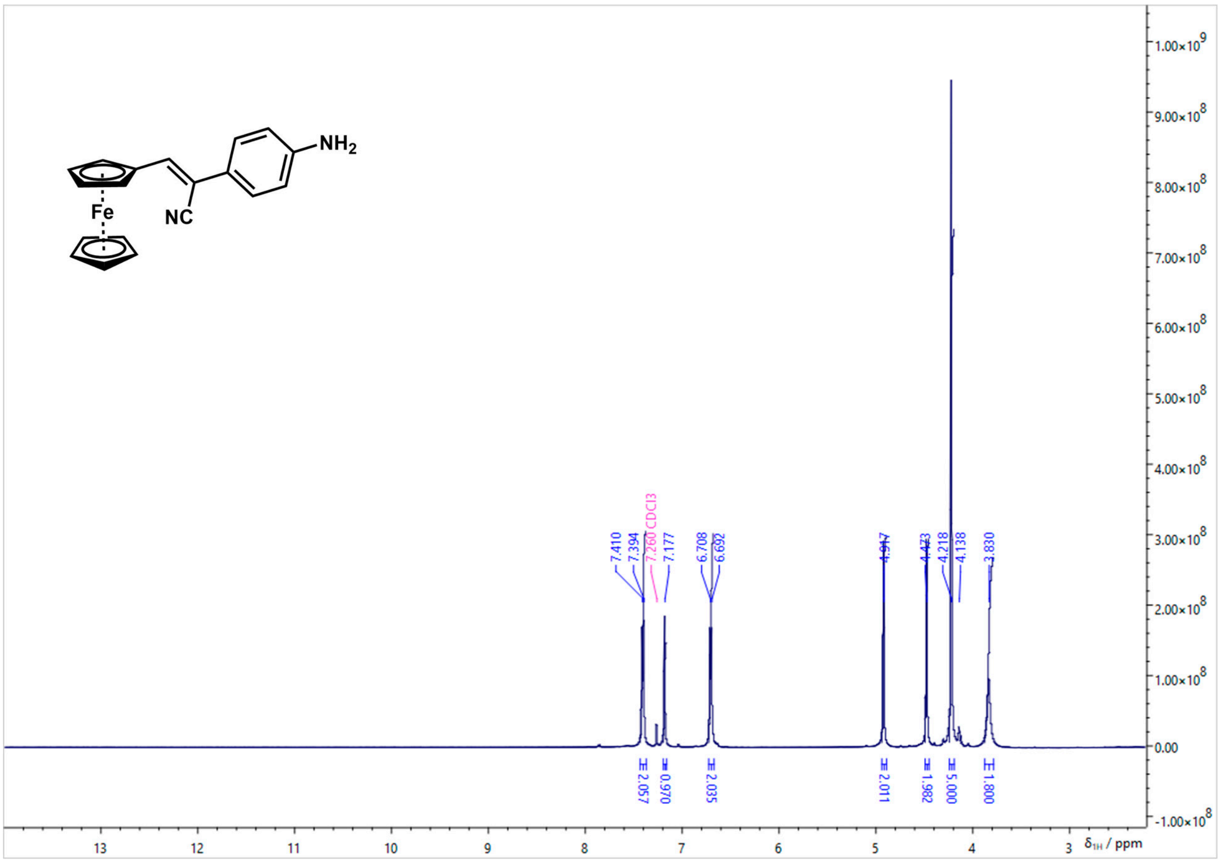

Figure S13: ^{1}H NMR Spectral Data of (*Z*)-**24** in $CDCl_3$

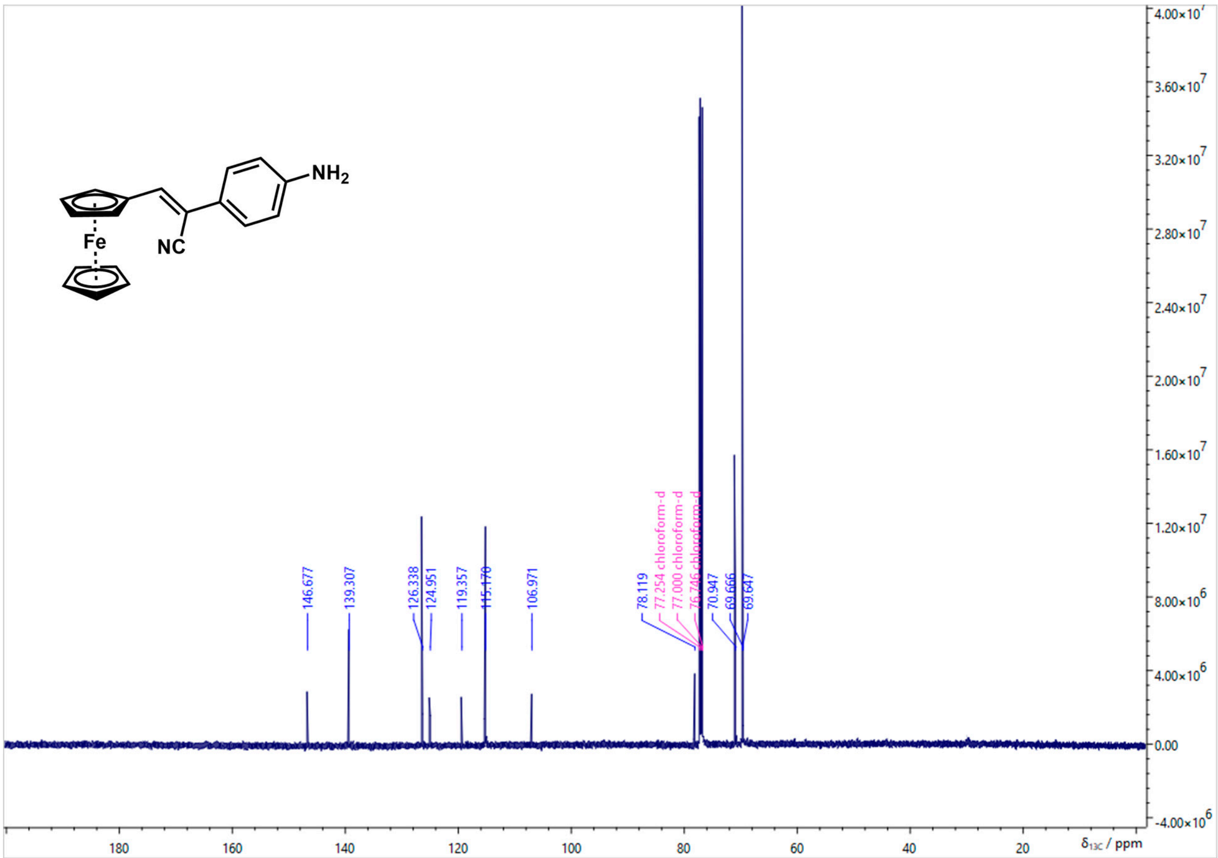

Figure S14: $^{13}C\{^{1}H\}$ NMR Spectral Data of (*Z*)-**24** in $CDCl_3$

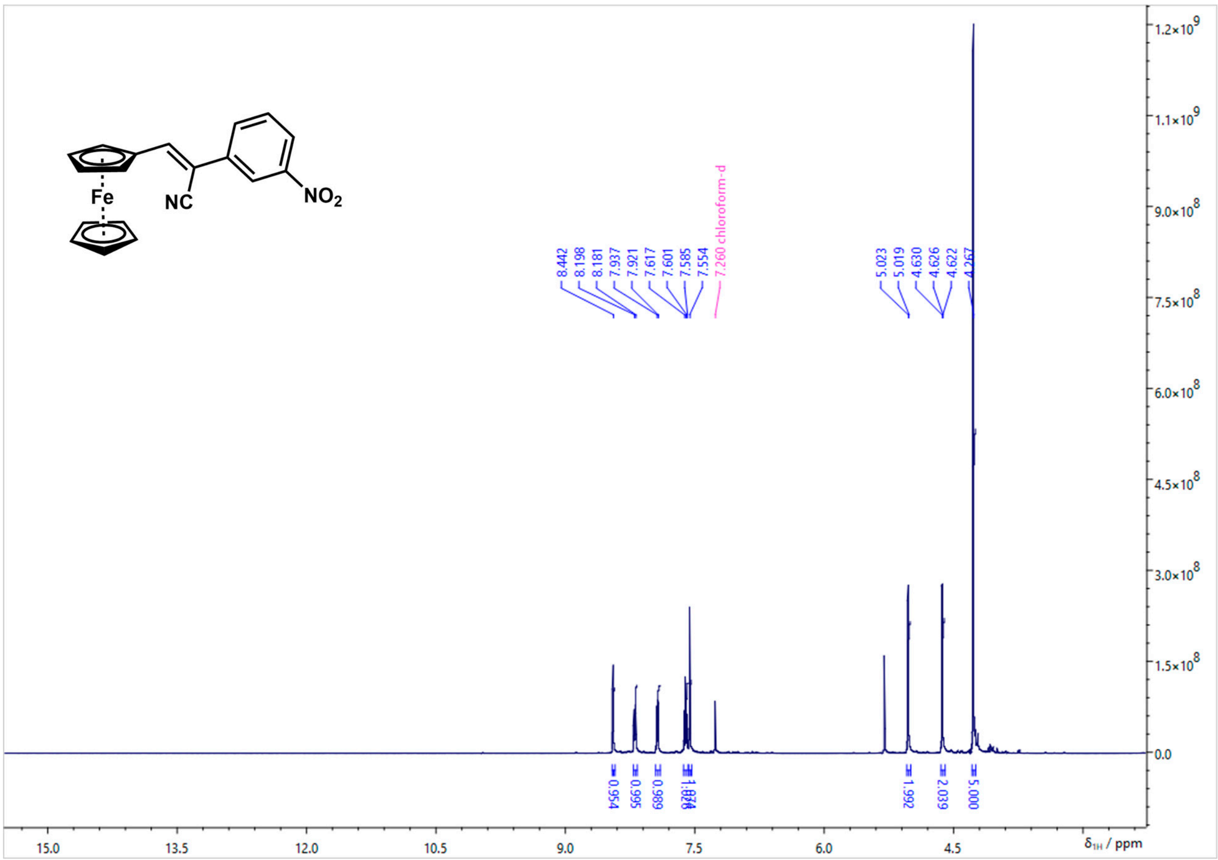

Figure S15: ^{1}H NMR Spectral Data of (*Z*)-**26** in $CDCl_3$

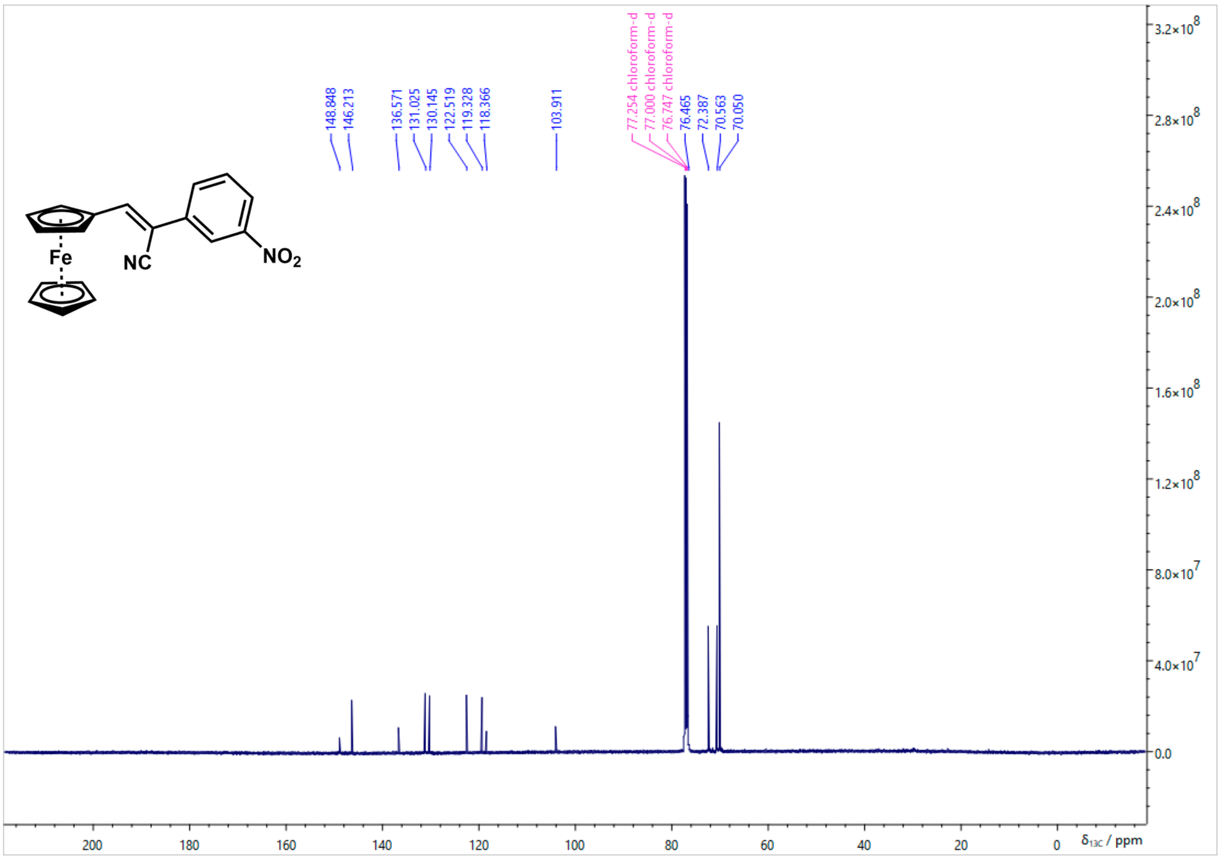

Figure S16: $^{13}C\{^{1}H\}$ NMR Spectral Data of (*Z*)-**26** in $CDCl_3$

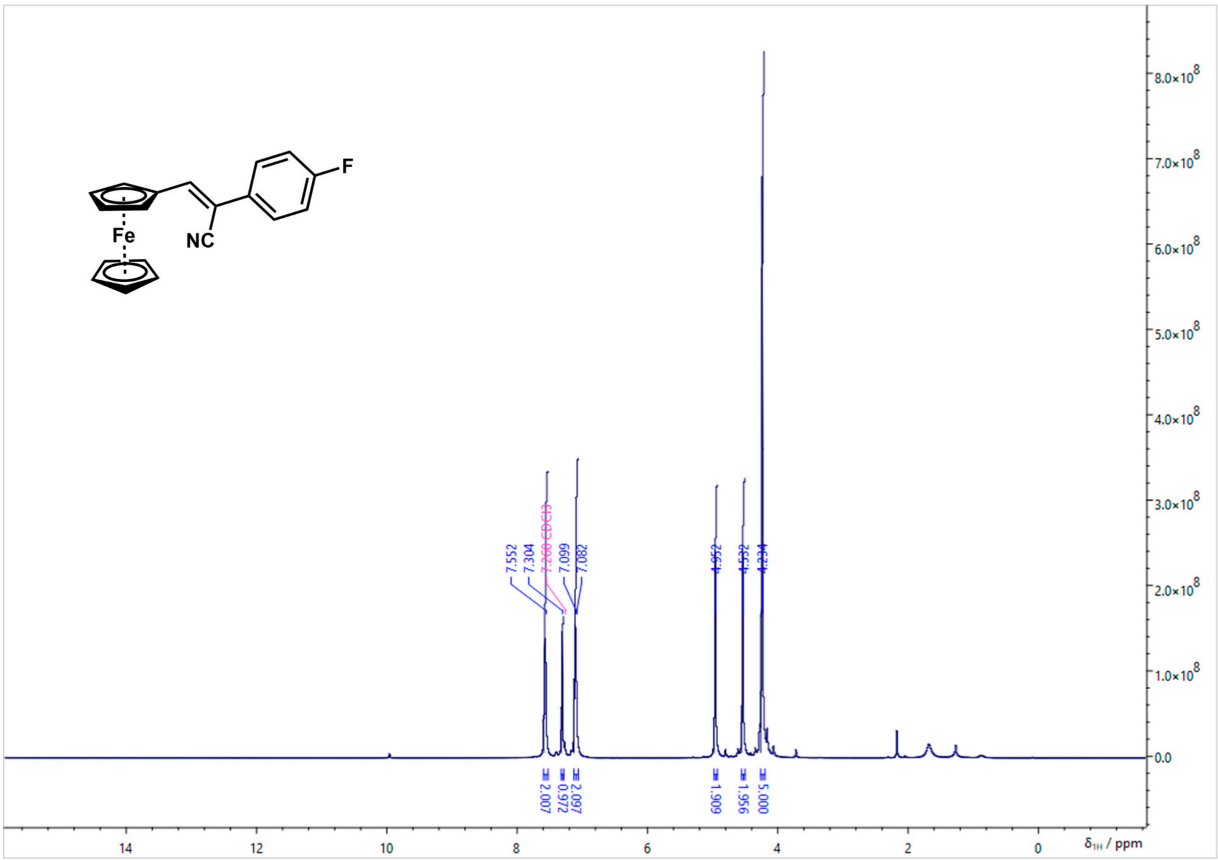

Figure S17: ^{1}H NMR Spectral Data of (*Z*)-**28** in $CDCl_3$

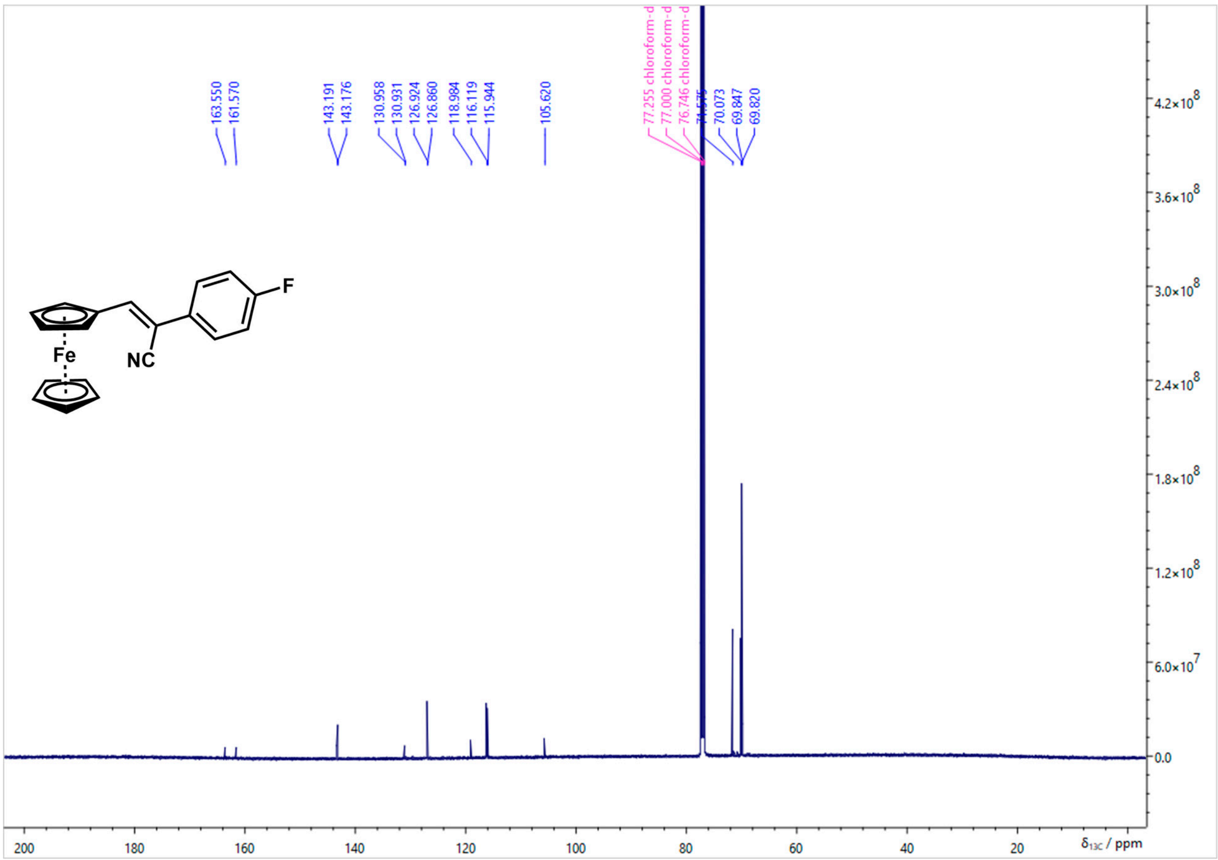

Figure S18: $^{13}C\{^{1}H\}$ NMR Spectral Data of (*Z*)-**28** in $CDCl_3$

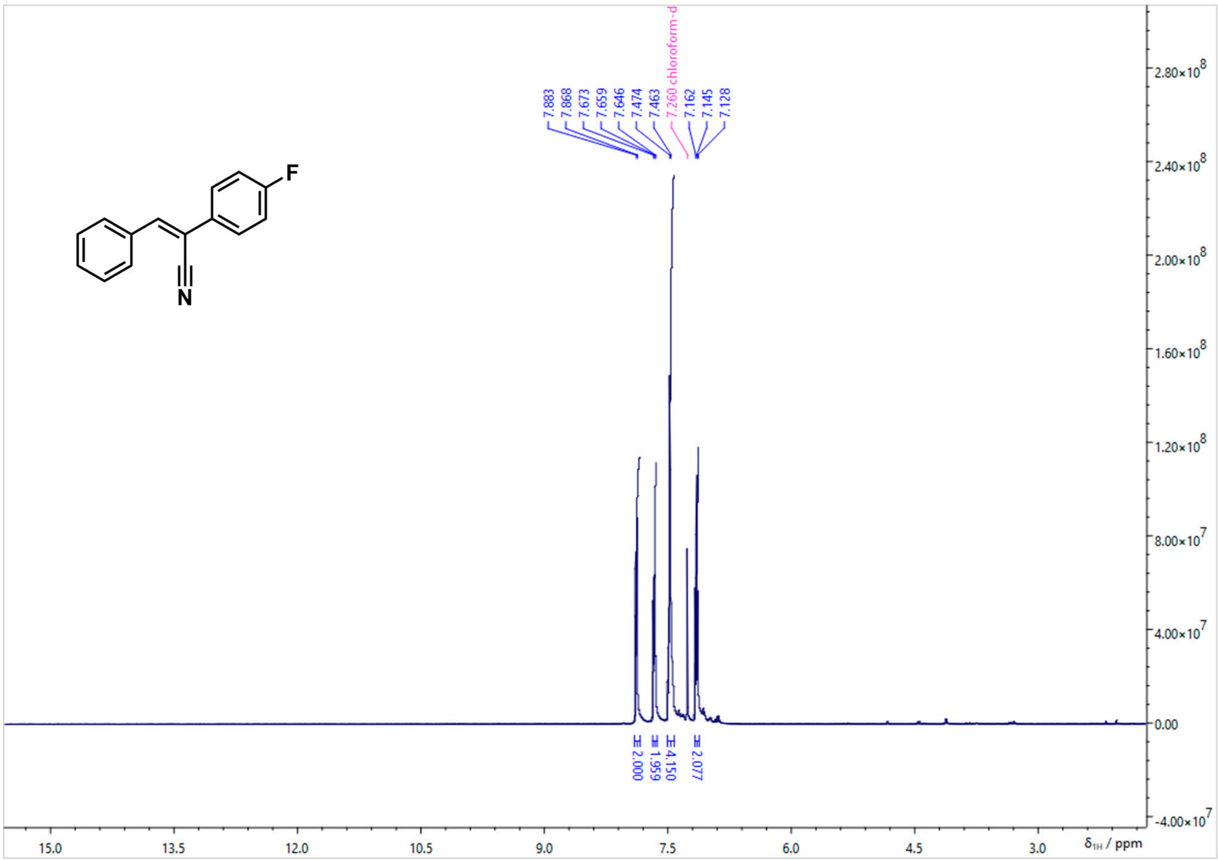

Figure S19: ^{1}H NMR Spectral Data of (*Z*)-**35** in $CDCl_3$

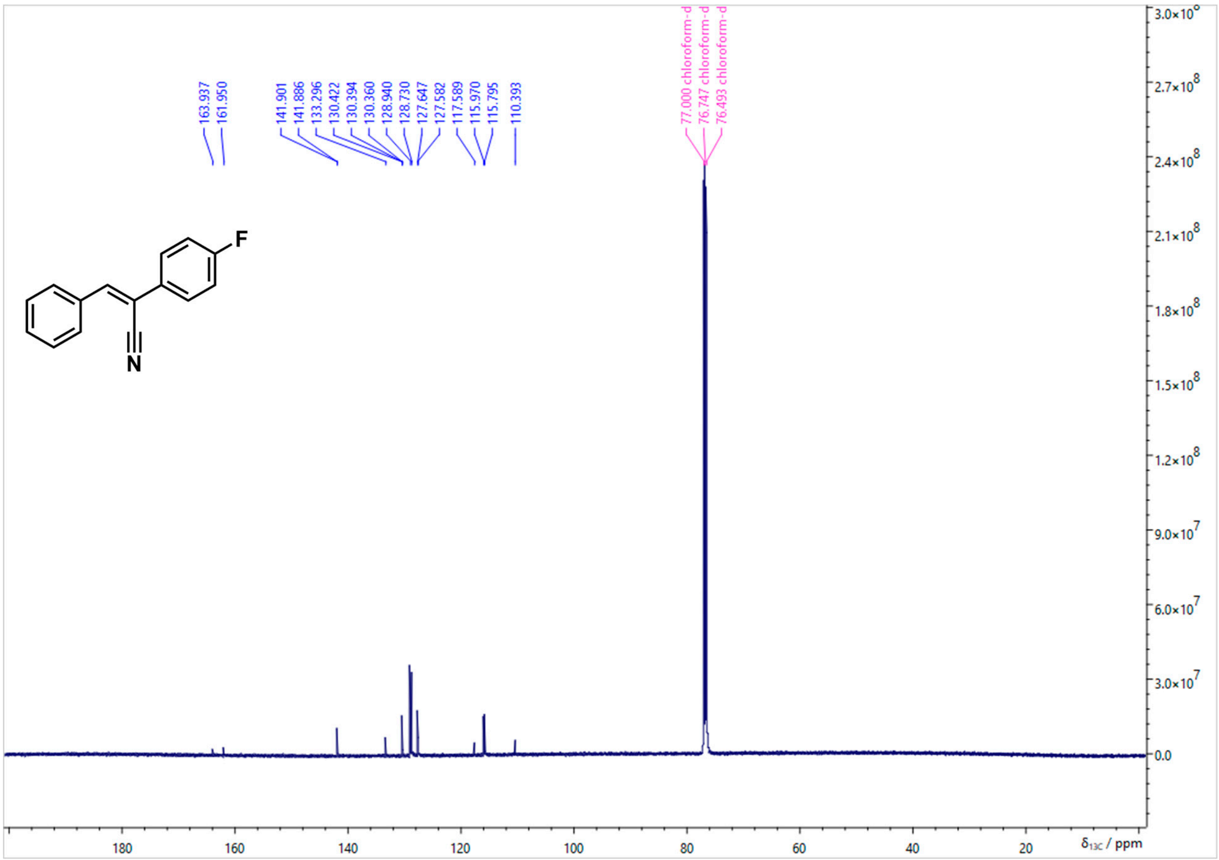

Figure S20: $^{13}C\{^{1}H\}$ NMR Spectral Data of (*Z*)-**35** in $CDCl_3$

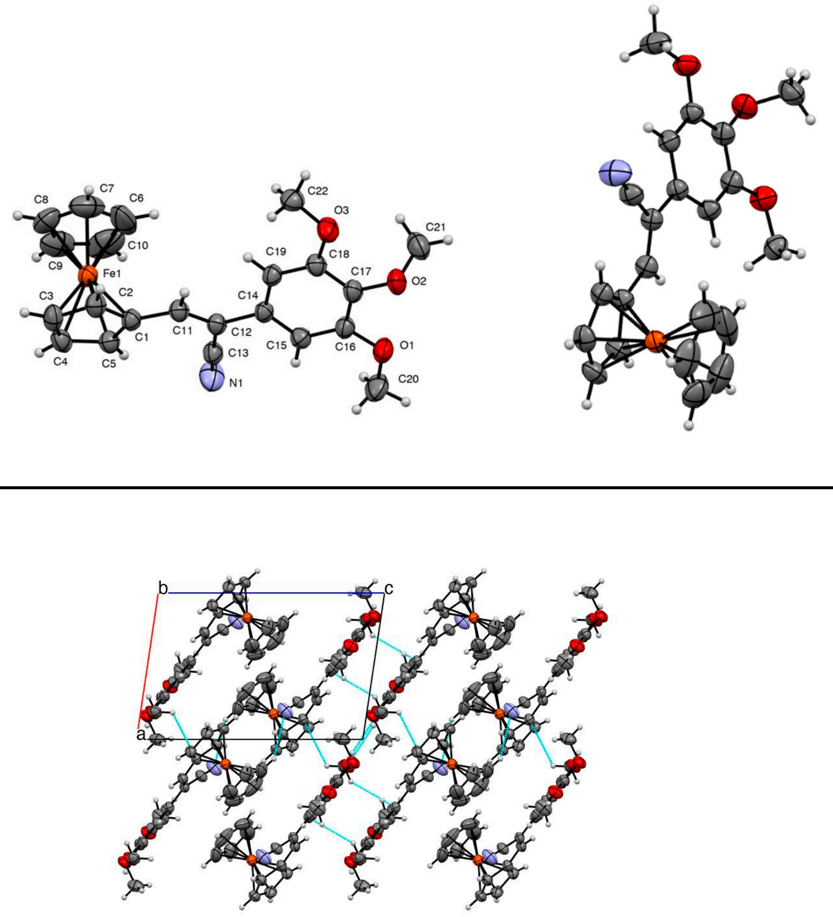

Figure S21: Crystal structure of compound *(Z)*-**21** with labels for non-C/H atoms and ellipsoids at the 50% probability level for (top left) a single molecular unit, (top right) the asymmetric unit, and (bottom) crystal packing along the ***b*** axis.

The compound crystallizes under the triclinic space group P-1. The substituent in the ferrocene subunit lies co-planar to the cyclopentadienyl ring. Important intermolecular interactions are observed between nitrogen N1 from the nitrile substituent and H9; C1 with H22c; C14 with H22a; and oxygen O3 with H22b, suggesting a network of weak hydrogen bonding and van der Waals interactions that may contribute to the stabilization of the molecular packing. Important short contacts are presented in the following list (Table S1). Table S2 summarizes the crystal data and structure refinement parameters for compound *(Z)*-**21**. Table S3 lists the bond lengths, and Table S4 lists the bond angles observed in *(Z)*-21. The CCDC number for (*Z*)-**21** is 2455357.

Table S1. Important short contacts in the crystal structure (*Z*)-21.

Atom1	Atom 2	Length
C1	H22c	2.885
N1	H9	2.739
C14	H22a	2.818
O3	H22b	2.659

Table S2. Crystal data and structure refinement for (*Z*)-21.

Identification code	dpc296_auto
Empirical formula	$C_{22}H_{21}FeNO_3$
Formula weight	403.25
Temperature/K	300(2)
Crystal system	triclinic
Space group	P-1
a/Å	8.09661(13)
b/Å	9.56240(16)
c/Å	12.3539(2)
α/°	91.5680(13)
β/°	98.2599(13)
γ/°	95.0197(13)
Volume/Å^3	942.15(3)
Z	2
ρ_{calc}g/cm^3	1.421
μ/mm^{-1}	6.597
F(000)	420
Crystal size/mm^3	0.17 × 0.13 × 0.03
Radiation	Cu Kα (λ = 1.54184)
2Θ range for data collection/°	7.24 to 136.92
Index ranges	$-9 \leq h \leq 9$, $-10 \leq k \leq 11$, $-14 \leq l \leq 14$
Reflections collected	13453
Independent reflections	3472 [R_{int} = 0.0397, R_{sigma} = 0.0419]
Data/restraints/parameters	3472/0/247
Goodness-of-fit on F^2	1.03
Final R indexes [I>=2σ (I)]	R_1 = 0.0325, wR_2 = 0.0846
Final R indexes [all data]	R_1 = 0.0363, wR_2 = 0.0870
Largest diff. peak/hole / e Å^{-3}	0.26/-0.26

Table S3. Bond Lengths for (Z)-21.					
**Atom**	**Atom**	**Length/Å**	**Atom**	**Atom**	**Length/Å**
Fe1	C1	2.0437(18)	C1	C11	1.449(2)
Fe1	C2	2.037(2)	C2	C3	1.414(3)
Fe1	C3	2.055(2)	C3	C4	1.415(3)
Fe1	C4	2.045(2)	C4	C5	1.417(3)
Fe1	C5	2.037(2)	C6	C7	1.378(5)
Fe1	C6	2.032(3)	C6	C10	1.415(5)
Fe1	C7	2.038(3)	C7	C8	1.367(4)
Fe1	C8	2.052(2)	C8	C9	1.391(4)
Fe1	C9	2.037(2)	C9	C10	1.414(5)
Fe1	C10	2.024(3)	C11	C12	1.344(3)
O1	C16	1.364(2)	C12	C13	1.432(3)
O1	C20	1.415(3)	C12	C14	1.490(2)
O2	C17	1.379(2)	C14	C15	1.390(3)
O2	C21	1.426(3)	C14	C19	1.389(3)
O3	C18	1.363(2)	C15	C16	1.391(3)
O3	C22	1.425(3)	C16	C17	1.388(3)
N1	C13	1.141(3)	C17	C18	1.391(3)
C1	C2	1.433(3)	C18	C19	1.385(3)
C1	C5	1.433(3)			

Table S4. Bond Angles for (Z)-21.							
**Atom**	**Atom**	**Atom**	**Angle/°**	**Atom**	**Atom**	**Atom**	**Angle/°**
C2	Fe1	C1	41.11(8)	C5	C1	C2	106.82(17)
C3	Fe1	C1	68.75(8)	C11	C1	Fe1	124.88(13)
C3	Fe1	C2	40.44(8)	C11	C1	C2	121.75(18)
C4	Fe1	C1	68.65(8)	C11	C1	C5	131.40(19)
C4	Fe1	C2	68.07(9)	C1	C2	Fe1	69.69(11)
C4	Fe1	C3	40.37(9)	C3	C2	Fe1	70.48(12)
C5	Fe1	C1	41.12(8)	C3	C2	C1	108.75(19)
C5	Fe1	C2	68.78(9)	C2	C3	Fe1	69.09(11)
C5	Fe1	C3	68.47(9)	C4	C3	Fe1	69.40(12)
C5	Fe1	C4	40.63(8)	C4	C3	C2	107.70(18)
C6	Fe1	C1	109.12(11)	C3	C4	Fe1	70.22(12)
C6	Fe1	C2	122.96(14)	C5	C4	Fe1	69.40(11)
C6	Fe1	C3	157.20(18)	C5	C4	C3	108.78(18)
C6	Fe1	C4	161.80(18)	C1	C5	Fe1	69.68(11)
C6	Fe1	C5	125.84(15)	C4	C5	Fe1	69.96(12)
C7	Fe1	C1	126.57(11)	C4	C5	C1	107.94(19)
C7	Fe1	C2	109.78(12)	C7	C6	Fe1	70.46(17)
C7	Fe1	C3	122.25(14)	C10	C6	Fe1	69.31(17)
C7	Fe1	C4	155.97(14)	C10	C6	C7	107.2(3)
C7	Fe1	C5	162.90(13)	C6	C7	Fe1	69.96(18)
C7	Fe1	C6	39.58(16)	C8	C7	Fe1	71.04(16)
C8	Fe1	C1	161.80(11)	C8	C7	C6	109.9(3)
C8	Fe1	C2	124.78(11)	C7	C8	Fe1	69.91(16)
C8	Fe1	C3	107.74(11)	C9	C8	Fe1	69.54(15)
C8	Fe1	C4	121.03(10)	C9	C8	C7	108.3(3)
C8	Fe1	C5	155.84(11)	C8	C9	Fe1	70.68(15)
C8	Fe1	C6	66.76(13)	C10	C9	Fe1	69.14(16)
C8	Fe1	C7	39.05(12)	C10	C9	C8	107.6(3)
C9	Fe1	C1	157.16(12)	C6	C10	Fe1	69.85(18)
C9	Fe1	C2	159.79(12)	C9	C10	Fe1	70.12(16)
C9	Fe1	C3	123.06(12)	C9	C10	C6	107.0(3)
C9	Fe1	C4	106.69(10)	C12	C11	C1	130.06(19)
C9	Fe1	C5	120.83(11)	C13	C12	C11	120.52(16)

C9	Fe1	C6	67.94(14)	C14	C12	C11	124.08(17)
C9	Fe1	C7	66.57(13)	C14	C12	C13	115.39(17)
C9	Fe1	C8	39.78(12)	C12	C13	N1	177.5(2)
C10	Fe1	C1	122.00(12)	C15	C14	C12	119.72(17)
C10	Fe1	C2	158.32(15)	C19	C14	C12	120.56(17)
C10	Fe1	C3	159.87(16)	C19	C14	C15	119.72(17)
C10	Fe1	C4	123.82(15)	C16	C15	C14	120.13(19)
C10	Fe1	C5	107.43(12)	C15	C16	O1	124.38(19)
C10	Fe1	C6	40.84(16)	C17	C16	O1	115.51(17)
C10	Fe1	C7	67.22(16)	C17	C16	C15	120.11(18)
C10	Fe1	C8	67.45(13)	C16	C17	O2	120.24(18)
C10	Fe1	C9	40.74(14)	C18	C17	O2	120.22(18)
C20	O1	C16	117.58(16)	C18	C17	C16	119.52(17)
C21	O2	C17	113.41(17)	C17	C18	O3	114.81(16)
C22	O3	C18	117.82(15)	C19	C18	O3	124.73(18)
C2	C1	Fe1	69.20(11)	C19	C18	C17	120.46(19)
C5	C1	Fe1	69.19(11)	C18	C19	C14	120.04(18)

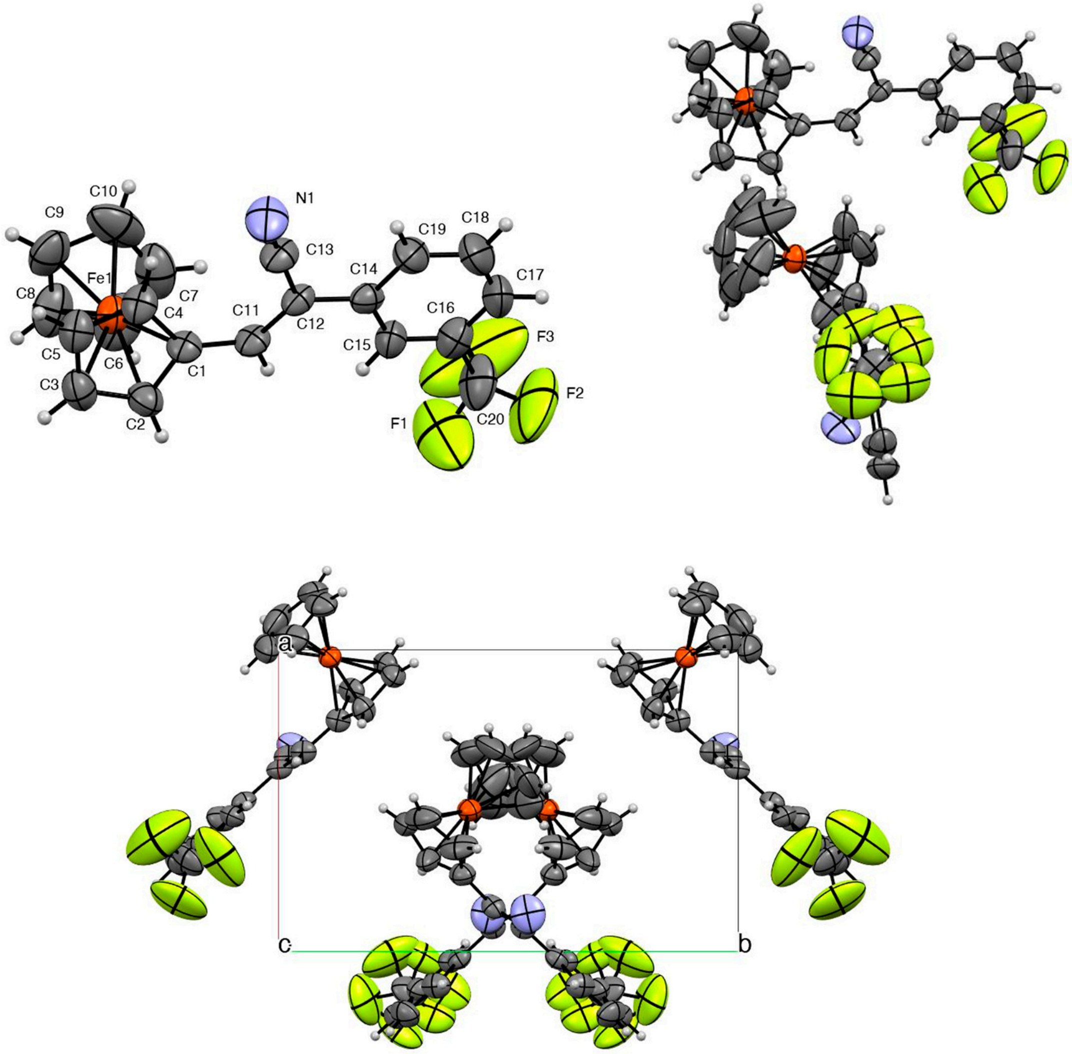

Figure S22: Crystal structure of compound (Z)-**27** with labels for non-C/H atoms and ellipsoids at the 50% probability level for (top left) a single molecular unit, (top right) the asymmetric unit, and (bottom) crystal packing along the ***c*** axis.

The compound crystallizes under the monoclinic space group Pc. The substituent in the ferrocene subunit lays co-planar to the cyclopentadienyl ring. Important intermolecular interactions are observed between nitrogen N1, from the nitrile substituent, with C11 and C15, and with C7…C35 from the CF3 substituents. Important short contacts are presented in the following list (Table S6). Table S7 summarizes the crystal data and structure refinement parameters for compound (Z)-**27**. Table S8 lists the bond lengths, and Table S9 the bond angles observed in (Z)-**27**. The CCDC number for (Z)-**27** is 2455367.

Table S5. Important short contacts in the crystal structure of (*Z*)-27.

Atom1	Atom2	Length
H11	N1	2.578
H15	N1	2.641
H17	C35	2.873
N2	H31	2.451
N2	H35	2.655

Table S6. Crystal data and structure refinement for (*Z*)-27.	
Identification code	dpc314_auto
Empirical formula	$C_{20}H_{14}F_3FeN$
Formula weight	381.17
Temperature/K	300(2)
Crystal system	monoclinic
Space group	Pc
a/Å	9.2775(3)
b/Å	14.0923(4)
c/Å	13.1862(4)
α/°	90
β/°	96.077(3)
γ/°	90
Volume/Å^3	1714.29(9)
Z	4
ρ_{calc}g/cm^3	1.477
μ/mm^{-1}	7.344
F(000)	776
Crystal size/mm^3	0.62 × 0.37 × 0.26
Radiation	CuKα (λ = 1.54184)
2Θ range for data collection/°	6.272 to 136.696
Index ranges	-11 ≤ h ≤ 9, -15 ≤ k ≤ 16, -14 ≤ l ≤ 15
Reflections collected	8362
Independent reflections	6288 [R_{int} = 0.0367, R_{sigma} = 0.0468]
Data/restraints/parameters	6288/14/480
Goodness-of-fit on F^2	1.032
Final R indexes [I>=2σ (I)]	R_1 = 0.0632, wR_2 = 0.1737
Final R indexes [all data]	R_1 = 0.0688, wR_2 = 0.1790
Largest diff. peak/hole / e Å^{-3}	0.65/-0.46
Flack parameter	0.007(8)

Table S7. Bond Lengths for (Z)-27.

Atom	Atom	Length/Å	Atom	Atom	Length/Å
Fe1	C20	2.043(8)	Fe2	C29	2.035(15)
Fe1	C2	1.993(9)	Fe2	C28	2.002(17)
Fe1	C3	2.034(9)	Fe2	C30	1.987(14)
Fe1	C4	2.048(8)	N2	C33	1.124(12)
Fe1	C5	2.027(8)	C21	C22	1.425(17)
Fe1	C6	2.035(11)	C21	C25	1.413(14)
Fe1	C7	2.049(11)	C21	C31	1.422(12)
Fe1	C8	2.039(10)	C22	C23	1.413(18)
Fe1	C9	2.043(10)	C23	C24	1.36(2)
Fe1	C10	2.038(12)	C24	C25	1.42(2)
N1	C13	1.134(11)	C31	C32	1.335(11)
C20	C2	1.444(11)	C32	C33	1.435(11)
C20	C5	1.437(11)	C32	C34	1.494(11)
C20	C11	1.435(11)	C34	C35	1.389(12)
C2	C3	1.423(13)	C34	C39	1.391(12)
C3	C4	1.413(15)	C35	C36	1.372(12)
C4	C5	1.407(12)	C36	C37	1.386(15)
C6	C7	1.36(2)	C36	C40	1.487(16)
C6	C10	1.42(2)	C37	C38	1.362(18)
C7	C8	1.439(18)	C38	C39	1.377(15)
C8	C9	1.36(2)	C40	F4	1.31(2)
C9	C10	1.468(19)	C40	F5	1.24(3)
C11	C12	1.351(10)	C40	F6	1.39(2)
C12	C13	1.438(10)	C40	F6A	1.37(2)
C12	C14	1.475(11)	C40	F4A	1.302(17)
C14	C15	1.380(13)	C40	F5A	1.25(2)
C14	C19	1.398(12)	F4	F6	1.29(6)
C15	C16	1.364(13)	F5	F6	0.87(5)
C16	C17	1.365(16)	F6A	F4A	1.46(3)
C16	C1	1.476(16)	F6A	F5A	1.54(5)
C17	C18	1.362(18)	F1	C1	1.294(19)
C18	C19	1.408(16)	C1	F3	1.252(16)
Fe2	C21	2.030(10)	C1	F2	1.261(19)

Fe2	C22	1.992(11)	C26	C27	1.33(3)
Fe2	C23	1.996(11)	C26	C30	1.45(3)
Fe2	C24	2.030(13)	C27	C28	1.40(3)
Fe2	C25	2.000(12)	C29	C28	1.41(3)
Fe2	C26	2.037(15)	C29	C30	1.39(3)
Fe2	C27	2.000(16)			

Table S8. Bond Angles for (Z)-27.

Atom	Atom	Atom	Angle/°	Atom	Atom	Atom	Angle/°
C20	Fe1	C4	69.0(3)	C23	Fe2	C26	123.4(8)
C20	Fe1	C7	123.2(4)	C23	Fe2	C27	105.9(9)
C20	Fe1	C9	160.3(5)	C23	Fe2	C29	153.6(11)
C2	Fe1	C20	41.9(3)	C23	Fe2	C28	117.7(10)
C2	Fe1	C3	41.4(4)	C24	Fe2	C26	108.0(9)
C2	Fe1	C4	69.2(4)	C24	Fe2	C29	166.3(12)
C2	Fe1	C5	69.8(4)	C25	Fe2	C21	41.1(4)
C2	Fe1	C6	121.5(6)	C25	Fe2	C24	41.4(6)
C2	Fe1	C7	106.1(5)	C25	Fe2	C26	121.7(11)
C2	Fe1	C8	121.9(5)	C25	Fe2	C27	153.6(7)
C2	Fe1	C9	156.6(5)	C25	Fe2	C29	127.9(9)
C2	Fe1	C10	158.4(6)	C25	Fe2	C28	164.9(7)
C3	Fe1	C20	69.5(3)	C27	Fe2	C21	163.8(9)
C3	Fe1	C4	40.5(4)	C27	Fe2	C24	118.1(7)
C3	Fe1	C6	156.6(7)	C27	Fe2	C26	38.4(9)
C3	Fe1	C7	121.4(6)	C27	Fe2	C29	68.3(8)
C3	Fe1	C8	106.0(5)	C27	Fe2	C28	40.9(8)
C3	Fe1	C9	121.0(5)	C29	Fe2	C26	68.9(9)
C3	Fe1	C10	159.7(7)	C28	Fe2	C21	125.8(8)
C4	Fe1	C7	157.2(6)	C28	Fe2	C24	151.5(8)
C5	Fe1	C20	41.3(3)	C28	Fe2	C26	67.7(12)
C5	Fe1	C3	68.6(4)	C28	Fe2	C29	41.0(9)
C5	Fe1	C4	40.4(3)	C30	Fe2	C21	119.8(7)
C5	Fe1	C6	126.3(5)	C30	Fe2	C22	154.6(9)
C5	Fe1	C7	160.8(5)	C30	Fe2	C23	163.3(11)
C5	Fe1	C8	156.9(4)	C30	Fe2	C24	128.5(13)
C5	Fe1	C9	123.4(5)	C30	Fe2	C25	109.5(10)
C5	Fe1	C10	109.0(5)	C30	Fe2	C26	42.3(9)
C6	Fe1	C20	108.5(4)	C30	Fe2	C27	67.9(10)
C6	Fe1	C4	162.3(6)	C30	Fe2	C29	40.4(10)
C6	Fe1	C7	38.8(6)	C30	Fe2	C28	68.5(13)
C6	Fe1	C8	67.4(5)	C22	C21	Fe2	67.8(6)
C6	Fe1	C9	68.8(6)	C25	C21	Fe2	68.3(6)

C6	Fe1	C10	40.9(7)	C25	C21	C22	104.2(10)
C8	Fe1	C20	159.9(5)	C25	C21	C31	131.5(11)
C8	Fe1	C4	121.1(4)	C31	C21	Fe2	123.7(7)
C8	Fe1	C7	41.2(5)	C31	C21	C22	124.1(10)
C8	Fe1	C9	38.8(6)	C21	C22	Fe2	70.7(6)
C9	Fe1	C4	107.3(5)	C23	C22	Fe2	69.4(6)
C9	Fe1	C7	67.8(6)	C23	C22	C21	110.0(14)
C10	Fe1	C20	122.9(5)	C22	C23	Fe2	69.1(7)
C10	Fe1	C4	124.7(7)	C24	C23	Fe2	71.7(8)
C10	Fe1	C7	67.6(7)	C24	C23	C22	107.9(13)
C10	Fe1	C8	67.9(6)	C23	C24	Fe2	68.9(8)
C10	Fe1	C9	42.2(6)	C23	C24	C25	108.3(12)
C2	C20	Fe1	67.2(5)	C25	C24	Fe2	68.2(7)
C5	C20	Fe1	68.7(4)	C21	C25	Fe2	70.6(6)
C5	C20	C2	105.9(7)	C21	C25	C24	109.5(14)
C11	C20	Fe1	122.0(5)	C24	C25	Fe2	70.5(8)
C11	C20	C2	121.5(7)	C32	C31	C21	130.8(8)
C11	C20	C5	132.1(7)	C31	C32	C33	119.6(7)
C20	C2	Fe1	70.9(5)	C31	C32	C34	125.7(6)
C3	C2	Fe1	70.9(6)	C33	C32	C34	114.7(7)
C3	C2	C20	108.4(8)	N2	C33	C32	178.8(11)
C2	C3	Fe1	67.8(5)	C35	C34	C32	120.9(8)
C4	C3	Fe1	70.3(5)	C35	C34	C39	118.3(8)
C4	C3	C2	108.0(8)	C39	C34	C32	120.8(8)
C3	C4	Fe1	69.2(5)	C36	C35	C34	120.2(9)
C5	C4	Fe1	69.0(5)	C35	C36	C37	121.5(9)
C5	C4	C3	108.5(8)	C35	C36	C40	119.6(9)
C20	C5	Fe1	69.9(4)	C37	C36	C40	118.9(9)
C4	C5	Fe1	70.6(4)	C38	C37	C36	118.1(9)
C4	C5	C20	109.1(7)	C37	C38	C39	121.7(10)
C7	C6	Fe1	71.1(7)	C38	C39	C34	120.3(9)
C7	C6	C10	109.7(12)	F4	C40	C36	114.9(14)
C10	C6	Fe1	69.7(7)	F4	C40	F6	57(3)
C6	C7	Fe1	70.0(7)	F5	C40	C36	116.5(18)
C6	C7	C8	107.7(14)	F5	C40	F4	91(3)
C8	C7	Fe1	69.0(6)	F5	C40	F6	38(2)
C7	C8	Fe1	69.8(6)	F6	C40	C36	111.1(13)
C9	C8	Fe1	70.8(7)	F6A	C40	C36	109.6(15)
C9	C8	C7	109.4(13)	F4A	C40	C36	114.1(13)

C8	C9	Fe1	70.4(6)	F4A	C40	F6A	66.3(17)
C8	C9	C10	107.4(13)	F5A	C40	C36	111.3(16)
C10	C9	Fe1	68.7(6)	F5A	C40	F6A	72(3)
C6	C10	Fe1	69.5(7)	F5A	C40	F4A	126(3)
C6	C10	C9	105.7(12)	F6	F4	C40	64.3(19)
C9	C10	Fe1	69.1(6)	F6	F5	C40	80(3)
C12	C11	C20	130.2(6)	F4	F6	C40	58.4(18)
C11	C12	C13	118.3(7)	F5	F6	C40	62(3)
C11	C12	C14	124.2(7)	F5	F6	F4	112(4)
C13	C12	C14	117.5(7)	C40	F6A	F4A	54.6(12)
N1	C13	C12	177.2(10)	C40	F6A	F5A	50.7(13)
C15	C14	C12	122.7(8)	F4A	F6A	F5A	99(2)
C15	C14	C19	117.2(8)	C40	F4A	F6A	59.0(13)
C19	C14	C12	120.0(8)	C40	F5A	F6A	57.7(19)
C16	C15	C14	122.2(9)	F1	C1	C16	113.8(10)
C15	C16	C17	120.4(10)	F3	C1	C16	115.9(13)
C15	C16	C1	120.3(10)	F3	C1	F1	100.7(16)
C17	C16	C1	119.3(10)	F3	C1	F2	109.9(15)
C18	C17	C16	119.9(9)	F2	C1	C16	112.8(14)
C17	C18	C19	120.1(10)	F2	C1	F1	102.3(17)
C14	C19	C18	120.1(10)	C27	C26	Fe2	69.3(10)
C21	Fe2	C24	69.6(4)	C27	C26	C30	106(2)
C21	Fe2	C26	156.4(10)	C30	C26	Fe2	67.0(9)
C21	Fe2	C29	107.6(6)	C26	C27	Fe2	72.4(12)
C22	Fe2	C21	41.5(5)	C26	C27	C28	111(2)
C22	Fe2	C23	41.5(5)	C28	C27	Fe2	69.6(10)
C22	Fe2	C24	67.7(7)	C28	C29	Fe2	68.3(10)
C22	Fe2	C25	68.3(7)	C30	C29	Fe2	68.0(9)
C22	Fe2	C26	160.9(10)	C30	C29	C28	106.7(18)
C22	Fe2	C27	125.7(10)	C27	C28	Fe2	69.5(10)
C22	Fe2	C29	119.7(9)	C27	C28	C29	107(2)
C22	Fe2	C28	106.7(10)	C29	C28	Fe2	70.8(10)
C23	Fe2	C21	70.5(5)	C26	C30	Fe2	70.7(9)
C23	Fe2	C24	39.4(7)	C29	C30	Fe2	71.7(11)
C23	Fe2	C25	68.7(8)	C29	C30	C26	108(3)

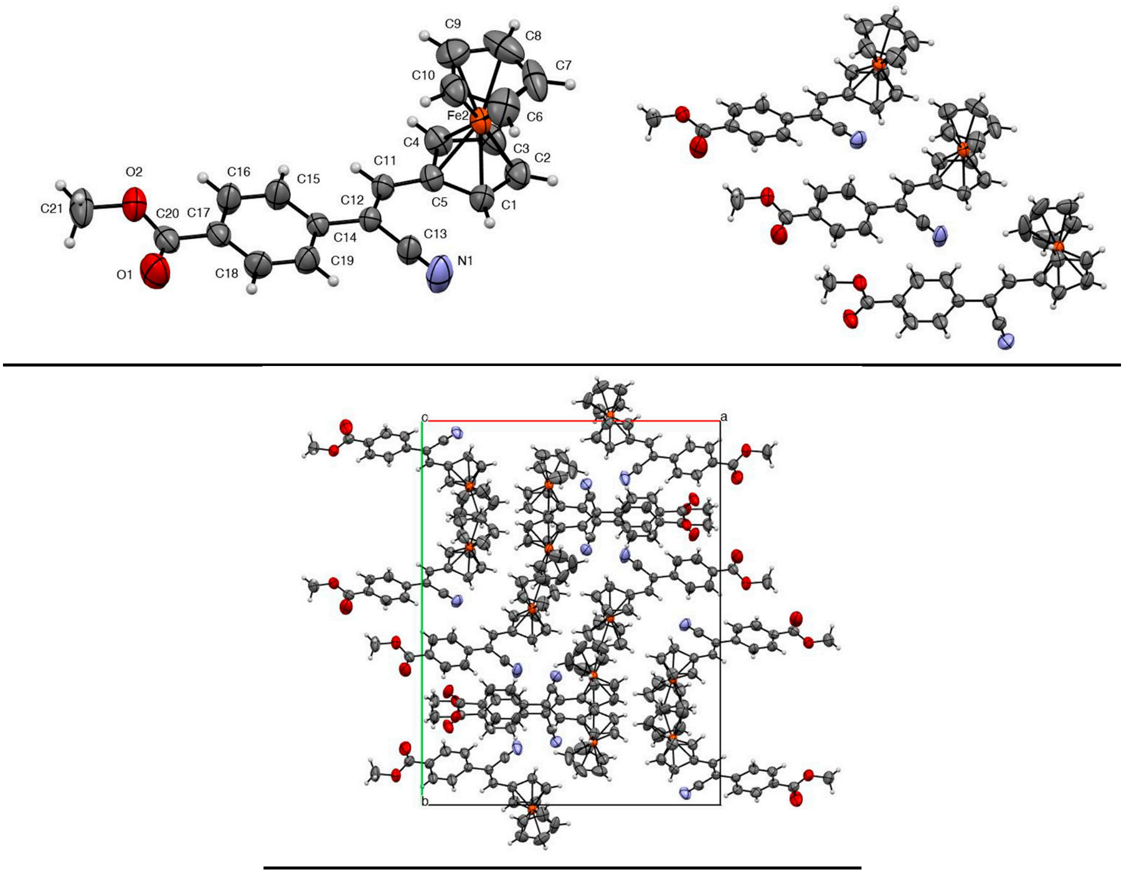

Figure S23: Crystal structure of compound (Z)-**29** with labels for non-C/H atoms and ellipsoids at the 50% probability level for (top left) a single molecular unit, (top right) the asymmetric unit, and (bottom) crystal packing along the *c* axis.

The compound crystallizes under the monoclinic space group $P2_{1/c}$. Important intermolecular interactions are observed between carbon atoms C22 and C39 indicating weak π···π stacking; C29 and C30 with H24; nitrogen N2 with H11, H15, and H21a; H30 with oxygen O1; O3 with H10; and H32 with O5, highlighting a network of moderate to strong hydrogen bonds. Additionally, close H···H contacts are seen between H36···H58, H37···H58, and H10···H21c, while H42c interacts with N3, N1 with H48, and H53 with N3, along with weaker interactions involving H1 with C48 and H48, and C8 with H47, suggesting a dense network of hydrogen bonding, and van der Waals interactions that contribute to the stabilization of the molecular assembly. Important short contacts are presented in the following list (Table S10). Table S11 summarizes the crystal data and structure refinement parameters for compound (Z)-**29**. Table S12 lists the bond lengths, and Table S13 lists the bond angles observed in (Z)-**29**. The CCDC number for (Z)-**29** is 2455361.

Table S9. Important short contacts in the crystal structure of (*Z*)-29.

Atom1	Atom2	Length
C22	C39	3.391
C29	H24	2.889
C30	H24	2.731
N2	H11	2.554
N2	H15	2.577
H30	O1	2.717
O3	H10	2.548
N2	H21a	2.55
H32	O5	2.581
H36	H58	2.365
H37	H58	2.369
H42c	N3	2.733
H10	H21c	2.377
N1	H48	2.541
H1	C48	2.821
H1	H48	2.375
C8	H47	2.812
H53	N3	2.708

Table S10. Crystal data and structure refinement for (*Z*)-29.	
Identification code	DPC301_auto
Empirical formula	$C_{21}H_{17}FeNO_2$
Formula weight	371.21
Temperature/K	300(2)
Crystal system	monoclinic
Space group	$P2_1/c$
a/Å	19.5856(2)
b/Å	25.0295(3)
c/Å	10.48898(12)
α/°	90
β/°	95.6909(10)
γ/°	90
Volume/Å^3	5116.54(10)
Z	12
ρ_{calc}g/cm^3	1.446
μ/mm^{-1}	7.195
F(000)	2304
Crystal size/mm^3	0.54 × 0.28 × 0.18
Radiation	CuKα (λ = 1.54184)
2Θ range for data collection/°	4.534 to 136.946
Index ranges	$-23 \leq h \leq 23$, $-30 \leq k \leq 29$, $-12 \leq l \leq 10$
Reflections collected	27598
Independent reflections	9431 [R_{int} = 0.0387, R_{sigma} = 0.0419]
Data/restraints/parameters	9431/0/680
Goodness-of-fit on F^2	1.044
Final R indexes [I>=2σ (I)]	R_1 = 0.0458, wR_2 = 0.1112
Final R indexes [all data]	R_1 = 0.0666, wR_2 = 0.1206
Largest diff. peak/hole / e Å^{-3}	0.47/-0.39

Table S11. Bond Lengths for (*Z*)-29.

Atom	Atom	Length/Å	Atom	Atom	Length/Å
Fe1	C22	2.031(3)	C4	C5	1.435(4)
Fe1	C23	2.046(3)	C5	C11	1.446(4)
Fe1	C24	2.043(3)	C6	C7	1.409(6)
Fe1	C25	2.030(3)	C6	C10	1.377(5)
Fe1	C26	2.032(3)	C7	C8	1.392(7)
Fe1	C27	2.027(4)	C8	C9	1.412(6)
Fe1	C28	2.037(4)	C9	C10	1.392(5)
Fe1	C29	2.033(4)	C11	C12	1.351(4)
Fe1	C30	2.037(4)	C12	C13	1.438(4)
Fe1	C31	2.036(3)	C12	C14	1.482(4)
O3	C41	1.194(4)	C14	C15	1.387(4)
O4	C41	1.327(4)	C14	C19	1.380(4)
O4	C42	1.438(4)	C15	C16	1.374(4)
N2	C34	1.129(4)	C16	C17	1.378(4)
C22	C23	1.410(4)	C17	C18	1.374(4)
C22	C26	1.437(4)	C17	C20	1.483(4)
C23	C24	1.403(5)	C18	C19	1.389(4)
C24	C25	1.409(4)	Fe3	C43	2.035(3)
C25	C26	1.421(4)	Fe3	C44	2.038(3)
C26	C32	1.449(4)	Fe3	C45	2.045(3)
C27	C28	1.412(6)	Fe3	C46	2.049(3)
C27	C31	1.396(6)	Fe3	C47	2.028(3)
C28	C29	1.393(6)	Fe3	C48	2.014(4)
C29	C30	1.417(5)	Fe3	C49	2.029(4)
C30	C31	1.388(6)	Fe3	C50	2.032(4)
C32	C33	1.348(4)	Fe3	C51	2.032(4)
C33	C34	1.444(4)	Fe3	C52	2.018(4)
C33	C35	1.479(4)	O5	C62	1.198(3)
C35	C36	1.400(4)	O6	C62	1.324(4)
C35	C40	1.389(4)	O6	C63	1.445(4)
C36	C37	1.378(4)	N3	C55	1.141(4)
C37	C38	1.386(4)	C43	C44	1.440(4)
C38	C39	1.378(4)	C43	C47	1.424(4)

C38	C41	1.487(4)	C43	C53	1.448(4)
C39	C40	1.373(4)	C44	C45	1.419(5)
Fe2	C1	2.035(3)	C45	C46	1.405(5)
Fe2	C2	2.049(3)	C46	C47	1.413(4)
Fe2	C3	2.040(3)	C48	C49	1.478(8)
Fe2	C4	2.026(3)	C48	C52	1.351(8)
Fe2	C5	2.036(3)	C49	C50	1.418(6)
Fe2	C6	2.034(4)	C50	C51	1.344(6)
Fe2	C7	2.028(4)	C51	C52	1.335(7)
Fe2	C8	2.032(4)	C53	C54	1.342(4)
Fe2	C9	2.038(4)	C54	C55	1.441(4)
Fe2	C10	2.041(3)	C54	C56	1.482(4)
O1	C20	1.197(4)	C56	C57	1.372(4)
O2	C20	1.332(4)	C56	C61	1.381(4)
O2	C21	1.449(4)	C57	C58	1.384(4)
N1	C13	1.134(4)	C58	C59	1.366(4)
C1	C2	1.415(4)	C59	C60	1.372(4)
C1	C5	1.436(4)	C59	C62	1.485(4)
C2	C3	1.396(5)	C60	C61	1.380(4)
C3	C4	1.417(5)			

Table S12. Bond Angles for (*Z*)-29.

Atom	Atom	Atom	Angle/°	Atom	Atom	Atom	Angle/°
C22	Fe1	C23	40.47(12)	C1	C2	Fe2	69.21(18)
C22	Fe1	C24	68.31(13)	C3	C2	Fe2	69.70(19)
C22	Fe1	C26	41.41(11)	C3	C2	C1	108.8(3)
C22	Fe1	C28	128.12(17)	C2	C3	Fe2	70.38(19)
C22	Fe1	C29	164.78(16)	C2	C3	C4	108.3(3)
C22	Fe1	C30	153.55(15)	C4	C3	Fe2	69.07(18)
C22	Fe1	C31	120.69(16)	C3	C4	Fe2	70.14(19)
C24	Fe1	C23	40.13(13)	C3	C4	C5	108.5(3)
C25	Fe1	C22	68.51(13)	C5	C4	Fe2	69.70(17)
C25	Fe1	C23	67.70(13)	C1	C5	Fe2	69.30(18)
C25	Fe1	C24	40.48(12)	C1	C5	C11	131.0(3)
C25	Fe1	C26	40.96(12)	C4	C5	Fe2	68.95(18)
C25	Fe1	C28	151.79(16)	C4	C5	C1	106.3(3)
C25	Fe1	C29	118.12(17)	C4	C5	C11	122.6(3)
C25	Fe1	C30	107.25(16)	C11	C5	Fe2	123.1(2)
C25	Fe1	C31	127.19(15)	C7	C6	Fe2	69.5(2)
C26	Fe1	C23	68.79(12)	C10	C6	Fe2	70.5(2)
C26	Fe1	C24	68.97(12)	C10	C6	C7	108.3(4)
C26	Fe1	C28	166.32(17)	C6	C7	Fe2	69.9(2)
C26	Fe1	C29	152.15(18)	C8	C7	Fe2	70.1(2)
C26	Fe1	C30	118.06(15)	C8	C7	C6	107.8(4)
C26	Fe1	C31	108.12(14)	C7	C8	Fe2	69.8(2)
C27	Fe1	C22	109.75(17)	C7	C8	C9	107.5(4)
C27	Fe1	C23	121.09(17)	C9	C8	Fe2	69.9(2)
C27	Fe1	C24	153.63(17)	C8	C9	Fe2	69.5(2)
C27	Fe1	C25	165.38(16)	C10	C9	Fe2	70.2(2)
C27	Fe1	C26	128.14(15)	C10	C9	C8	107.9(4)
C27	Fe1	C28	40.66(16)	C6	C10	Fe2	70.0(2)
C27	Fe1	C29	67.48(19)	C6	C10	C9	108.5(3)
C27	Fe1	C30	67.43(19)	C9	C10	Fe2	69.9(2)

C27	Fe1	C31	40.17(16)	C12	C11	C5	130.1(3)
C28	Fe1	C23	108.72(15)	C11	C12	C13	120.1(3)
C28	Fe1	C24	118.41(15)	C11	C12	C14	124.1(3)
C29	Fe1	C23	126.91(15)	C13	C12	C14	115.7(3)
C29	Fe1	C24	107.11(16)	N1	C13	C12	178.6(4)
C29	Fe1	C28	40.04(17)	C15	C14	C12	121.5(3)
C29	Fe1	C30	40.75(16)	C19	C14	C12	121.1(3)
C29	Fe1	C31	67.63(16)	C19	C14	C15	117.4(3)
C30	Fe1	C23	164.09(16)	C16	C15	C14	121.7(3)
C30	Fe1	C24	126.39(17)	C15	C16	C17	120.5(3)
C30	Fe1	C28	68.10(17)	C16	C17	C20	121.6(3)
C31	Fe1	C23	155.01(17)	C18	C17	C16	118.5(3)
C31	Fe1	C24	163.93(17)	C18	C17	C20	119.9(3)
C31	Fe1	C28	68.09(15)	C17	C18	C19	120.9(3)
C31	Fe1	C30	39.85(16)	C14	C19	C18	120.9(3)
C41	O4	C42	116.7(3)	O1	C20	O2	123.7(3)
C23	C22	Fe1	70.31(18)	O1	C20	C17	124.5(3)
C23	C22	C26	108.0(3)	O2	C20	C17	111.8(3)
C26	C22	Fe1	69.31(17)	C43	Fe3	C44	41.40(11)
C22	C23	Fe1	69.22(17)	C43	Fe3	C45	68.71(12)
C24	C23	Fe1	69.83(18)	C43	Fe3	C46	68.86(13)
C24	C23	C22	108.8(3)	C44	Fe3	C45	40.67(13)
C23	C24	Fe1	70.04(18)	C44	Fe3	C46	68.54(15)
C23	C24	C25	107.7(3)	C45	Fe3	C46	40.14(14)
C25	C24	Fe1	69.29(17)	C47	Fe3	C43	41.04(12)
C24	C25	Fe1	70.24(18)	C47	Fe3	C44	68.92(14)
C24	C25	C26	109.2(3)	C47	Fe3	C45	67.94(14)
C26	C25	Fe1	69.57(17)	C47	Fe3	C46	40.57(12)
C22	C26	Fe1	69.28(17)	C47	Fe3	C49	164.5(2)
C22	C26	C32	131.5(3)	C47	Fe3	C50	151.88(16)
C25	C26	Fe1	69.47(17)	C47	Fe3	C51	118.52(17)
C25	C26	C22	106.3(3)	C48	Fe3	C43	107.28(16)
C25	C26	C32	122.1(3)	C48	Fe3	C44	122.2(2)
C32	C26	Fe1	123.4(2)	C48	Fe3	C45	158.3(3)
C28	C27	Fe1	70.0(2)	C48	Fe3	C46	160.2(3)
C31	C27	Fe1	70.3(2)	C48	Fe3	C47	124.0(2)
C31	C27	C28	108.6(4)	C48	Fe3	C49	42.9(2)
C27	C28	Fe1	69.3(2)	C48	Fe3	C50	68.54(18)
C29	C28	Fe1	69.8(2)	C48	Fe3	C51	66.3(2)

C29	C28	C27	107.0(4)	C48	Fe3	C52	39.2(2)
C28	C29	Fe1	70.1(2)	C49	Fe3	C43	127.51(19)
C28	C29	C30	108.5(4)	C49	Fe3	C44	109.36(17)
C30	C29	Fe1	69.8(2)	C49	Fe3	C45	121.5(2)
C29	C30	Fe1	69.5(2)	C49	Fe3	C46	154.4(2)
C31	C30	Fe1	70.1(2)	C49	Fe3	C50	40.87(18)
C31	C30	C29	107.6(4)	C49	Fe3	C51	67.27(19)
C27	C31	Fe1	69.6(2)	C50	Fe3	C43	166.70(16)
C30	C31	Fe1	70.1(2)	C50	Fe3	C44	129.19(15)
C30	C31	C27	108.2(4)	C50	Fe3	C45	110.19(15)
C33	C32	C26	129.8(3)	C50	Fe3	C46	119.46(15)
C32	C33	C34	118.9(3)	C50	Fe3	C51	38.62(16)
C32	C33	C35	125.3(2)	C51	Fe3	C43	152.59(17)
C34	C33	C35	115.8(2)	C51	Fe3	C44	164.70(17)
N2	C34	C33	178.8(4)	C51	Fe3	C45	127.11(18)
C36	C35	C33	121.2(3)	C51	Fe3	C46	107.65(18)
C40	C35	C33	121.5(3)	C52	Fe3	C43	119.71(17)
C40	C35	C36	117.2(3)	C52	Fe3	C44	155.8(2)
C37	C36	C35	120.6(3)	C52	Fe3	C45	161.7(2)
C36	C37	C38	121.4(3)	C52	Fe3	C46	124.6(2)
C37	C38	C41	122.2(3)	C52	Fe3	C47	106.82(19)
C39	C38	C37	118.2(3)	C52	Fe3	C49	68.0(2)
C39	C38	C41	119.7(3)	C52	Fe3	C50	65.61(18)
C40	C39	C38	120.9(3)	C52	Fe3	C51	38.49(19)
C39	C40	C35	121.8(3)	C62	O6	C63	116.3(2)
O3	C41	O4	123.2(3)	C44	C43	Fe3	69.39(17)
O3	C41	C38	124.5(3)	C44	C43	C53	130.9(3)
O4	C41	C38	112.2(3)	C47	C43	Fe3	69.20(17)
C1	Fe2	C2	40.53(12)	C47	C43	C44	106.9(3)
C1	Fe2	C3	68.23(14)	C47	C43	C53	122.1(3)
C1	Fe2	C5	41.31(12)	C53	C43	Fe3	123.1(2)
C1	Fe2	C9	155.95(15)	C43	C44	Fe3	69.21(18)
C1	Fe2	C10	121.00(15)	C45	C44	Fe3	69.9(2)
C3	Fe2	C2	39.92(14)	C45	C44	C43	107.3(3)
C3	Fe2	C10	164.03(16)	C44	C45	Fe3	69.38(18)
C4	Fe2	C1	68.87(14)	C46	C45	Fe3	70.09(19)
C4	Fe2	C2	68.03(14)	C46	C45	C44	109.2(3)
C4	Fe2	C3	40.79(13)	C45	C46	Fe3	69.77(19)
C4	Fe2	C5	41.35(12)	C45	C46	C47	107.7(3)

C4	Fe2	C6	163.17(15)	C47	C46	Fe3	68.90(18)
C4	Fe2	C7	154.89(17)	C43	C47	Fe3	69.77(17)
C4	Fe2	C8	120.86(19)	C46	C47	Fe3	70.53(18)
C4	Fe2	C9	109.05(17)	C46	C47	C43	108.9(3)
C4	Fe2	C10	127.06(14)	C49	C48	Fe3	69.1(2)
C5	Fe2	C2	68.84(12)	C52	C48	Fe3	70.6(2)
C5	Fe2	C3	69.17(13)	C52	C48	C49	106.3(4)
C5	Fe2	C9	121.05(15)	C48	C49	Fe3	68.0(2)
C5	Fe2	C10	108.32(13)	C50	C49	Fe3	69.6(2)
C6	Fe2	C1	107.65(17)	C50	C49	C48	103.7(4)
C6	Fe2	C2	120.75(16)	C49	C50	Fe3	69.5(2)
C6	Fe2	C3	154.74(16)	C51	C50	Fe3	70.7(2)
C6	Fe2	C5	125.24(16)	C51	C50	C49	109.1(4)
C6	Fe2	C9	67.00(18)	C50	C51	Fe3	70.7(2)
C6	Fe2	C10	39.49(15)	C52	C51	Fe3	70.2(3)
C7	Fe2	C1	124.74(19)	C52	C51	C50	110.0(5)
C7	Fe2	C2	107.41(16)	C48	C52	Fe3	70.2(3)
C7	Fe2	C3	119.81(15)	C51	C52	Fe3	71.3(2)
C7	Fe2	C5	162.28(19)	C51	C52	C48	111.0(5)
C7	Fe2	C6	40.60(17)	C54	C53	C43	131.0(3)
C7	Fe2	C8	40.10(19)	C53	C54	C55	119.4(3)
C7	Fe2	C9	67.56(18)	C53	C54	C56	125.2(3)
C7	Fe2	C10	67.41(14)	C55	C54	C56	115.4(3)
C8	Fe2	C1	161.44(19)	N3	C55	C54	178.3(4)
C8	Fe2	C2	125.00(17)	C57	C56	C54	121.3(3)
C8	Fe2	C3	107.85(16)	C57	C56	C61	117.2(3)
C8	Fe2	C5	156.1(2)	C61	C56	C54	121.5(3)
C8	Fe2	C6	67.68(19)	C56	C57	C58	121.5(3)
C8	Fe2	C9	40.61(18)	C59	C58	C57	121.0(3)
C8	Fe2	C10	67.66(16)	C58	C59	C60	117.9(3)
C9	Fe2	C2	162.74(16)	C58	C59	C62	119.2(3)
C9	Fe2	C3	126.83(17)	C60	C59	C62	122.8(3)
C9	Fe2	C10	39.92(15)	C59	C60	C61	121.1(3)
C10	Fe2	C2	155.25(16)	C60	C61	C56	121.2(3)
C20	O2	C21	116.5(3)	O5	C62	O6	123.2(3)
C2	C1	Fe2	70.3(2)	O5	C62	C59	124.7(3)
C2	C1	C5	108.2(3)	O6	C62	C59	112.0(2)

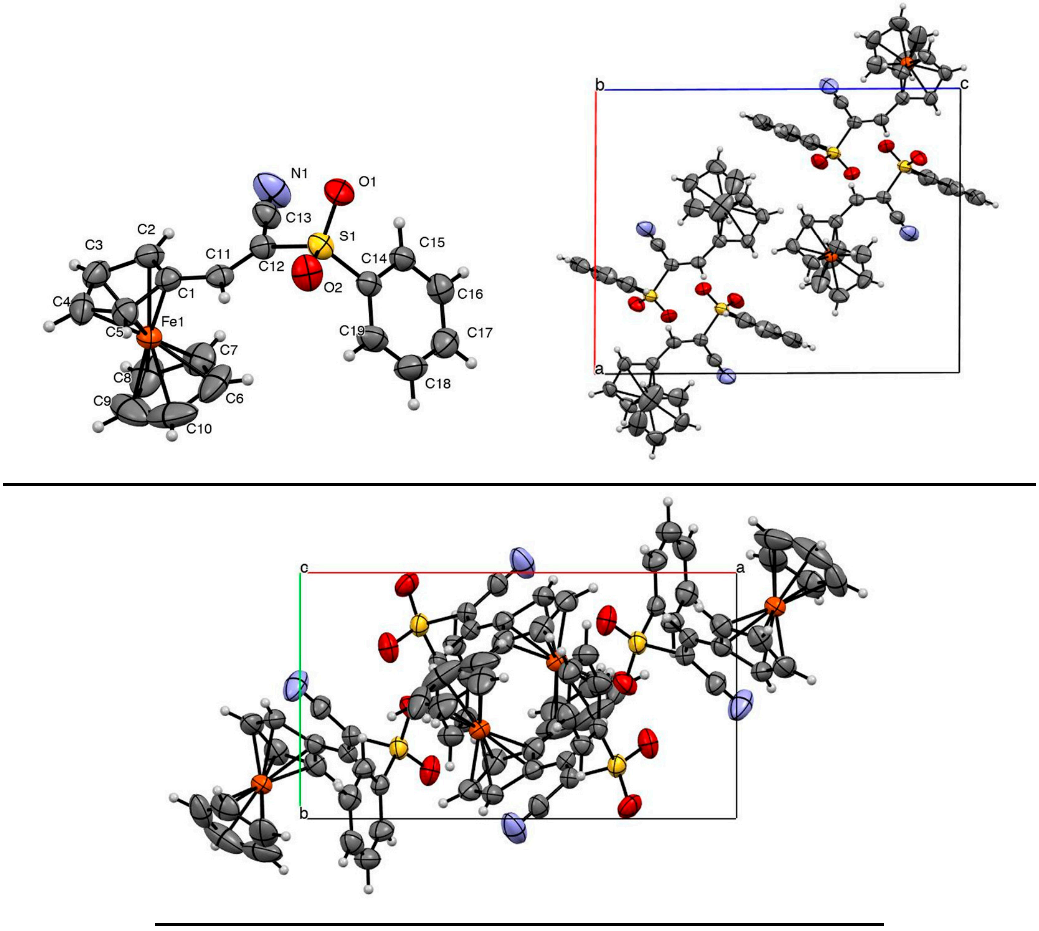

Figure S24: Crystal structure of compound (*E*)-**33** with labels for non-C/H atoms and ellipsoids at the 50% probability level for (top left) a single molecular unit, (top right) the asymmetric unit, and (bottom) crystal packing along the *c* axis.

The compound crystallizes under the monoclinic space group $P2_{1/n}$. Important intermolecular interactions are observed between hydrogen H18 and oxygen O1; H8 and nitrogen N1; H5 and O1; H11 and O1; as well as weaker contacts between H19 and carbon C11, and H3 with carbon C16, indicating a network of moderate hydrogen bonds, particularly involving O1 as a common acceptor, along with weak C–H···C interactions contributing to the stabilization of the crystal structure. Important short contacts are presented in the following list (Table S14). Table S15 summarizes the crystal data and structure refinement parameters for compound (*E*)-**33**. Table S16 lists the bond lengths, and Table S17 lists the bond angles observed in (*E*)-**33**. The CCDC number for (*E*)-**33** is 2455359.

Table S13. Important short contacts in the crystal structure (*E*)-33.

Atom1	Atom2	Length
H18	O1	2.619
H8	N1	2.688
H5	O1	2.695
H11	O1	2.542
H19	C11	2.884
H3	C16	2.878

Table S14. Crystal data and structure refinement for (*E*)-33.	
Identification code	dpc307_auto
Empirical formula	$C_{19}H_{15}FeNO_2S$
Formula weight	377.23
Temperature/K	300(2)
Crystal system	monoclinic
Space group	$P2_1/n$
a/Å	13.1731(3)
b/Å	7.41671(18)
c/Å	16.9253(4)
α/°	90
β/°	90.5143(18)
γ/°	90
Volume/Å^3	1653.55(7)
Z	4
ρ_{calc}g/cm^3	1.515
μ/mm^{-1}	8.58
F(000)	776
Crystal size/mm^3	0.24 × 0.14 × 0.01
Radiation	Cu Kα (λ = 1.54184)
2Θ range for data collection/°	8.46 to 136.82
Index ranges	$-15 \leq h \leq 14$, $-8 \leq k \leq 8$, $-20 \leq l \leq 18$
Reflections collected	7946
Independent reflections	3029 [R_{int} = 0.0367, R_{sigma} = 0.0478]
Data/restraints/parameters	3029/4/218
Goodness-of-fit on F^2	1.028
Final R indexes [I>=2σ (I)]	R_1 = 0.0425, wR_2 = 0.1115
Final R indexes [all data]	R_1 = 0.0507, wR_2 = 0.1181
Largest diff. peak/hole / e Å^{-3}	0.35/-0.49

Table S15. Bond Lengths for (*E*)-33.					
**Atom**	**Atom**	**Length/Å**	**Atom**	**Atom**	**Length/Å**
Fe1	C1	2.028(3)	C1	C11	1.425(4)
Fe1	C2	2.028(3)	C2	C3	1.411(5)
Fe1	C3	2.050(3)	C3	C4	1.408(5)
Fe1	C4	2.055(3)	C4	C5	1.405(5)
Fe1	C5	2.033(3)	C6	C7	1.396(6)
Fe1	C6	2.027(4)	C6	C10	1.386(7)
Fe1	C7	2.036(3)	C7	C8	1.411(6)
Fe1	C8	2.035(4)	C8	C9	1.361(8)
Fe1	C9	2.023(5)	C9	C10	1.371(9)
Fe1	C10	2.027(5)	C11	C12	1.351(4)
S1	O1	1.435(2)	C12	C13	1.413(4)
S1	O2	1.436(2)	C14	C15	1.382(4)
S1	C12	1.771(3)	C14	C19	1.391(4)
S1	C14	1.758(3)	C15	C16	1.377(5)
N1	C13	1.137(4)	C16	C17	1.380(5)
C1	C2	1.433(4)	C17	C18	1.374(6)
C1	C5	1.449(4)	C18	C19	1.372(5)

Table S16. Bond Angles for (*E*)-33.							
**Atom**	**Atom**	**Atom**	**Angle/°**	**Atom**	**Atom**	**Atom**	**Angle/°**
C2	Fe1	C1	41.38(13)	C14	S1	O2	109.16(15)
C3	Fe1	C1	68.57(13)	C14	S1	C12	105.37(13)
C3	Fe1	C2	40.48(14)	C2	C1	Fe1	69.32(18)
C4	Fe1	C1	68.96(13)	C5	C1	Fe1	69.30(18)
C4	Fe1	C2	68.51(15)	C5	C1	C2	106.7(3)
C4	Fe1	C3	40.13(16)	C11	C1	Fe1	121.9(2)
C5	Fe1	C1	41.81(13)	C11	C1	C2	130.1(3)
C5	Fe1	C2	69.40(14)	C11	C1	C5	122.9(3)
C5	Fe1	C3	67.86(15)	C1	C2	Fe1	69.30(18)
C5	Fe1	C4	40.20(13)	C3	C2	Fe1	70.59(19)
C6	Fe1	C1	108.81(17)	C3	C2	C1	107.7(3)
C6	Fe1	C2	125.2(2)	C2	C3	Fe1	68.93(18)
C6	Fe1	C3	161.2(2)	C4	C3	Fe1	70.12(19)
C6	Fe1	C4	158.0(2)	C4	C3	C2	109.2(3)
C6	Fe1	C5	123.48(17)	C3	C4	Fe1	69.7(2)
C7	Fe1	C1	122.50(15)	C5	C4	Fe1	69.08(18)
C7	Fe1	C2	107.89(17)	C5	C4	C3	108.2(3)
C7	Fe1	C3	124.27(18)	C1	C5	Fe1	68.88(17)
C7	Fe1	C4	159.61(17)	C4	C5	Fe1	70.73(19)
C7	Fe1	C5	159.19(16)	C4	C5	C1	108.1(3)
C7	Fe1	C6	40.20(19)	C7	C6	Fe1	70.2(2)
C8	Fe1	C1	158.27(19)	C10	C6	Fe1	70.0(3)
C8	Fe1	C2	121.9(2)	C10	C6	C7	108.1(5)
C8	Fe1	C3	107.79(18)	C6	C7	Fe1	69.6(2)
C8	Fe1	C4	122.97(16)	C8	C7	Fe1	69.7(2)
C8	Fe1	C5	158.50(18)	C8	C7	C6	106.6(4)
C8	Fe1	C6	67.32(19)	C7	C8	Fe1	69.7(2)
C8	Fe1	C7	40.57(18)	C9	C8	Fe1	69.9(3)
C9	Fe1	C1	161.0(2)	C9	C8	C7	107.9(5)
C9	Fe1	C2	156.5(3)	C8	C9	Fe1	70.9(3)
C9	Fe1	C3	121.8(2)	C10	C9	Fe1	70.3(3)
C9	Fe1	C4	107.78(19)	C10	C9	C8	109.5(5)
C9	Fe1	C5	123.7(2)	C6	C10	Fe1	70.0(3)

C9	Fe1	C6	66.8(2)	C9	C10	Fe1	70.1(3)
C9	Fe1	C7	67.0(2)	C9	C10	C6	107.9(5)
C9	Fe1	C8	39.2(2)	C12	C11	C1	128.9(3)
C10	Fe1	C1	125.2(2)	C11	C12	S1	119.9(2)
C10	Fe1	C2	162.0(3)	C13	C12	S1	114.3(2)
C10	Fe1	C3	156.7(2)	C13	C12	C11	125.7(3)
C10	Fe1	C4	122.2(2)	C12	C13	N1	176.9(4)
C10	Fe1	C5	108.5(2)	C15	C14	S1	119.5(2)
C10	Fe1	C6	40.0(2)	C19	C14	S1	119.5(3)
C10	Fe1	C7	67.3(2)	C19	C14	C15	121.0(3)
C10	Fe1	C8	66.6(2)	C16	C15	C14	119.1(3)
C10	Fe1	C9	39.6(3)	C17	C16	C15	120.1(3)
O2	S1	O1	119.57(15)	C18	C17	C16	120.3(4)
C12	S1	O1	106.90(15)	C19	C18	C17	120.6(3)
C12	S1	O2	107.28(14)	C18	C19	C14	118.9(3)
C14	S1	O1	107.67(14)				
